# Disentangling biological variability and taphonomy: shape analysis of the limb long bones of the sauropodomorph dinosaur *Plateosaurus*

**DOI:** 10.7717/peerj.9359

**Published:** 2020-07-23

**Authors:** Rémi Lefebvre, Ronan Allain, Alexandra Houssaye, Raphaël Cornette

**Affiliations:** 1 Mécanismes Adaptatifs et Évolution, UMR 7179, MNHN, CNRS, Muséum National d’Histoire Naturelle, Paris, France; 2 Centre de Recherche en Paléontologie - Paris, UMR 7207, MNHN, CNRS, SU, Muséum National d’Histoire Naturelle, Paris, France; 3 Institut de Systématique, Évolution, Biodiversité, UMR7205, MNHN, CNRS, SU, EPHE, UA, Muséum National d’Histoire Naturelle, Paris, France

**Keywords:** Dinosauria, Sauropodomorpha, 3D Geometric morphometrics, Deformation, Comparative anatomy, Paleobiology, Saurischia

## Abstract

Sauropodomorph dinosaurs constitute a well-studied clade of dinosaurs, notably because of the acquisition of gigantism within this group. The genus *Plateosaurus* is one of the best-known sauropodomorphs, with numerous remains from various localities. Its tumultuous taxonomic history suggests the relevance of addressing its intrageneric shape variability, mixed with taphonomic modifications of the original bone shape. Here we investigate quantitatively the morphological variation of *Plateosaurus* occurring at the genus level by studying the shape variation of a sample of limb long bones. By means of 3D geometric morphometrics, the analysis of the uncorrelated variation permits separation of the variation estimated as obviously taphonomically influenced from the more biologically plausible variation. Beyond the dominant taphonomic signal, our approach permits interpretation of the most biologically plausible features, even on anatomical parts influenced by taphonomic deformations. Those features are thus found on a quantitative basis from the variation of samples containing fossil specimens, by taking the impact of taphonomy into account, which is paramount in order to avoid making biologically ambiguous interpretations.

## Introduction

The evolutionary history of sauropodomorph dinosaurs is strongly linked to the increase of body size and mass, from relatively small bipedal early representatives to gigantic organisms reaching several dozen tons, implying strong variation in the appendicular skeleton of these animals (Carrano, 2005; Sander & Clauss, 2008; Langer et al., 2010; Rauhut et al., 2011; Sander et al., 2011).

One of the best-known bipedal representatives of the group is the genus *Plateosaurus* von Meyer, 1837. Indeed, this non-sauropod sauropodomorph (“prosauropod”) is known from numerous skeletons mainly coming from Germany, Switzerland and France (von Huene, 1926, 1932; Weishampel & Westphal, 1986; Sander, 1992; Moser, 2003; Hofmann & Sander, 2014). Many studies investigated the paleobiology of *Plateosaurus*, focusing on sexual dimorphism (Weishampel & Chapman, 1990; Galton, 1997), functional morphology (Bonnan & Senter, 2007; Mallison, 2010a, 2010b), paleohistology (Sander & Klein, 2005; Cerda et al., 2017) and developmental biology (Böhmer, Rauhut & Wörheide, 2015).

However, the taxonomic history of this genus is tumultuous (Weishampel & Chapman, 1990; Galton, 2001a, 2001b; Moser, 2003; Yates, 2003; Prieto-Márquez & Norell, 2011; Galton, 2012). Since the discovery of the first remains until the middle of the twentieth century, a dozen species classified in several different genera have been erected (von Huene, 1905; Fraas, 1913; Weishampel & Chapman, 1990, Table 3.1). During the second half of the same century, taxonomic studies synonymized most of the species (Galton, 1985, 2001b).

This complex taxonomic history raises the question of the intrageneric variation of *Plateosaurus*, often difficult to estimate in the fossil record. The use of quantitative methods such as three dimensional geometric morphometrics (3D GM; Rohlf & Marcus, 1993), can give an overview of the morphological variation of bones within a genus (Cordeiro-Estrela et al., 2008; Hautier et al., 2017). This tool is also widely used in morphofunctional studies on the appendicular skeleton (Milne, Vizcaíno & Fernicola, 2009; Martín-Serra, Figueirido & Palmqvist, 2014; Botton-Divet et al., 2016; Maclaren et al., 2018) but remains little used in non-avian dinosaur studies (Hedrick & Dodson, 2013; Ratsimbaholison, Felice & O’Connor, 2016; Neenan et al., 2018). By using anatomical landmarks and sliding semilandmarks of curves and surface (Gunz & Mitteroecker, 2013), 3D GM permits one to quantify, analyze and visualize the morphological variation in a sample by taking into account the overall shape of the bones of interest, even with few anatomically homologous landmarks, such as in limb long bones. By the means of 3D GM, it is possible to thoroughly investigate the shape variation of these bones, given their critical importance in phylogenetic (Peyre de Fabrègues, Allain & Barriel, 2015) and morphofunctional studies (Bonnan, 2007).

The description of the range of variation within a genus is of particular importance to appreciate the potential characteristics delimiting species. It also permits appreciation of the intraspecific biological variability occurring in a sample, such as ontogenetic variation or dimorphism/polymorphism. If high developmental plasticity has been histologically detected for *Plateosaurus* (Sander & Klein, 2005), too few quantitative studies investigated the intrageneric appendicular variation of this genus (Weishampel & Chapman, 1990). However, shape quantification using 3D GM is limited by the taphonomic history of the fossils. Indeed, taphonomy (i.e., deformations occurring between the death of the organism and the discovery of the fossil remains) involves various minor to heavy modifications of the original conformation of the remains, so that even paired bones from the same individual can show different taphonomic influence (Müller et al., 2018; Hedrick et al., 2019). Examining the impact of taphonomy is thus critical in qualitative and quantitative studies to avoid misleading conclusions on fossil organisms (Hedrick & Dodson, 2013; Tschopp, Russo & Dzemski, 2013; Baert, Burns & Currie, 2014; Müller et al., 2018; Hedrick et al., 2019).

Here we investigate the biological variability of a sample of sauropodomorph stylopod and zeugopod limb bones, for the first-time using 3D GM and taking into account the taphonomic influence on their morphology, through the case study of the genus *Plateosaurus*. Besides studying the shape variability occurring in limb bones within the genus, we also consider the variability occurring in the historically related genera of German non-sauropod sauropodomorphs (i.e., *Efraasia* and *Ruehleia*; see Material). Since these taxa are supposedly distinct, we expect to find biological intergeneric variation separating them. Though relatively limited because of the similarity of these genera, this intergeneric variation should be higher than the biological variation obtained within the *Plateosaurus* sample. The detection of such a signal should corroborate the taxonomic delimitation of these genera.

Through describing an intrageneric range of variation often poorly illustrated in dinosaur studies and by taking taphonomy into account, we aim to highlight with more confidence the biologically driven variation.

## Materials and Methods

### Material

We studied the stylopod (i.e., humerus and femur) and zeugopod (i.e., radius, ulna, tibia and fibula) bones of *Plateosaurus*. Among a fossil sample constituted of numerous but often incomplete long bones, a set of 67 bones (11 humeri, 11 radii, 12 ulnae, 10 femora, 10 tibiae and 13 fibulae) out of a total of 140 examined bones (25 humeri, 19 radii, 19 ulnae, 39 femora, 30 tibiae, 24 fibulae) housed in the German collections from the Staatliches Museum für Naturkunde, Stuttgart (SMNS), Institute for Geosciences, Eberhard-Karls-Universität, Tübingen (GPIT) and Museum für Naturkunde, Berlin (MB.R) have been investigated in this study (Table 1). Only the bones that preserved all the morphological features captured by the anatomical landmarks datasets were included in this study. No particular selection was done based on the apparent taphonomic deformation of the specimens. The investigated material comes from several localities (see Table 1). For *Plateosaurus*, the majority of the bones comes from the Trossingen locality (Sander, 1992). The remaining material comes from the following localities: One humerus and one femur come from Stuttgart–Degerloch; one ulna from Pfrondorf; three tibiae respectively coming from Tübingen, Halberstadt and Erlenberg; and one fibula is without information about the locality. For *Efraasia*, all the material comes from Pfaffenhofen, except one humerus coming from Oschenbach. For *Ruehleia*, all the material come from Römhild. The femur of SMNS 12220 (unclear taxonomy, see below) comes from Pfaffenhofen. Presently, if the type species *P. engelhardti* is recognized, the taxonomic attribution of the material coming from Trossingen and some other localities is still debated (Galton, 2001b; Moser, 2003; Prieto-Márquez & Norell, 2011; Galton, 2012). Consequently, all the material studied here is simply considered, for the scope of our study, as belonging to the genus *Plateosaurus*. *Plateosaurus gracilis* is the only other widely recognized species (Yates, 2003), although it could be a metataxon, that is, an assemblage of operating taxonomic units lacking positive evidence of monophyly or paraphyly (Archibald, 1994). This species is, however, not sampled in our study, because no investigated bone was sufficiently preserved for the analysis. Finally, two other genera of German non-sauropodan sauropodmorphs were recently erected: *Ruehleia bedheimensis* from the Norian of Römhild (South Thuringia, Germany) is based on material previously referred to *Plateosaurus* (Galton, 2001a, 2001b). *Efraasia minor* is based on part of the material previously referred to *Sellosaurus gracilis* (now invalid), while some material from the other parts is referred to *Plateosaurus gracilis* (Yates, 2003). The remaining material, including the femur SMNS 12220 sampled in our study, however, has not been attributed to either of these two taxa.

**Table 1:** Material sampled in this study.

Collection number	Genus	Locality	Orientation	Data acquisition	Maximum length (cm)
**Humerus**					
SMNS 12949	*Plateosaurus*	Trossingen	L	Artec EVA Surface Scanner	41.4
SMNS 91296 (F10) #1	*Plateosaurus*	Trossingen	L	Artec EVA Surface Scanner	45.4
SMNS 91296 (F10) #2	*Plateosaurus*	Trossingen	L	Artec EVA Surface Scanner	38
SMNS 91296 (F10) #3	*Plateosaurus*	Trossingen	R	Artec EVA Surface Scanner	36.6
SMNS 91296 (F10) #4	*Plateosaurus*	Trossingen	R	Artec EVA Surface Scanner	44.6
SMNS 91306^x^ (F48)	*Plateosaurus*	Trossingen	L	Artec EVA Surface Scanner	45.4
SMNS 91310 (F65d512)	*Plateosaurus*	Trossingen	R	Artec EVA Surface Scanner	35.1
GPIT 2^x^	*Plateosaurus*	Trossingen	L	CT-scan from Mallison (2010a)	35
SMNS 80664	*Plateosaurus*	Stuttgart-Degerloch	L	Artec EVA Surface Scanner	55.5
SMNS 12684^x^	*Efraasia*	Pfaffenhofen	R	Artec EVA Surface Scanner	24.4
SMNS 17928	*Efraasia*	Ochsenbach	R	Artec EVA Surface Scanner	32.1
**Radius**					
SMNS 12949	*Plateosaurus*	Trossingen	L	Artec EVA Surface Scanner	22.7
SMNS 12950	*Plateosaurus*	Trossingen	R	Artec EVA Surface Scanner	23.3
SMNS 13200^x^	*Plateosaurus*	Trossingen	L	Artec EVA Surface Scanner	24
SMNS 81914 (F8)	*Plateosaurus*	Trossingen	L	Artec EVA Surface Scanner	27.8
SMNS 91296 (F10) #5	*Plateosaurus*	Trossingen	R	Artec EVA Surface Scanner	18.5
SMNS 91296 (F10) #6	*Plateosaurus*	Trossingen	L	Artec EVA Surface Scanner	23.5
SMNS 91296 (F10) #7	*Plateosaurus*	Trossingen	L	Artec EVA Surface Scanner	23.8
SMNS 91310 (F65)	*Plateosaurus*	Trossingen	R	Artec EVA Surface Scanner	22
GPIT 2^x^	*Plateosaurus*	Trossingen	L	CT-scan from Mallison (2010a)	21.1
SMNS 12354b^x^	*Efraasia*	Pfaffenhofen	L	Artec EVA Surface Scanner	16.4
MB.R.4718.59^x^	*Ruehleia*	Römhild	R	Photogrammetry	26.1
**Ulna**					
SMNS 12949	*Plateosaurus*	Trossingen	L	Artec EVA Surface Scanner	26.6
SMNS12950	*Plateosaurus*	Trossingen	R	Artec EVA Surface Scanner	26
SMNS13200^x^	*Plateosaurus*	Trossingen	R	Artec EVA Surface Scanner	26.3
SMNS 91296 (F10) #8	*Plateosaurus*	Trossingen	L	Artec EVA Surface Scanner	21.2
SMNS 91296 (F10) #9	*Plateosaurus*	Trossingen	L	Artec EVA Surface Scanner	24.3
SMNS 91296 (F10) #10	*Plateosaurus*	Trossingen	R	Artec EVA Surface Scanner	27
SMNS 91306^x^ (F48)	*Plateosaurus*	Trossingen	L	Artec EVA Surface Scanner	28.5
GPIT 2^x^	*Plateosaurus*	Trossingen	L	CT-scan from Mallison (2010a)	23.7
GPIT uncat. #1	*Plateosaurus*	Pfrondorf	R	Artec EVA Surface Scanner	23.6
SMNS 12354b^x^	*Efraasia*	Pfaffenhofen	L	Artec EVA Surface Scanner	18
SMNS 12684^x^	*Efraasia*	Pfaffenhofen	L	Artec EVA Surface Scanner	17.5
MB.R.4718.58^x^	*Ruehleia*	Römhild	R	Photogrammetry	29.7
**Femur**					
SMNS 13200^x^	*Plateosaurus*	Trossingen	R	Artec EVA Surface Scanner	65.8
SMNS 91296 (F10) #11	*Plateosaurus*	Trossingen	R	Artec EVA Surface Scanner	61.8
SMNS 91306^x^ (F48)	*Plateosaurus*	Trossingen	R	Artec EVA Surface Scanner	76.1
GPIT 1^x^	*Plateosaurus*	Trossingen	L	CT-scan from Mallison (2010a)	59.6
GPIT 1^x^	*Plateosaurus*	Trossingen	R	CT-scan from Mallison (2010a)	57.2
SMNS 53537	*Plateosaurus*	Stuttgart-Degerloch	R	Artec EVA Surface Scanner	63.6
SMNS 12220	“*Sellosaurus*”	Pfaffenhofen	L	Artec EVA Surface Scanner	45.2
SMNS 12684	*Efraasia*	Pfaffenhofen	R	Artec EVA Surface Scanner	36.2
MB.R.4718.98^x^	*Ruehleia*	Römhild	L	Photogrammetry	79.2
MB.R.4753	*Ruehleia*	Römhild	R	Photogrammetry	81
**Tibia**					
SMNS13200^x^	*Plateosaurus*	Trossingen	L	Artec EVA Surface Scanner	55.5
SMNS 13200^x^	*Plateosaurus*	Trossingen	R	Artec EVA Surface Scanner	54.4
SMNS 91296 (F10) #12	*Plateosaurus*	Trossingen	R	Artec EVA Surface Scanner	52
SMNS 91296 (F10) #13	*Plateosaurus*	Trossingen	R	Artec EVA Surface Scanner	58.9
SMNS 91306^x^ (F48)	*Plateosaurus*	Trossingen	L	Artec EVA Surface Scanner	63.2
SMNS 91310 (F65)	*Plateosaurus*	Trossingen	R	Artec EVA Surface Scanner	50.9
GPIT 1^x^	*Plateosaurus*	Trossingen	L	CT-scan from Mallison (2010a)	47.8
GPIT 1^x^	*Plateosaurus*	Trossingen	R	CT-scan from Mallison (2010a)	49.3
GPIT RE 7313	*Plateosaurus*	Tübingen	L	Artec EVA Surface Scanner	65.3
MB.R.4398.109	*Plateosaurus*	Halberstadt	R	Photogrammetry	55.5
SMNS 6014 (excluded)	*Plateosaurus*	Erlenberg	R	Artec EVA Surface Scanner	52.8
**Fibula**					
SMNS 13200^x^	*Plateosaurus*	Trossingen	L	Artec EVA Surface Scanner	54
SMNS 13200^x^	*Plateosaurus*	Trossingen	R	Artec EVA Surface Scanner	52.2
SMNS13200a+e	*Plateosaurus*	Trossingen	R	Artec EVA Surface Scanner	57.7
SMNS 91296 (F10) #14	*Plateosaurus*	Trossingen	L	Artec EVA Surface Scanner	57.9
SMNS 91296 (F10) #15	*Plateosaurus*	Trossingen	R	Artec EVA Surface Scanner	54.9
SMNS 91296 (F10) #16	*Plateosaurus*	Trossingen	R	Artec EVA Surface Scanner	46.6
SMNS 91297 (F14)	*Plateosaurus*	Trossingen	R	Artec EVA Surface Scanner	51.1
SMNS 91306^x^ (F48)	*Plateosaurus*	Trossingen	L	Artec EVA Surface Scanner	61.1
SMNS 91306^x^ (F48)	*Plateosaurus*	Trossingen	R	Artec EVA Surface Scanner	60.1
GPIT 1^x^	*Plateosaurus*	Trossingen	L	CT-scan from Mallison (2010a)	47.2
GPIT 1^x^	*Plateosaurus*	Trossingen	R	CT-scan from Mallison (2010a)	48.5
GPIT uncat. #2	*Plateosaurus*	Trossingen	L	Artec EVA Surface Scanner	57.8
GPIT uncat. #3	*Plateosaurus*	?	R	Artec EVA Surface Scanner	58.7

**Notes:**

x Indicates which bones are reliably identified as belonging to the same individual.

Institutional abbreviations: GPIT Institute for Geosciences, Eberhard-Karls-Universität Tübingen, Tübingen, Germany; MB.R Museum fur Naturkunde, Berlin, Germany; SMNS Staatliches Museum für Naturkunde, Stuttgart, Germany.

### Bone digitization

Sampled bones were digitized into 3D models in order to perform 3D GM. They were obtained using surface scanning, photogrammetry and CT-scanning. Surface scanning was performed using a Artec EVA surface scanner and the software Artec Studio 12 (Artec 3D, 2018). The models of specimens coming from the Museum fur Naturkunde were provided by Marco Marzola (using photogrammetry via the software Agisoft Photoscan) (Agisoft LLC, 2018), following the protocol of Mallison & Wings (2014). The models of specimens GPIT I and II were provided by Heinrich Mallison, from a previous publication (Mallison, 2010a). 3D models were decimated to 500,000 faces when they were above this limit, and the left bones were symmetrized on the right side (chosen arbitrarily) for the purposes of the analysis. These two steps were made using Meshlab software (Cignoni et al., 2008). Complete specimens broken in two or more parts were virtually merged using Blender software (Blender Foundation, 2017).

### Landmark acquisition

A dataset of anatomical landmarks was defined for each bone to capture their overall form (Table S1). Because of the scarcity of these anatomical landmarks on limb bones as compared to skulls, and to capture the most accurately the shape of the bones, we chose to use sliding semilandmarks of curves and surface (Gunz & Mitteroecker, 2013). Landmarks and curves sliding semilandmarks were acquired by the same operator on 3D models using the IDAV Landmark software (Wiley et al., 2005). For each bone, a repeatability test was performed by digitizing the anatomical landmark set on three specimens, for ten times each. A Generalized Procrustes Analysis followed by a Principal Component Analysis (see “3D Geometric Morphometrics”) were run to verify that the intra-individual variability (error of measurement) was lower than inter-individual variability (morphological variation). The results (see Fig. S1) showed the expected lower intra-individual signal compared to the inter-individual one. Surface semilandmarks were warped onto 3D models using a template, that is, a model chosen among the sample for which all the landmarks and semilandmarks were placed, for each type of bone, using the “placePatch” function in the Morpho package (Schlager, 2017) in R 3.5.1 (R Core Team, 2018). The surface sliding semilandmarks were manually placed on this template using IDAV Landmark. Curve and surface semilandmarks were slid following the protocol in Gunz, Mitteroecker & Bookstein (2005), minimizing the bending energy of a Thin-Plate Spline first between each specimen and the template (“relaxLM” function in Morpho R package, this step was iterated 5 times), then between the result of the previous step and the Procrustes consensus of the dataset (“slider3d” function in Morpho R package, also iterated 5 times).

### 3D geometric morphometrics

The superimposition of the dataset was done using a Generalized Procrustes Analysis (GPA; see Gower, 1975 and Rohlf & Slice, 1990), removing size, relative position and orientation of the specimens. It was followed by a Principal Component Analysis (PCA), which permits plotting of the superimposed Procrustes residuals in shape tangent space, using geomorph (Adams, Collyer & Kaliontzopoulou, 2018) and Morpho R packages (“gpagen” and “plotTangentSpace” functions in geomorph R package; “procSym” function in Morpho R package). This analysis ordinates the global variation on new axes, the Principal Components (PC). The ordination is performed in order to maximize the explained variation in a reduced number of orthogonal PCs, separating the multivariate initial variability into a new set of uncorrelated axes.

### Visualization and interpretation protocol

Our study was articulated around two main goals: (1) Assess the impact of taphonomy on the variation occurring in the sample; and (2) describe the range of variation attributable to the biological history of the specimens. It was thus necessary to adopt an exploratory approach on our dataset in order to tackle our two-fold question.

To do so, we explored the first 90% of the total variance of each analysis. On each PC, we observed the shape variation using Thin-Plates Splines (TPS) analysis, performed with two different functions and providing two different visualizations. First, with the “pcaplot3d” function (in Morpho) that permits to represent by vectors the displacement of each (semi)landmarks along one PC (also known as “lollipop vectors” (Klingenberg, 2013)); second, with the “plotTangentSpace” (in geomorph) which allows display of the extreme landmark conformations of a PC, facilitating the comparison of shape changes along the PC. These landmark extreme conformations were exported as 3D models using the “vcgPlywrite” function from the Rvcg R package (Schlager, 2017). Each visualized PC was categorized according to the apparent impact of taphonomic deformations affecting the anatomical features of the bone. We have evaluated the degree of taphonomic influence occurring in each PC by seeking the anatomically aberrant variation, so that the discussion of the biologically plausible variation was only based on the most biologically plausible PCs. The categorization of each uncorrelated PC given its degree of biological reliability is based on the recognition of deformation patterns suggesting that a taphonomic process was at the origin of it. The most common taphonomic events described in the literature is compression leading to a taphonomic flattening of the bone (e.g., Baert, Burns & Currie, 2014; Müller et al., 2018), inducing a strong variation of a bone in one axis (usually the mediolateral or the anteroposterior one). Taphonomic bendings (e.g., Wahl, 2009; Fanti, Currie & Burns, 2015) and modifications of original torsions (e.g., Nicholls & Russell, 1985; Remes, 2008), altering respectively the original curvature of the shaft and orientation of the ends of a bone, are also encountered in the literature. The original bone shape and/or volume can be also globally or locally altered by the conditions of fossilization, that is, by cementation with calcite and hematite (Holz & Schulz, 1998) or by the precipitation and swelling of clays (Moser, 2003). In addition to this non-exhaustive listing of plastic deformations, some variation of shape related to minor incompleteness of the bones can also be found. Following the degree of alteration, fossils can display complex mixtures of these variations, increasing the diversity of observable patterns. Furthermore, the PC scores of the left and right bones of a single individual on one PC can give additional support to the categorization of a PC. As the biological asymmetry between the left and right bones should be slight (Hedrick et al., 2019), a relatively high distance between such objects observed in a PC would support a taphonomic interpretation. A Neighbor-Joining clustering analysis (NJ; Saitou & Nei, 1987) was performed for each PCA on the subset of PCs representing the estimated biologically plausible variation, permitting an overall representation of the morphological distance between each specimen by the means of an unrooted tree. NJ trees were calculated using the ape R package (Paradis & Schliep, 2018).

For each analysis, the interpretation of the results based on the protocol detailed here permitted us to categorize each feature varying along each uncorrelated PC into three categories: “obviously taphonomically influenced”, “ambiguous”, and “biologically plausible” (synthesized from the results in the discussion and the Appendix S1). The PCs containing at least one “obviously taphonomically influenced” feature were considered as clearly taphonomically influenced, while the other PCs (containing no “obviously taphonomically influenced” feature) were considered as the most biologically plausible PCs. The investigation of the range of biological variation occurring in our sample was done on the latter category of PCs, because they are uncorrelated to the clearly taphonomically influenced variation of the PCs presenting at least one “obviously taphonomically influenced” variation. The highlighted features were discussed and sorted given their biological reliability, assessed thanks to a corroborative observation of the real sampled specimens. After this step, the distribution of the highlighted features among the sampled specimens are compared to the NJ analyses. This approach permits evaluation of the biological importance of a highlighted compelling feature, or can bring additional support to the biological origin of a less compelling feature (see “Discussion”).

### Testing the effect of size

The allometry, the influence of size variation on morphological variation, is an important factor to study in order to understand the biology of the organisms (see Klingenberg, 2016). In our study, size variation can result not only from allometry, but also from taphonomy (notably influencing, by the processes cited previously, the original shape and size of the bone). It is thus important to try to dissociate the size variation induced by biology from the size variation induced by taphonomy. We performed for each dataset a Procrustes ANOVA (function “procD.allometry” in geomorph; see Goodall, 1991) testing the influence of size (here represented by the natural logarithm of the centroid size) on aligned landmark conformations (see Table S2). This analysis tests the influence of size on the overall variation, including the taphonomically impacted PCs. We also tested the correlation between the natural logarithm of the centroid size on each examined PC for each analysis (Pearson’s correlation test; see Table S2). This complementary test investigates the impact of size regarding the estimated taphonomic influence/biological reliability of each PC.

## Results

The results are here described with terminology mostly following Remes (2008) for the forelimb elements and Langer (2003) for the hindlimb ones.

### Humerus

The first six PCs (91.94% of total variance) are here investigated.

**Figure 1:**
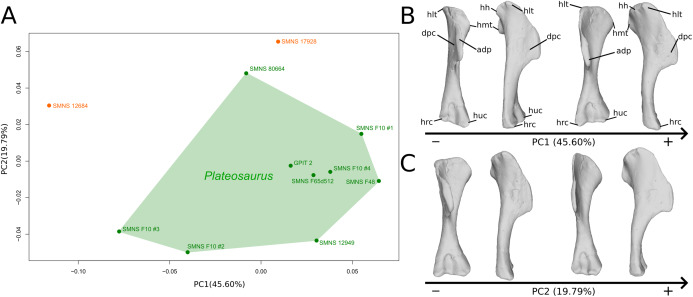
Results of the PCA on the PC1 and PC2 of the humerus analysis (right side illustrated). On the PCA plot (A), the green cluster represents the morphospace occupied by the genus *Plateosaurus*, the orange dots correspond to the *Efraasia* specimens. Extrema of shape changes along PC1 (B) and PC2 (C) are represented in anterior and lateral views. Abbreviations: adp, apex of deltopectoral crest; dpc, deltopectoral crest; hh, humeral head; hlt, humeral lateral tubercle; hmt, humeral medial tuberosity; hrc, humeral radial condyle; huc, humeral ulnar condyle.

The shape changes in the first PC (Figs. 1A, and 1B; 45.60%) do not separate well the different humeri according to their taxonomic attribution or locality, though the *Efraasia* from Pfaffenhofen is rather isolated at the negative extremity. Two of the four F10 humeri are relatively markedly separated on the negative side. The variation occurs mainly on three morphological features: the deltopectoral crest, and the proximal and distal ends. On the negative side, the proximal end of the humerus presents a slight elevation of the proximal part of the deltopectoral crest (area connected to the lateral tubercle), a compact humeral head presenting a sheared pattern mediolaterally, with the medial tuberosity almost flat. The shaft is flattened anteroposteriorly. The distal end is also flattened, but also strongly twisted toward the lateral side. The strongest variation, however, occurs in the deltopectoral crest, from an anteromedial orientation on the negative side to an anterolateral orientation on the positive side (with the apex slightly more incurved medially).

The second PC (Figs. 1A and 1C; 19.79%) separates Trossingen specimens from the ones from the other localities. On the positive side, the proximal part of the humerus is globally anteroposteriorly flattened. The medial side of the proximal part of the shaft is flattened anteroposteriorly, associated with a strong elevation of the proximal part of the lateral tubercle connecting the deltopectoral crest and an expansion of the medial tuberosity mediodistally. The midshaft is slightly more sigmoid. The distal part of the shaft is slender, with a compact distal end (condyles are closer to each other than on the opposite side of this axis; they do not seem compressed anteroposteriorly). Again, on the deltopectoral crest, we observe a similar, but less intense, variation from a lateral orientation (apex slightly incurved medially; positive side) to a more axial one (apex more strongly incurved medially; negative side). The crest is thicker on the negative side.

The third PC (Figs. S2A and S2B; 10.44%) of the shape changes mainly separates two groups from a central cluster two groups: F48 on the positive extremity, and two of the four F10 humeri on the negative extremity (different from those in PC1). F48 is much more distant from the central cluster than the “two F10” group is. Variation occurs around the apex of the crest: the structure’s location is more (positive side) or less (negative side) close to the midshaft transversal plan, with a slight variation of orientation in the anterior view. The orientation of the deltopectoral crest does not vary with the same intensity as in previous PCs. On the positive side, the apex is closer to the midshaft plane, so that the slope of the end of the deltopectoral crest is the steepest (almost perpendicular). The humeral lateral tubercle is developed proximally, the humeral head is almost flat posteriorly, and the medial tuberosity is less prominent but more expanded mediodistally. The shaft is slightly straighter. The distal end varies in the shape of the condyles and in the distance between them, with mediolaterally expanded condyles and greater intercondylar distance on the positive side. On the negative side, the apex of the deltopectoral crest is far from the midshaft plane, resulting in a softer slope of the end of the deltopectoral crest. The humeral head is more compact as compared to the positive direction of the axis. The shaft is slightly sigmoid. The condyles are more compact and the intercondylar distance is smaller.

The fourth PC (Figs. S2A and S2C; 6.69%) separates on the positive extremity Oschenbach *Efraasia* and one of the four F10 humeri (with a less important positive value) from the others. A proximodistal displacement of the deltopectoral crest is observed along the axis, similar as in PC3 but less intensively. On the positive direction, the lateral side of the deltopectoral crest is flatter, the overall shape of the proximal end is flattened anteroposteriorly, the midshaft is sigmoid (whereas it is straight on the negative side) and the distal part of the shaft and the distal end is more compact, with the lateral and medial sides flattened, and the anterior and posterior sides depressed.

**Figure 2:**
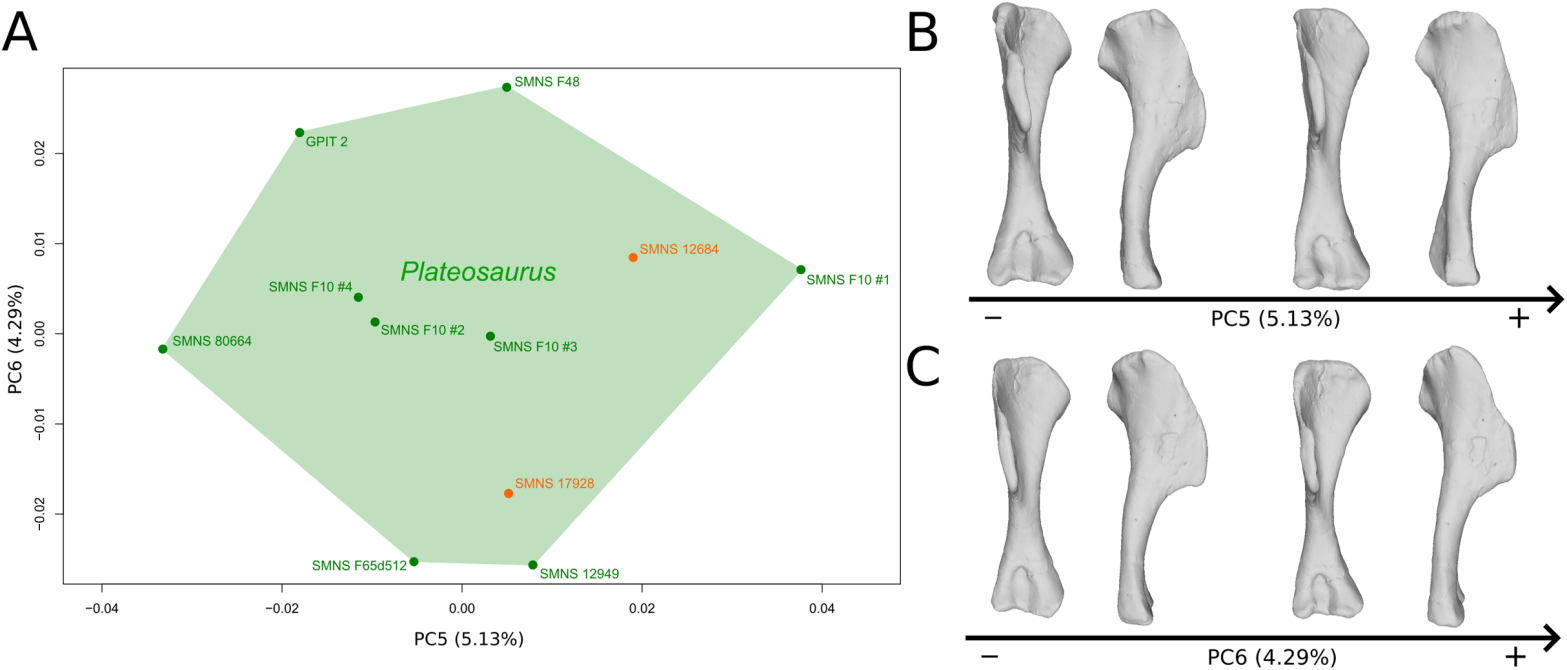
Results of the PCA on the PC5 and PC6 of the humerus analysis (right side illustrated). On the PCA plot (A), the green cluster represents the morphospace occupied by the genus *Plateosaurus*, the orange dots correspond to the *Efraasia* specimens. Extrema of shape changes along PC5 (B) and PC6 (C) are represented in anterior and lateral views.

The fifth PC (Figs. 2A and 2B; 5.13%) does not separate particular clusters. The deltopectoral crest shows slight changes on the apex, which is less expanded on the positive side. The proximal end is flattened, but less intensively than the variations observed in PC4. The main change occurs on the shaft, which displays a sigmoid shape on the negative side and a straight shape on the positive one, as in previous PCs. This variation occurs in the midshaft and also in the proximal part of the shaft. A smooth twist of its distal end occurs, but is substantially less intense than along PC1.

The sixth PC (Figs. 2A and 2C; 4.29%) does not separate particular clusters. The main variation occurs on the deltopectoral crest outline, consisting of variations in slope and apex outline, but also in the presence/absence of an accessory distal process (see “Discussion” part 2.1). On the positive side, the proximal end is more domed with a proximally more developed medial tuberosity, the shaft is more slender and the radial condyle of the distal end is more rounded.

### Radius

The first seven PCs (92.39% of total variance) are here investigated.

**Figure 3:**
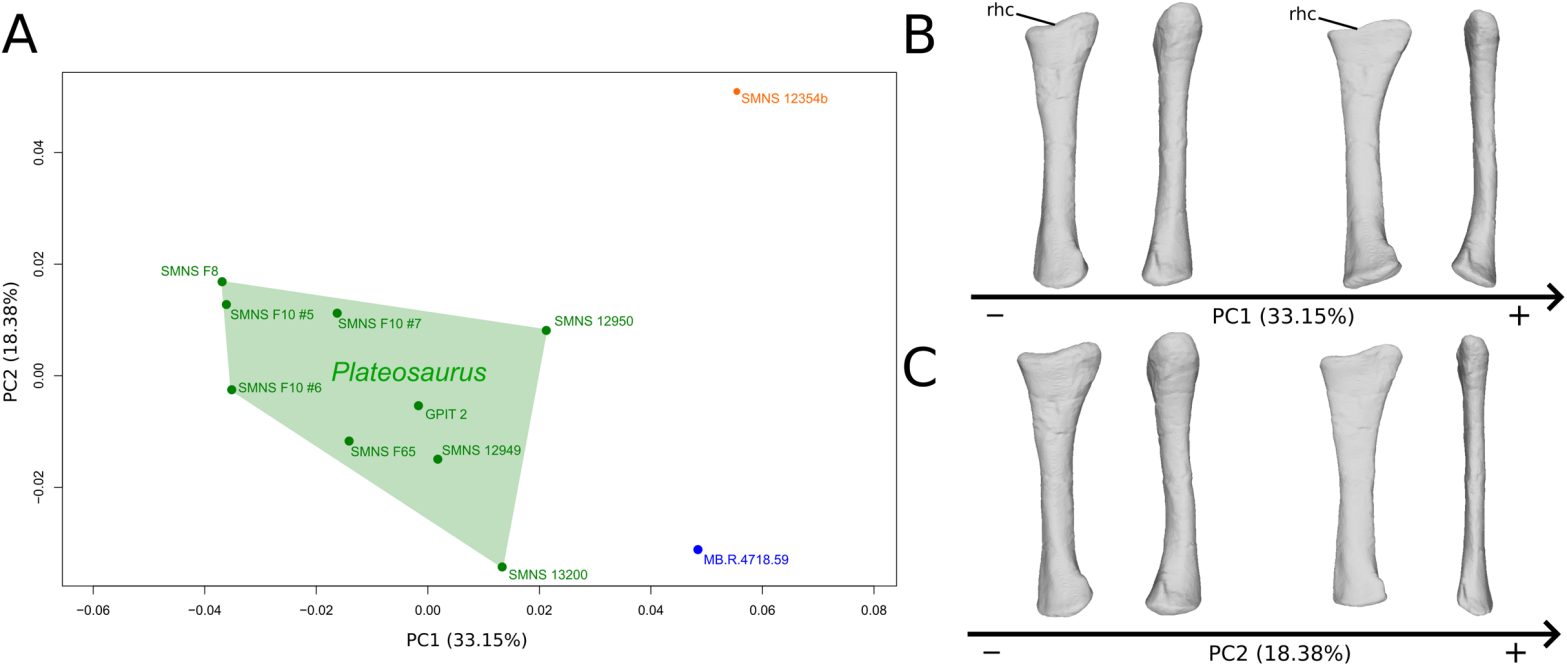
Results of the PCA on the PC1 and PC2 of the radius analysis (right side illustrated). On the PCA plot (A), the green cluster represents the morphospace occupied by the genus *Plateosaurus*, the orange dot corresponds to the *Efraasia* specimen, the blue dot corresponds to the *Ruehleia* specimen. Extrema of shape changes along PC1 (B) and PC2 (C) are represented in medial and posterior views. Abbreviations: rhc, radial humeral cotyle.

The shape changes in the first PC (Figs. 3A and 3B; 33.15%) separate on the positive extremity *Efraasia* and *Ruehleia* from a dispersed *Plateosaurus* cluster. On the positive side, specimens have a mediolaterally flattened proximal half and a more medially curved distal half, giving a wavy shape to the shaft. The distal end outline is less rounded in distal view.

The second PC (Figs. 3A and 3C; 18.38%) separates well on the positive extremity *Efraasia* from a dispersed cluster grouping *Ruehleia* and *Plateosaurus* specimens. On the positive side, the main shape change is a strong mediolateral general flattening.

The third PC (Figs. S3A and S3B; 16.58%) separates SMNS 13200 alone on the positive extremity and a cluster formed by *Ruehleia*, SMNS 12949 and one of the three F10 radii on the negative extremity. On the positive side, the radius shaft tends to be more slender, with a mediolaterally and anteroposteriorly narrower proximal end, and with a deeper humeral cotyle. Also, the distal end is almost square, contrasting with a more robust shaft and an ovoid distal end on the negative side.

The fourth PC (Figs. S3A and S3C; 8.75%) groups on the positive extreme SMNS 12949, SMNS 12950 and one of the three F10 radii, and on the negative extreme F8 alone. On the positive side, the proximal end is slightly more expanded anteroposteriorly, forming a hourglass shape in medial/lateral view. Distally, the shaft is slightly more slender; the distal end is slightly more developed mediolaterally, and slightly more distally oriented.

**Figure 4:**
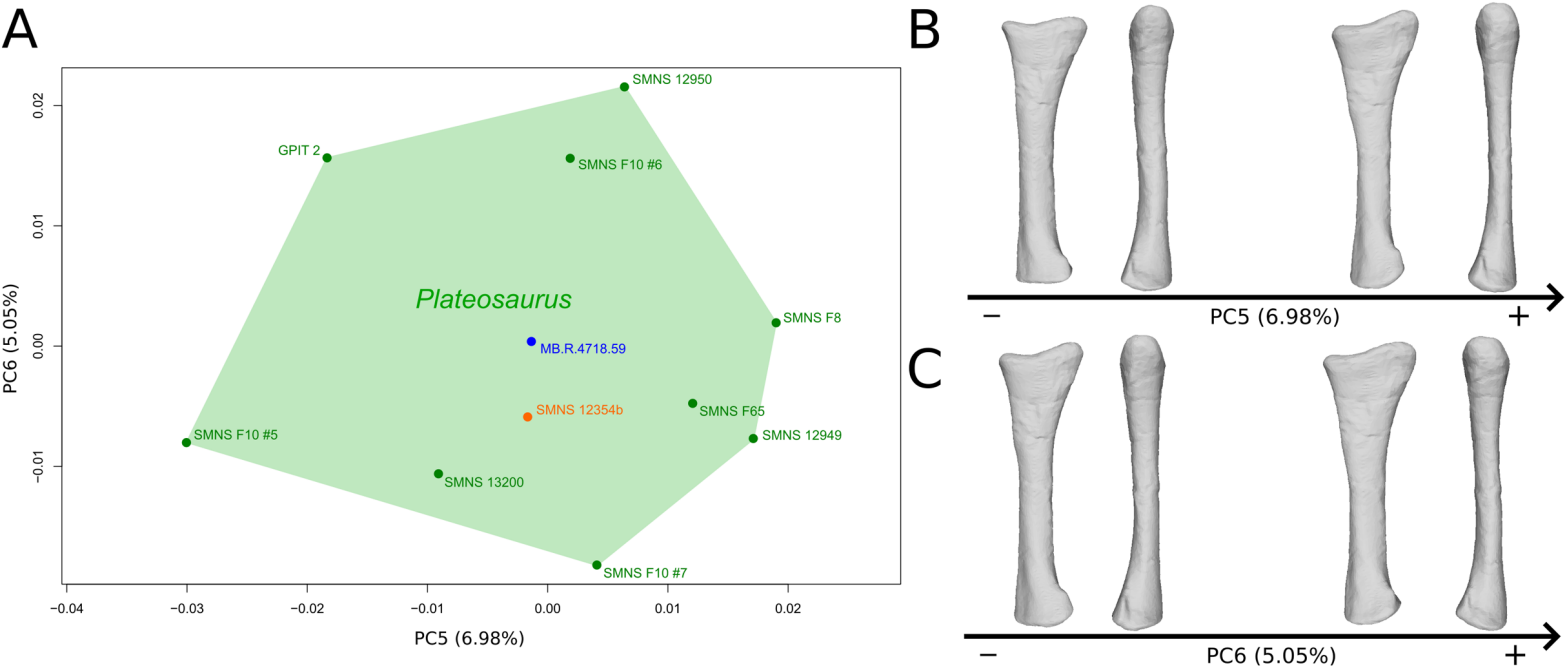
Results of the PCA on the PC5 and PC6 of the radius analysis (right side illustrated). On the PCA plot (A), the green cluster represents the morphospace occupied by the genus *Plateosaurus*, the orange dot corresponds to the *Efraasia* specimen, the blue dot corresponds to the *Ruehleia* specimen. Extrema of shape changes along PC5 (B) and PC6 (C) are represented in medial and posterior views.

The fifth PC (Figs. 4A and 4B; 6.98%) does not separate particular clusters. On the positive side, the shaft is more slender, slightly more curved anteriorly and posteriorly, and the distal end is slightly more inclined medially.

The sixth PC (Figs. 4A and 4C; 5.05%) separates on the positive side SMNS 12950, GPIT2, and one of the F10 radii from the others on the negative side. On the positive side, radii tend to have a slightly more slender shaft, a globally smaller and mediolaterally flatter proximal end and a slightly twisted and mediolaterally flatter distal end.

The seventh PC (Figs. S4A and S4B; 3.50%) tends to separate F65 (negative extremity) from the others (positive extremity). Main shape changes occur on the posterior outline of the proximal and distal ends, more irregular on the negative side, and on the distal part of the shaft, more curved toward the anterior side. Also, the shaft is slightly more (on the positive side of the axis) or less (on the negative side) robust, and the distal half is slightly more anteriorly incurved on the positive side.

### Ulna

The first seven PCs (92.25% of total variance) are here investigated.

**Figure 5:**
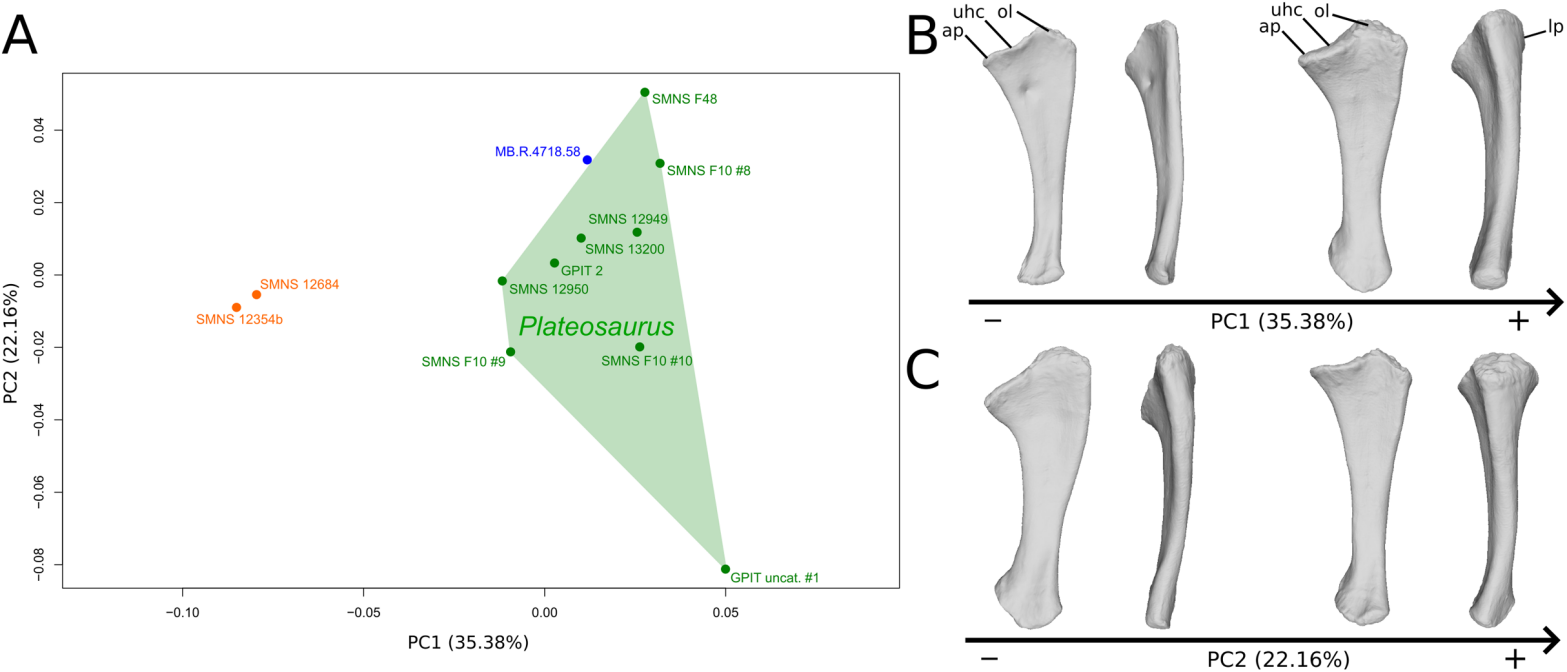
Results of the PCA on the PC1 and PC2 of the ulna analysis (right side illustrated). On the PCA plot (A), the green cluster represents the morphospace occupied by the genus *Plateosaurus*, the orange dots correspond to the *Efraasia* specimens, the blue dot corresponds to the *Ruehleia* specimen. Extrema of shape changes along PC1 (B) and PC2 (C) are represented in medial and posterior views. Abbreviations: ap, anterior process; lp, lateral process; ol, olecranon; uhc, ulnar humeral cotyle.

The shape changes in the first PC (Figs. 5A and 5B; 35.38%) separate the *Efraasia* ulnae (negative extremity) from all the other ones. The deformation on the negative side is an intense mediolateral flattening.

The second PC (Figs. 5A and 5C; 22.16%) separates the ulna from Pfrondorf (negative extremity) from all the other ones. On the negative side the overall shape is flatter mediolaterally. This shape variation is associated with a strongly anteriorly incurved proximal end and an acute and strongly twisted distal end.

The third PC (Figs. S5A and S5B; 12.70%) separates the SMNS 12949 ulna (positive extremity) from the other ones. On the positive side, the overall shape is mediolaterally flattened, with expanded olecranon and anterior processes. The medial side is strongly concave on its proximal half, and the shaft is straight. The distal end is twisted, its anterior part is flattened and expanded laterally.

The fourth PC (Figs. S5A and S5C; 8.55%) does not separate particular clusters. On the positive side, the shaft is more curved mediolaterally, the anterior and lateral processes are more expanded, the anterior part of the distal end is more expanded mediolaterally and the posterior part is slightly shifted.

**Figure 6:**
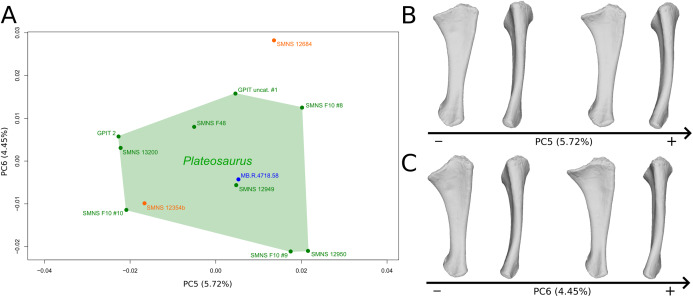
Results of the PCA on the PC5 and PC6 of the ulna analysis (right side illustrated). On the PCA plot (A), the green cluster represents the morphospace occupied by the genus *Plateosaurus*, the orange dots correspond to the *Efraasia* specimens, the blue dot corresponds to the *Ruehleia* specimen. Extrema of shape changes along PC5 (B) and PC6 (C) are represented in medial and posterior views.

The fifth PC (Figs. 6A and 6B; 5.72%) does not separate particular clusters. On the positive side, the shaft is slightly more curved mediolaterally and is very slightly more slender. Also, the lateral margin of the proximal end is more elevated, the margin of the humeral cotyle is sharper, the olecranon is less developed and the distal end is slightly more acute and slightly less developed.

The sixth PC (Figs. 6A and 6C; 4.45%) does not separate particular clusters. The anterior process varies from an anterodistal orientated and expanded shape on the negative side to an anteroproximal orientation and shortened shape on the positive side, whereas the olecranon is less developed. On the negative side, the shaft is slightly more slender, less curved mediolaterally and anteroposteriorly, and the distal end is more developed.

The seventh PC (Figs. S6A and S6B; 3.57%) does not separate particular clusters. The previous anterior process orientation variability is also observed in this PC but only on the apex of the process. On the positive side, the proximal end is larger, the posteromedial margin of the shaft is also slightly sigmoid, and the distal end is globally less developed.

### Femur

The first six PCs (91.40% of total variance) are here investigated.

**Figure 7:**
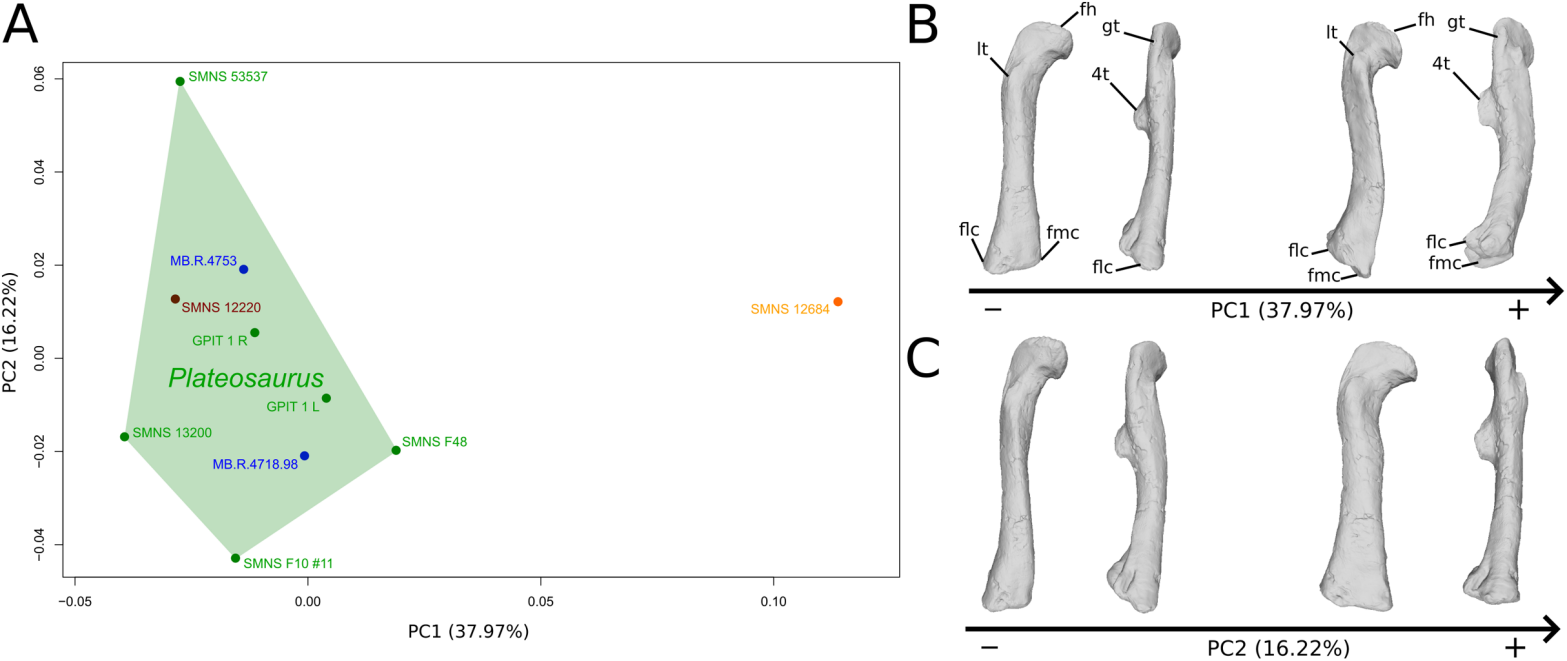
Results of the PCA on the PC1 and PC2 of the femur analysis (right side illustrated). On the PCA plot (A), the green cluster represents the morphospace occupied by the genus *Plateosaurus*, the orange dot corresponds to the *Efraasia* specimens, the blue dots correspond to the *Ruehleia* specimen and the brown dot correspond to SMNS 12220. Extrema of shape changes along PC1 (B) and PC2 (C) are represented in anterior and lateral views. Abbreviations: 4t, fourth trochanter; fh, femoral head; flc, femoral lateral condyle; fmc, femoral medial condyle; gt, greater trochanter; lt, lesser trochanter.

The shape changes in the first PC (Figs. 7A and 7B; 37.97%) separate *Efraasia* (SMNS12684; positive extremity) from all the specimens. The left and right bones of GPIT I are plotting close to each other. The main deformation on the positive side is an overall general mediolateral flattening, affecting all of the shape, notably the femoral head curved toward the shaft, the fourth trochanter and the distal end. The greater and the lesser trochanters are more proximally located, and the distal half of the shaft is strongly curved mediolaterally.

The second PC (Figs. 7A and 7C; 16.22%) tends to separate two specimens on each extreme of the axis: SMNS 53537 on the positive extremity, and F10 on the negative extremity. The left and right bones of GPIT I plot close to each other. On the positive side, the femoral head is more medially oriented and more expanded mediolaterally, whereas the greater and the lesser trochanter are more projected anteriorly. The fourth trochanter is located more closely to the parasagittal plan instead of its classic medial position. The shaft and the distal ends are strongly mediolaterally expanded. The distal end is slightly more laterally oriented.

**Figure 8:**
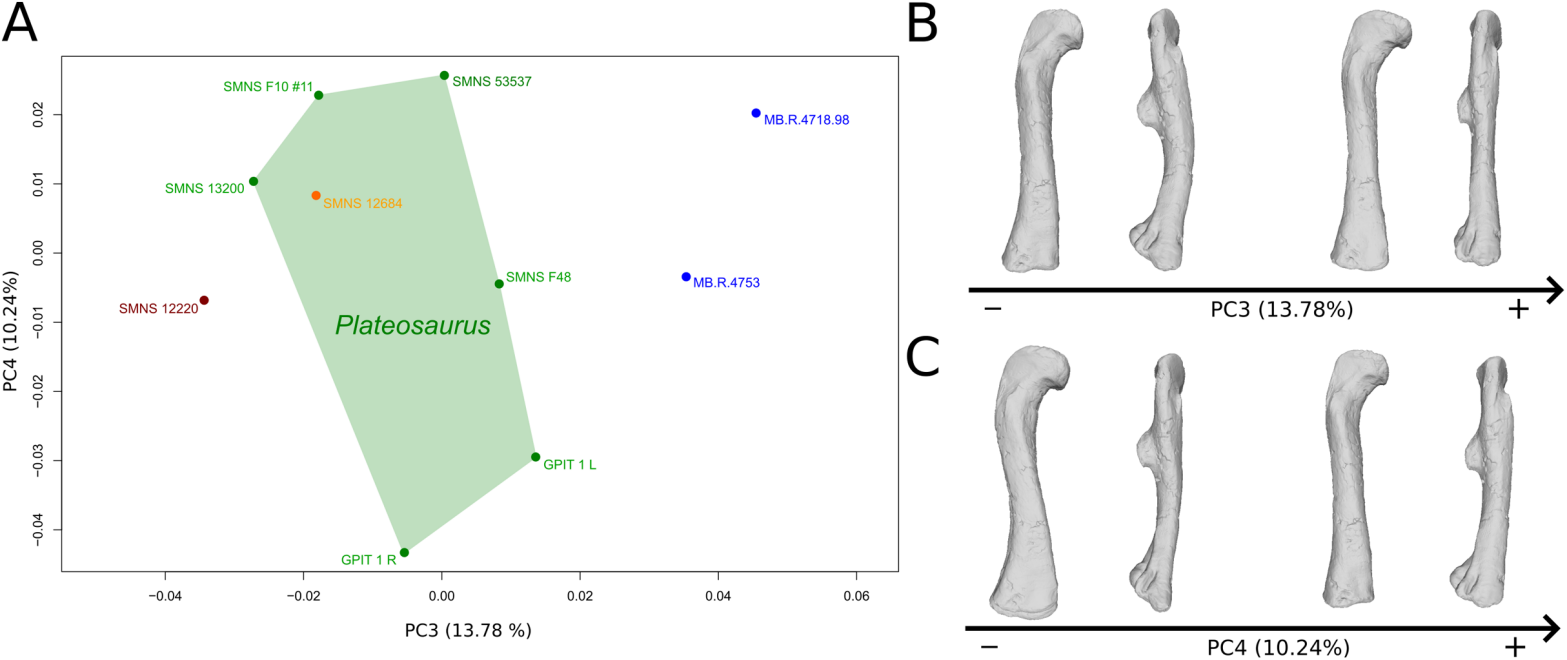
Results of the PCA on the PC3 and PC4 of the femur analysis (right side illustrated). On the PCA plot (A), the green cluster represents the morphospace occupied by the genus *Plateosaurus*, the orange dot corresponds to the *Efraasia* specimens, the blue dots correspond to the *Ruehleia* specimen and the brown dot correspond to SMNS 12220. Extrema of shape changes along PC3 (B) and PC4 (C) are represented in anterior and lateral views.

The third PC (Figs. 8A and 8B; 13.78%) separates *Ruehleia* (positive extremity) femora from the others that form a great dispersed cluster. The left and right bones of GPIT I plot relatively closely to each other. On the positive side, four shape changes occur. The main one is that the shaft is totally straight in medial and lateral views (whereas it is sigmoid on the negative side); the fourth trochanter is slightly more laterally positioned and its outline is slightly sharper; the femoral head is slightly more anteriorly oriented; the distal end is slightly narrower on the anterolateral part, slightly more expanded on the anteromedial part and straight relatively to the shaft.

The fourth PC (Figs. 8A and 8C; 10.24%) separates the two femora (negative extremity) of GPIT I from all the others. The left and right bones of GPIT I plot relatively closely to each other. On the positive side, the femoral head is more compact, the fourth trochanter is straight and more medially positioned, the distal part of the shaft is less curved, and the distal end is more rounded and more distally oriented. On the negative side, the femoral head is markedly more expanded. A strong variation occurs on the fourth trochanter: it is diagonally positioned, from a proximomedial to a more distolateral orientation. The shaft is slightly flatter and less circular. The distal part of the shaft is more laterally oriented, with the anterior ridge extending the lesser trochanter from the proximal to the distal part of the shaft, accentuating the sigmoid curvature of the overall shaft in medial view. The distal part of the shaft is also anteroposteriorly flattened and the distal end is more posteriorly oriented.

The fifth PC (Figs. S7A and S7B; 8.62%) separates on the positive side all of the *Plateosaurus* femora, from a group composed of SMNS 12220, *Efraasia*, and *Ruehleia* specimens on the negative side (we note that the SMNS 12220 and the *Efraasia* specimens are extremely distant from the negative extreme to the origin of the axis; SMNS 12220 is, moreover, very distant from all the other bones). The left and right bones of GPIT I plot closely to each other. On the positive side, the femoral head and the greater trochanter are more developed medially, and the fourth trochanter is more laterally positioned. On the negative side, the femoral head and the greater trochanter are less developed medially, and the fourth trochanter is more medially positioned. The overall distal half is strongly anteroposteriorly flattened, with the condyles of the distal end oriented posteriorly.

The sixth PC (Figs. S7A and S7C; 4.56%) does not separate particular clusters. The left and right femora of GPIT I plot relatively distantly to each other. The femoral head and the shaft are slightly thicker on the positive side than on the negative side. Some slight changes occur on the distal end outline, with a more developed posterior margin of the medial condyle and anterior margin of the lateral condyle on the positive side.

### Tibia

After a first run of the analysis, we excluded SMNS 6014 from the tibia sample, which is an outlier representing a large part of the total variance. Indeed, this specimen has been erroneously restored, so that part of the shaft was missing and the distal end was twisted by 180° (Galton, 2001b), resulting in an anatomically impossible position.

The first six PCs of the analysis without SMNS 6014 (91.73% of total variance) are here investigated.

**Figure 9:**
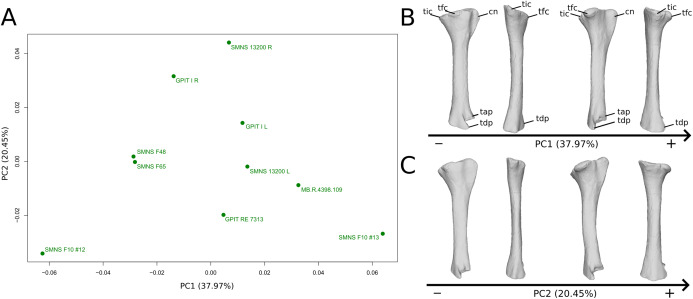
Results of the PCA on the PC1 and PC2 of the tibia analysis (right side illustrated). On the PCA plot (A), the green dots correspond to the specimens of the genus *Plateosaurus*. Extrema of shape changes along PC1 (B) and PC2 (C) are represented in lateral and posterior views. Abbreviations: cn, cnemial crest; tap, tibial ascending process; tdp, tibial descending process; tfc, tibial fibular condyle; tic, tibial internal condyle.

The shape changes in the first PC (Figs. 9A and 9B; 37.97%) separate two extreme specimens from a dispersed central cluster. One of the two F10 tibiae is positioned on the negative extreme, while the other one is on the positive extreme. The left and right bones of GPIT I and SMNS 13200 respectively plot relatively close to each other. On the negative side, the proximal extremity is flattened mediolaterally, with a compact fibular condyle oriented anteriorly and a more distally oriented cnemial crest; the shaft is slightly anteriorly flattened; the distal extremity is twisted anteriorly and distally flattened. On the positive side, the cnemial crest is expanded mediolaterally and anteriorly. The fibular condyle is more oriented posteriorly; the shaft is slightly mediolaterally flattened; the distal extremity is twisted more laterally.

The second PC (Figs. 9A and 9C; 20.45%) does not separate particular clusters. We can therefore note that the left and right tibiae of SMNS 13200 plot distantly relative to each other along this axis: the left tibia is around the origin, whereas the right one is the extreme specimen of the positive side of the axis (this is also the case with both sides of GPIT I, but they plot more closely along the positive side of the axis). On the negative side, the overall shape of the tibia is flattened mediolaterally, notably on the proximal part.

The third PC (Figs. S8A and S8B; 10.76%) separates the left tibia of GPIT I and F65 (negative extremity) from the other ones. The right tibia of GPIT I is, at the opposite, positioned on the positive extremity. The left and right bones of GPIT I plot distantly from each other, whereas left and right tibiae of SMNS 13200 plot relatively closely to each other. On the negative side, the proximal end outline is irregular, with a cnemial crest more medially positioned, a more proximally oriented fibular condyle, a more posteriorly extended internal condyle and an almost straight medial side. The posterior part of the shaft is more expanded. This is also the case of the distal end in anteroposterior directions.

The fourth PC (Figs. S8A and S8C; 7.49%) does not separate particular clusters. The left and right bones of GPIT I plot relatively closely to each other, whereas left and right tibiae of SMNS 13200 plot extremely closely to each other. On the positive side, the outline of proximal end is irregular, with a cnemial crest more developed, and a more expanded fibular condyle and posterior margin. The shaft is slightly anteriorly twisted. The distal end (distal part of the shaft and end) is slightly more twisted, with ascending and descending processes more anteriorly oriented.

**Figure 10:**
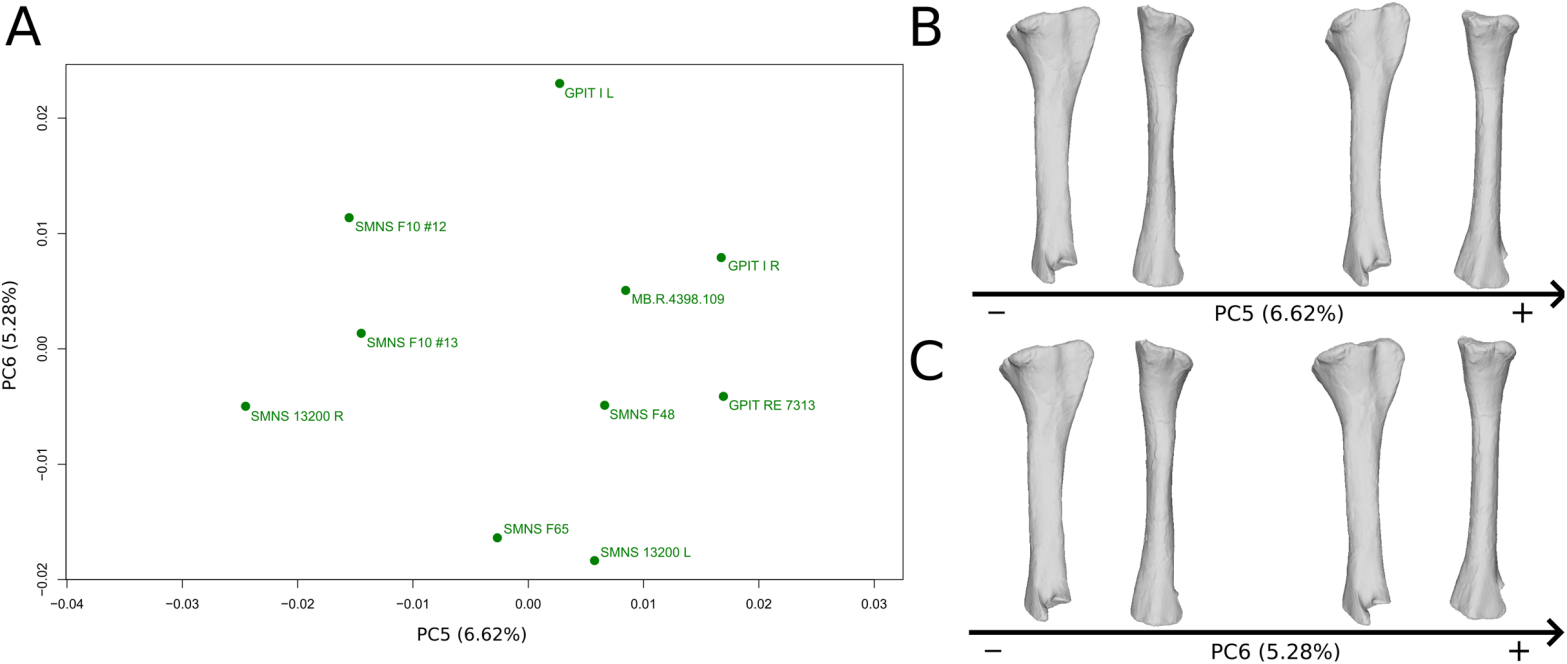
Results of the PCA on the PC5 and PC6 of the tibia analysis (right side illustrated). On the PCA plot (A), the green dots correspond to the specimens of the genus *Plateosaurus*. Extrema of shape changes along PC5 (B) and PC6 (C) are represented in lateral and posterior views.

The fifth PC (Figs. 10A and 10B; 6.62%) separates on the negative side the right tibia of SMNS 13200 and the two tibiae from F10. The two tibiae of SMNS 13200 are far from each other, whereas those of GPIT I are plotting relatively closely. On the positive side, on the proximal end, the cnemial crest is slightly more developed anteriorly, and the internal condyle is more developed posteriorly. The proximal part is slightly more anteriorly oriented (whereas it is more anteroproximally oriented on the negative side). The shaft is medially more circular and more posteriorly expanded. On the distal end, the medial corner is slightly more developed anteriorly, and the posterior margin is more smoothly curved, whereas it is steeper on the negative side.

The sixth PC (Figs. 10A and 10C; 5.28%) does not separate particular clusters. The left and right tibiae of GPIT I and SMNS 13200, respectively, plot closely to each other. On the positive side, the proximal end is flattened mediolaterally. The cnemial crest is slightly more developed. The proximal surface of the end is flattened (whereas it is more developed on the negative side). A portion of the shaft is slightly shifted posteriorly, and the anterior part of the distal surface is more domed.

### Fibula

The first seven PCs (90.79% of total variance) are here investigated.

**Figure 11:**
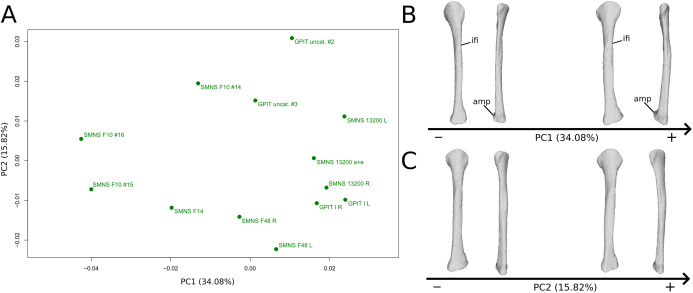
Results of the PCA on the PC1 and PC2 of the fibula analysis (right side illustrated). On the PCA plot (A), the green dots correspond to the specimens of the genus *Plateosaurus*. Extrema of shape changes along PC1 (B) and PC2 (C) are represented in lateral and posterior views. Abbreviations: amp, anteromedial projection (following Otero et al., 2015); ifi, iliofibularis insertion.

The first PC (Figs. 11A and 11B; 34.08%) separates on the negative side a dispersed group formed by the three F10 and F14 fibulae from the others (on the positive side, less dispersed). The bones belonging to the same specimen (GPIT I, SMNS 13200, F48) are close to each other on the positive side. Major shape changes occur on the shaft, ranging from a slightly medially curved (negative side) to a strongly laterally curved shaft (slightly posteriorly deviated; positive side). On the negative side, the medial and lateral margins of the proximal end are more curved laterally, with the anterior part slightly less developed; the anterior and posterior extremes of the distal end are more flattened and expanded proximally.

The second PC (Figs. 11A and 11C; 15.82%) does not separate particular clusters. The bones belonging to the same specimen plot relatively closely to each other. On the positive side, the shaft is slightly flatter and more expanded anteriorly. The proximal end is tighter mediolaterally and more expanded anteroposteriorly; the proximal half is slightly more twisted laterally. On the distal end, the anterolateral and posterior margins are slightly less developed, whereas the anteromedial projection (craniomedial projection in Otero et al., 2015) is more prominent proximally.

The third PC (Figs. S9A and S9B; 13.48%) separates two fibulae on the positive (one of the three F10 and F14) and negative (another F10 and one of the two SMNS 13200) extremities from a central cluster. The bones belonging to the same individuals plot relatively close to each other (except for SMNS 13200 fibulae which are more distant). On the negative side, the proximal end is strongly flattened anteriorly and more expanded mediolaterally; the proximal part of the shaft is pinched, whereas the midshaft is laterally expanded. The distal end is more developed, with a relatively strong twist of the anterior half.

The fourth PC (Figs. S9A and S9C; 9.63%) separates three fibulae (SMNS 13200a+e, one of the three F10 and the right fibula of GPIT I) on the positive side from a more closely grouped negative cluster. The bones belonging to the same individuals plot relatively close to each other. On the positive side, the proximal end is strongly flattened anteriorly and slightly more developed posteriorly. The shaft is slightly more developed anteriorly and slightly less so posteriorly. The distal end is slightly more developed anteriorly and slightly less so posteriorly.

**Figure 12:**
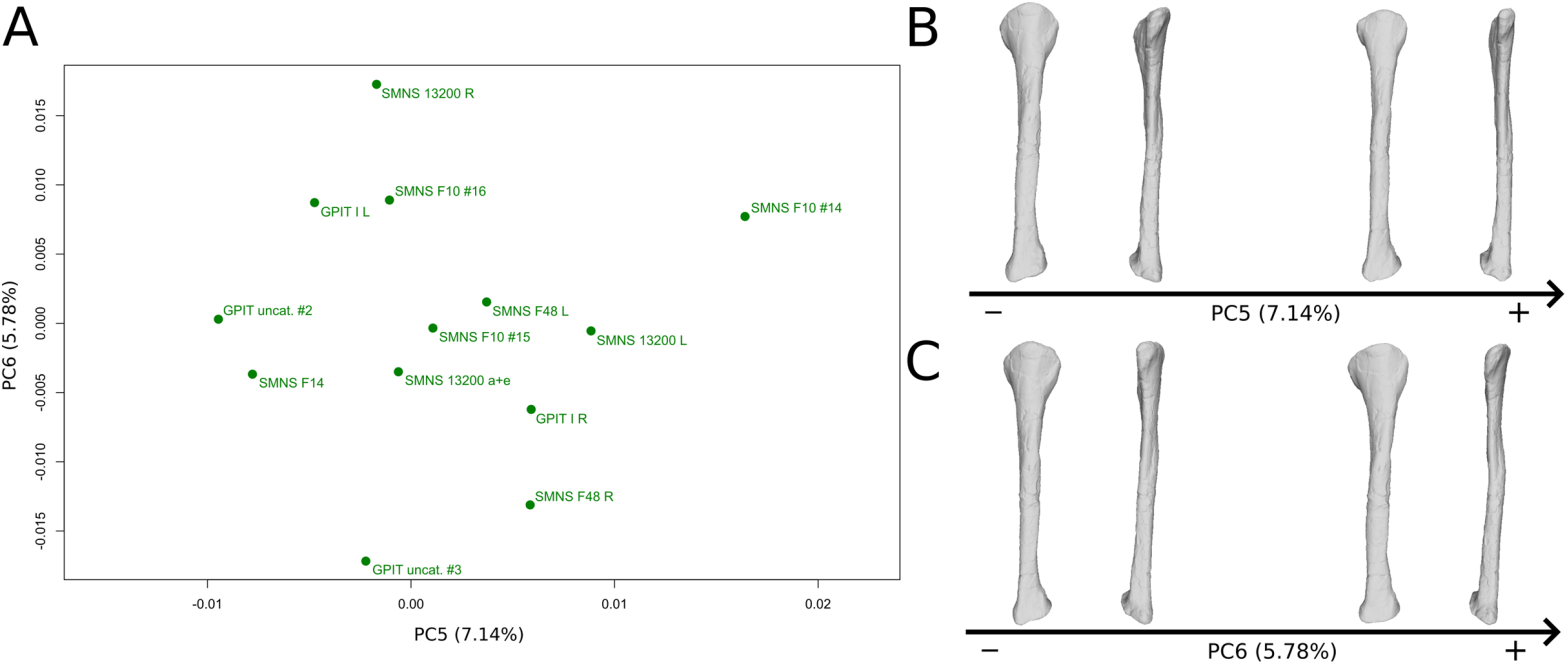
Results of the PCA on the PC5 and PC6 of the fibula analysis (right side illustrated). On the PCA plot (A), the green dots correspond to the specimens of the genus *Plateosaurus*. Extrema of shape changes along PC5 (B) and PC6 (C) are represented in lateral and posterior views.

The fifth PC (Figs. 12A and 12B; 7.14%) does not separate particular clusters, although one of the F10 fibulae is distant from the others, on the positive side. The bones belonging to the same individuals plot relatively distantly to each other, except for F48. On the positive side, the overall shape is mediolaterally flattened with the exception of the distal half of the shaft, which is more curved laterally. The proximal and distal ends are globally less developed.

The sixth PC (Figs. 12A and 12C; 5.78%) does not separate particular clusters. The bones belonging to the same individuals plot slightly distantly to each other. On the positive side, the proximal end is slightly twisted proximodistally on the lateral view, and the anterior part is slightly less developed. The anterior half of the shaft is more expanded anteriorly and less so posteriorly, whereas all of the distal part of the shaft is more developed. The distal end is slightly twisted distally in lateral view and mediolaterally in distal view. The anteromedial projection is also slightly more developed.

The seventh PC (Figs. S10A and S10B; 4.85%) does not separate particular clusters. The bones belonging to the same individuals plot relatively distantly to each other, except for F48. On the positive side, the anterior part of the proximal end is slightly incurved laterally and slightly less developed laterally. A slightly larger region forming a depression around the midshaft, probably corresponding to the iliofibularis insertion (Langer, 2003; McPhee et al., 2014), is more proximally positioned. The outline of the distal end is less developed.

### Test of the impact of size

Considering the results of the Procrustes ANOVA performed for each dataset, only the aligned landmark conformations of the ulnae were significantly (*p* < 0.05) correlated to the logarithm of the centroid size (see Table S2). Considering the correlation tests performed on each PCs, 4 of the 39 PCs herein investigated were significantly correlated with size: PC1 of humerus, PC1 of ulna, PC3 of femur, PC 7 of fibula.

## Discussion

Every features varying along each uncorrelated PC were classified into three categories: “obviously taphonomically influenced”, “ambiguous”, and “biologically plausible” (see the Appendix S1 part I, referencing every interpretation for every described feature).

### Taphonomic influence on morphological variation

We categorized the PCs 1–3 for the humerus, 1–3 for the radius, 1–3 for the ulna, 1–2 and 5 for the femur, 1–4 for the tibia, and 1 and 3–5 for the fibula as clearly taphonomically influenced, because these PCs were presenting at least one “obviously taphonomically influenced” variation. It constitutes most of the total variation explored in our study (75.83% of the total variance for the humerus analysis, 68.11% for the radius, 70.24% for the ulna, 62.81% for the femur, 76.67% for the tibia, and 64.33% for the fibula).

**Figure 13:**
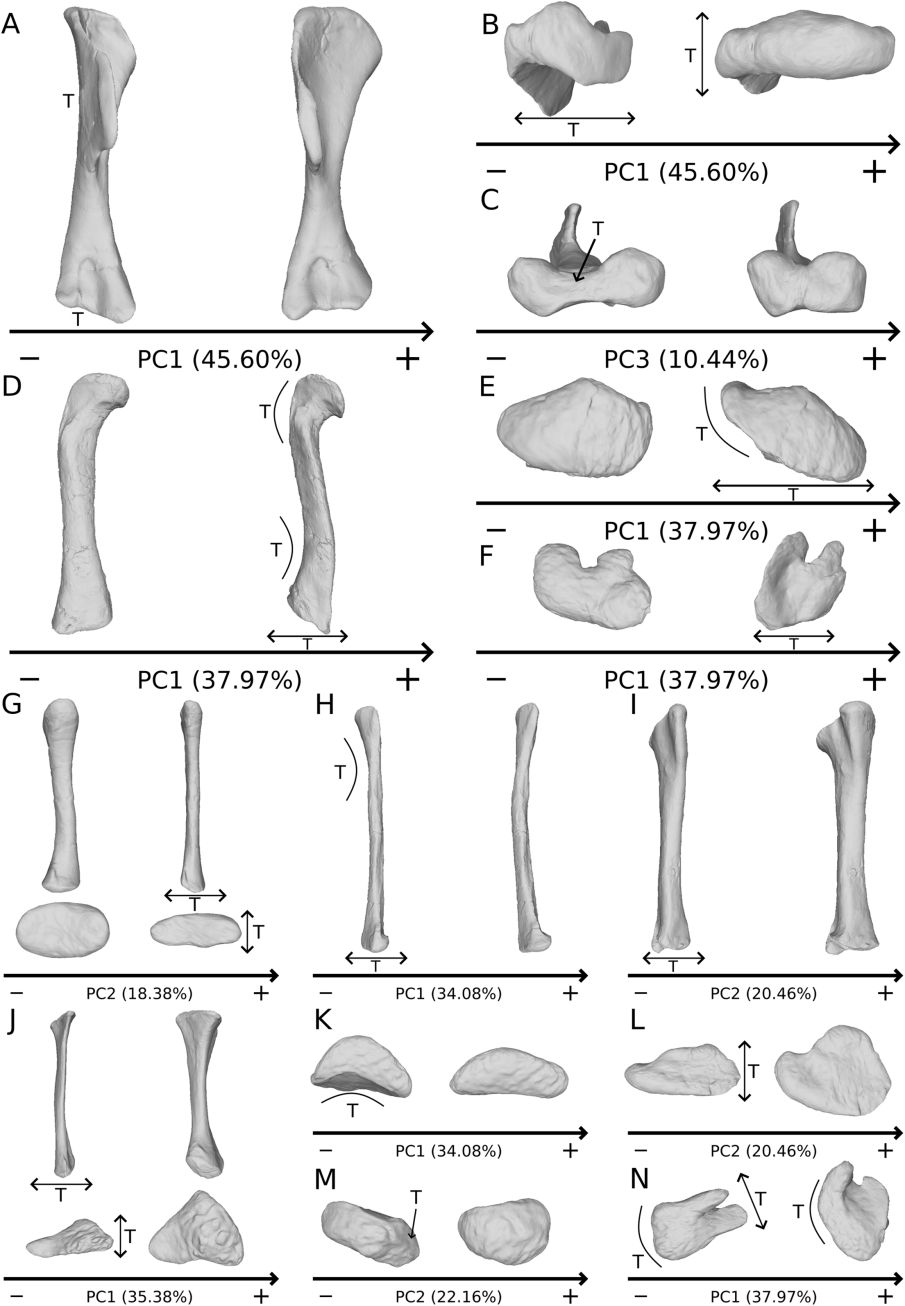
Examples of obviously taphonomically influenced variations not taken into account in the biological interpretations. Observations on theoretical shapes representing variations on the humerus in anterior (A), proximal (B) and distal (C) views; the femur in anterior (D), proximal (E) and distal (F) views; the radius in posterior (top) and proximal (bottom) views (G); the fibula (H) in anterior view; the tibia in anterior view (I); the ulna in anterior (top) and proximal (bottom) views (J); the fibula in proximal view (K); the tibia in proximal view (L); the ulna in distal view (M) and the tibia in distal view (N). The letter T denotes the areas affected by taphonomic deformations. Associated double-headed arrows correspond to a flattening variation, associated curves correspond to a bending or a modification of torsion variation. Associated single headed arrows or absence of associated signs correspond to particular patterns of variation (see text).

The variation depicted on these PCs (Fig. 13), comprising some characters such as the deltopectoral crest variation of orientation on humeri (Fig. 13A) or the general flattening on femora (Figs. 13D–13F), are therefore discarded from the analysis of the biological variation. Among these variations, some of them are redundant, such as the strong general (and most of the time mediolateral) flattening of the bones, occurring as the main variation for the radius, ulna, femur and tibia (Figs. 13D-13J, 13L and 13N). Such a strong general flattening obviously reflects that a taphonomic compression influenced the bone. Strong flattening or expansion variations can also be observed locally in every possible direction (Figs. 13B and 13C). They are observed in all the bones studied, either on the shaft or at the ends. Strong flattening is relatively frequently found in other non-sauropodan sauropodomorphs, affecting for instance the proximal part of the radius and of the ulna of *Unaysaurus tolentinoi* (McPhee et al., 2019), the femur of *Gryponyx africanus* (Haughton, 1924) the tibiae of *Chromogisaurus novasi* (Ezcurra, 2010) and *Lessemsaurus sauropoides* (Pol & Powell, 2007), and most of the appendicular material of *Sarahsaurus aurifontanalis* (Marsh & Rowe, 2018). Strongly flattened bones obviously do not represent the original shape of fossils, implying a distortion in one or more directions. Extreme caution is thus required in these cases because a strong flattening can modify the qualitative appearance of the features of a bone (Müller et al., 2018). Linear measurements are obviously impacted when studying highly flattened bones, and should not be taken in such cases in a biological study, as suggested for proportional characters by Hedrick & Dodson (2013), in the context of using ratios or relative positions as taxonomic/phylogenetic characters. The qualitative characters describing shapes can be also affected, since the original form of the processes on a bone can be altered, as already noted for ilia by Müller et al. (2018). The coding of characters defining only the presence or the absence of a structure should not be affected.

Besides these flattening patterns, strong variations of the curvature of the shaft and of the orientation of the ends are found in all the analyses, reflecting taphonomic bendings and accentuations or attenuations of torsion (Figs. 13A, 13D, 13E, 13H, 13K and 13N). Among those, the variation of orientation of the deltopectoral crest of the humerus (Fig. 13A) seems highly sensible to taphonomic influence, as already pointed out by Remes (2008). As for the variation of bending of the shaft, it can lead to anatomically aberrant morphologies (compared to e.g., Otero (2018)). One of the most obviously influenced example is the femur of *Efraasia* of our sample (SMNS 12684; well represented by the positive side of Fig. 13D), in which the shaft shows a strong sharp lateral deviation, so that the distal end is directed almost laterally. Similarly, the degree of curvature of the bones can be modified by taphonomy, reducing or accentuating the natural bending of bones. Similar statements can be made for the variation of orientation. The reduction or the amplification of torsion (i.e., angle between the long axes of the proximal end versus the distal one) is often discussed in the literature. It has been observed, for example, in the humeri of *Antetonitrus ingenipes* (McPhee et al., 2014), as well as for a collection of femora of the silesaurid *Sacisaurus agudoensis* (Langer & Ferigolo, 2013). This degree of torsion is often discussed as biologically meaningful (e.g., humeral torsion in Bonaparte (1971); Langer, Franca & Gabriel (2007); Remes (2008)). It is thus important to take with caution the biological interpretations that can be made on the variation of this feature among non-sauropodan sauropodomorphs, as it may be altered taphonomically in a substantial number of fossils from this group.

Also, some strong outline and development variations of the ends of the bones obviously reflect some missing part of the bones (Fig. 13M). This observation brings us to consider these variations as biased by taphonomic breaks, abrasion, or preservation variation. Such changes alter superficially the original bony shape of the bones. The preservation variation is assessed here as the cartilaginous part of the ends of the sauropodomorph limb bones, which represent a large missing proportion of the original shape (Holliday et al., 2010; Bonnan et al., 2013), sometimes fossilized at least partially (Schwarz, Wings & Meyer, 2007; Mallison, 2010c; Müller et al., 2017). Consequently, preservational differences of specimens attributed to the same species (Mallison, 2010c; Müller et al., 2017) can lead to morphological variation that may be totally (unpreserved parts in some specimens that are preserved in some others) or partially (biologic variation of ossification) taphonomic. In most of the biological cases, this preservation seems to correspond to the calcified cartilage largely conforming to the osseous subchondral shape (Tsai & Holliday, 2014), so that the variation should be subtle. Such variation could, however, interfere with the search for slight intrageneric variation.

The predominance of the taphonomic signal in our analyses shows that it is paramount to integrate the management of taphonomy in study designs when tackling the biological study of a sample containing fossils. Given our data and previously published studies (Hedrick & Dodson, 2013; Hedrick et al., 2019), it seems important to explicitly discuss the difficulties encountered with taphonomic deformations. In our study, the influence of taphonomy is very important, given that our investigation is focused on low-taxonomic level variations that can be relatively subtle. This impact may even be underestimated relative to the global existing material of limb long bones housed in the visited collections, at least regarding the quality of preservation of the features captured by the anatomical landmarks, because only the bones preserving the complete set of anatomical landmarks used in this study were included in the analyses. The influence of taphonomy should be lower in studies focusing on variations occurring at a higher taxonomic level. In order to minimize the impact of taphonomy on a morphometric analysis, an a priori elimination of the bones showing strong taphonomic deformations can be intended. However, this strategy implies to have a sufficiently substantial sample size allowing discard of specimens without diminishing too drastically the number of specimens, which is often difficult in paleontological studies. Instead, we propose here an a posteriori management of taphonomy, by taking only into account the subset of biologically plausible PCs, uncorrelated to the obviously taphonomically influenced PCs. This approach permits use of quantified results as a tool in order to highlight the most biologically plausible traits of a sample.

### Biologically plausible morphological features

The interpretation of the biologically plausible variation has highlighted the following features:

#### Humerus

**Figure 14:**
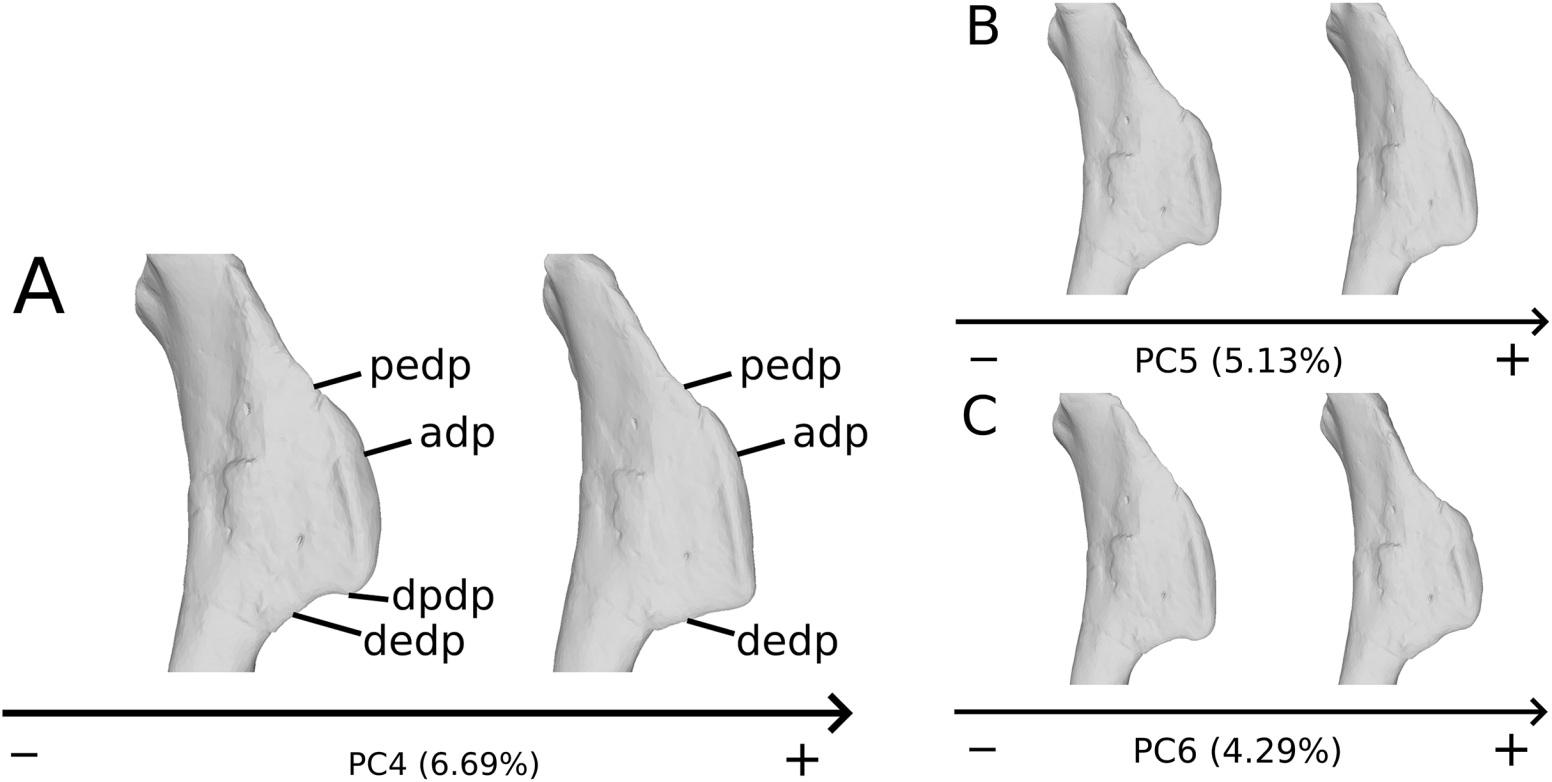
Deltopectoral crest morphological variation on the biologically plausible PCs (i.e., PCs 4.5 & 6). On the PC4 (A), PC5 (B) and PC6 (C), the theoretical shapes corresponding to the minimum of variation along the axis are represented on the left, whereas the shapes corresponding to the maximum of variation are represented on the right. Abbreviations: adp, apex of deltopectoral crest; dedp, distal edge of deltopectoral crest; dpdp, distal process of deltopectoral crest; pedp, proximal edge of deltopectoral crest.

The humerus is one of the most complex limb bones amongst non-sauropodan sauropodomorphs, notably because of the strongly developed deltopectoral crest, whose orientation is highly sensitive to taphonomic influence. The most biologically plausible variation patterns are located on four main spots:

On the deltopectoral crest (Fig. 14), the shape changes of the outline are biologically plausible, as they do not impact the plausible disposition of the muscles in this area (compared to e.g., Otero, 2018), because they do not involve changes of orientation. The whole outline of the deltopectoral crest is variable. We can separate here the crest into three areas, the apex, the proximal edge and the distal edge of the deltopectoral crest. The proximal edge variation occurs mainly in slope steepness. On the apex of the deltopectoral crest, the convexity of the outline varies in lateral view, ranging from a slightly domed curvature to a totally flat outline. In anterior view, the shape of the apex can be straight to curved, generally associated respectively with a tight to large transversal thickness. Also, the relative position of the apex region can slightly vary from a proximal to a more distal position. A variation of steepness of the distal edge of the deltopectoral crest is also observed. A final variation occurs between the last two areas depicted, consisting of the presence or the absence of a small development terminating the apex distally (distal process of deltopectoral crest in Fig. 14), forming a small bump perturbing the linearity of the slope ending the deltopectoral crest. Besides the outline variations, some slight lateral flattening variations also occur, but can hardly be dissociated from orientation and/or general flattening variations that are clearly taphonomic.

**Figure 15:**
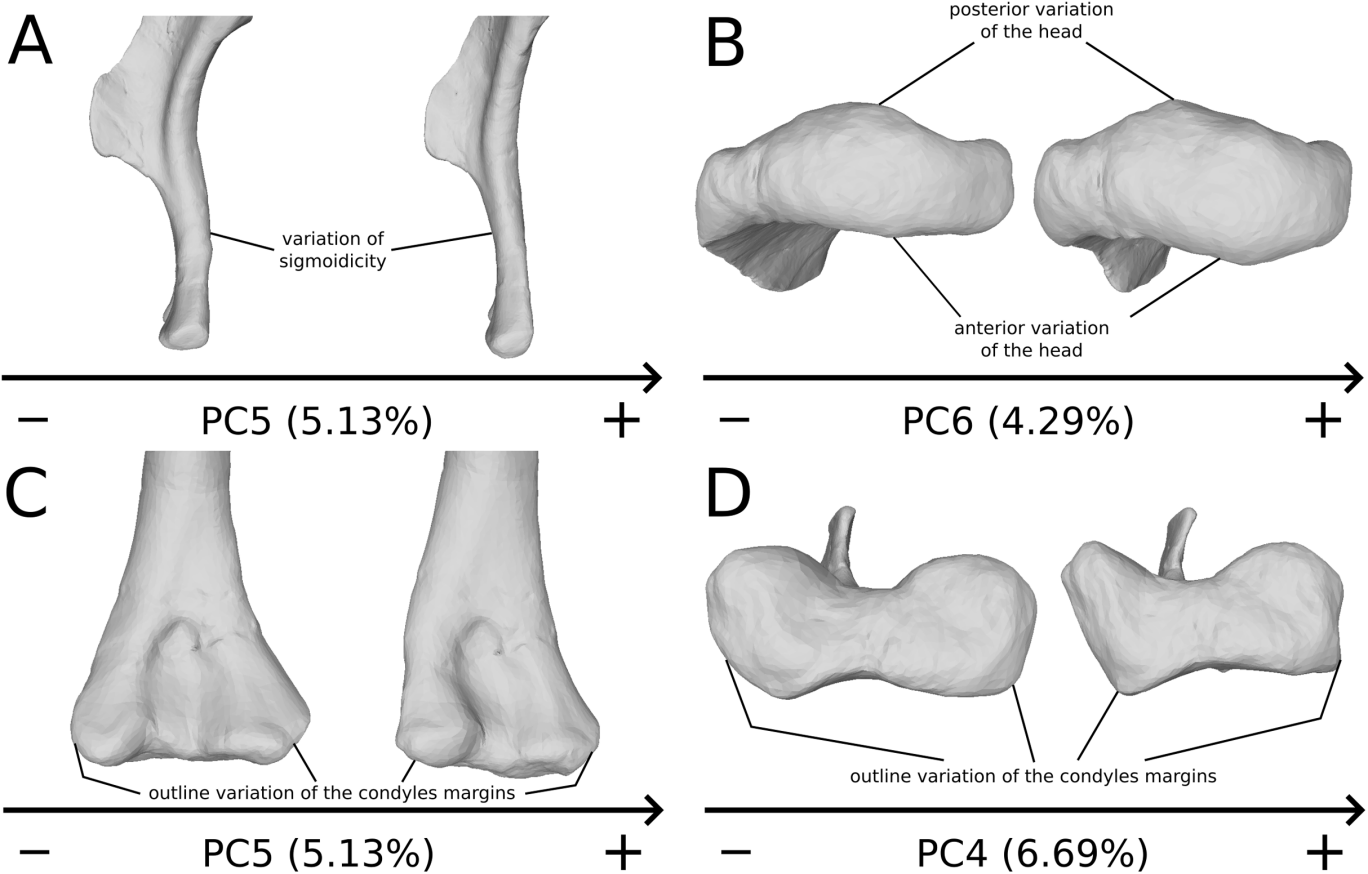
Selected close-ups of biologically plausible humeral variation (i.e*.*, PCs 4, 5 & 6). Variation on PC5, humeral shaft in medial view (A), on PC6, proximal end in proximal view (B), distal end on PC5 in anterior view (C) and on PC4 in distal view (D). The theoretical shapes corresponding to the minimum of variation along the axis are represented on the left, whereas the shapes corresponding to the maximum of variation are represented on the right.

On the shaft (Fig. 15A), shape variation occurs, from straight to sigmoid. Some slight variation of robustness of the shaft also occurs. Because some other genera of non-sauropod sauropodomorphs show sigmoid humeral shafts (Langer, Franca & Gabriel, 2007; Remes, 2008), we consider this variation as biologically plausible. However, such variations can be accentuated by slight taphonomic processes. This assessment is based on specimens of dinosaurs preserving a left and a right humerus of the same individual, but each of them showing a different condition (e.g., holotype of “*Ischisaurus cattoi*” Reig, 1963; R. Lefebvre, 2019, personal observation). Consequently, as biological and taphonomic signal can be here confounded, variations in shaft shape should be interpreted with caution.

On the proximal end (Fig. 15B), principal shape changes are linked to an anteroposterior flattening of the humeral head. The observed variation can be depicted as a quite rounded to flat development of the humeral head. The development of the humeral head can be, however, decoupled between its anterior and posterior margins, varies independently in our analysis (i.e., on different PCs). Although it does not impact the shape of the insertion site of the humerus with the glenoid articulation of the scapulocoracoid, this pattern of variation may also correspond to a taphonomic compression of the humerus. Some caution would be thus necessary before making biological interpretation of this variation (e.g., by inspecting the flattening of the condyles, tubercles and tuberosities, which are supposed to be relatively round).

On the distal end (Figs. 15C and 15D), the most important shape changes occur in the shape of the condyles. They affect the lateral margin of the ulnar condyle and the medial margin of the radial condyle, essentially varying from a rounded to a flat shape with sharp angles. These changes seem somewhat important at this taxonomic scale, notably seen in anterior view (Fig. 15C). Although a part of these changes may be biologic, it is mixed with some obviously taphonomic variation caused by bone modifications (i.e., deformations, breaks, abrasion, or preservation biases) depicted previously. Some slight twist patterns also occur in the analyzed variation, but this signal is also mixed with the obviously taphonomically modified humeral torsion depicted previously. It is therefore necessary to be cautious on the conclusions that we can draw from this area of the humerus.

#### Radius

**Figure 16:**
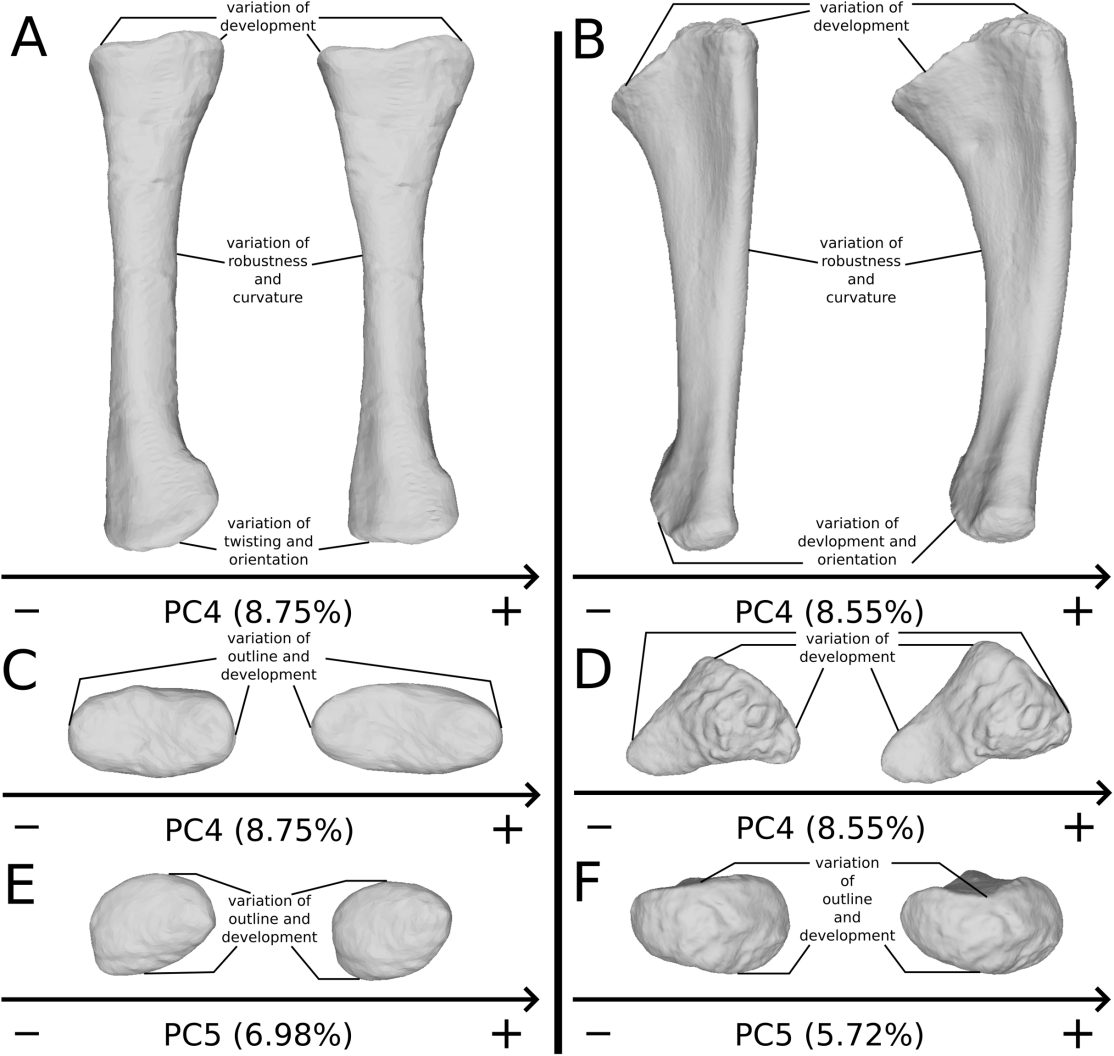
Selected close-ups of biologically plausible radial and ulnar variation (i.e., PCs 4, 5, 6 & 7). For radii, variation on PC4 of radial shaft in medial view (A) and proximal end in proximal view (C), on PC5, distal end in distal view (E); for ulnae, variation on PC4 of ulnar shaft approximatively in medial view (B) and of proximal end in proximal view (D), on PC5, distal end in PC5 (F). The theoretical shapes corresponding to the minimum of variation along the axis are represented on the left, whereas the shapes corresponding to the maximum of variation are represented on the right.

The radius is a relatively simply shaped bone. It is nearly cylindrical with an ovoid proximal end and a subcircular, nearly posteromedially inclined, distal end. Despite strong taphonomic patterns (i.e., mainly general compressions), some more reliable features are highlighted:

On the shaft (Fig. 16A), the main shape change corresponds to a variation of robustness. Some variations also occur on the curvature of the shaft, particularly on the posterior margin. It seems that there is a possible correlation between the slenderness and the accentuation of the curvature of the shaft. Because these variations are not aberrant anatomically, we consider it biologically likely. However, these variations are mixed with the taphonomic variation occurring in the obviously taphonomically influenced PCs. Consequently, we need to be cautious on the interpretations that can be drawn from this morphological feature.

On the proximal end (Figs. 16A and 16C), the main shape change is the variation of development of the end. Slight variations of outline also occur. These variations are likely to be caused by bone modifications, leading to the loss of a part of the proximal processes. The variation of the outline of radial head must therefore be interpreted with caution. Slight variation of inclination of the head may also occur.

On the distal end (Fig. 16E), similar changes of development and outline occur, leading to the same caution in the interpretation of the variations of the distal end. This variation is, indeed, also mixed with variation on obviously taphonomically influenced areas depicted previously. Variations in twisting and orientation also occur (Fig. 16A). Although slight, these variations can be confounded or accentuated by taphonomic deformations, notably bendings and torsions.

#### Ulna

The general shape of the ulna is more complex than the shape of the radius, with a triangular proximal end, an incurved shaft, and a rounded distal end. Removing the strong taphonomic patterns seen in the first PCs, some more biologically plausible morphological features remain in three main regions:

The shaft (Fig. 16B) varies in terms of curvature mediolaterally, particularly on the posterior margin, which seems biologically plausible, because the changes are slight and not anatomically aberrant. These variations might also be caused by a slight mediolateral general taphonomic compression of the bone. However, it would have been expected that such a compression also affects the proximal and distal ends; this is not the case for the slight variation of the most biologically plausible variation studied here. As in the radius, a variation of the robustness of the shaft is observed. It is more pronounced in the anteroposterior plane; this corresponds with the radius, where the variation is observed relatively similarly in the anteroposterior and the mediolateral plan. Because this variation is also mixed with the obviously taphonomically influenced variation, some caution on the biological interpretations that can be drawn here is necessary. Some slight changes of sigmoidicity are also observable.

On the proximal end (Figs. 16B and 16D), shape changes of the anterior process affect its development, but also its proximodistal orientation. The lateral process shows similar patterns of variation of development. This variation is, however, less intense than the variation of development observed for the anterior process. The olecranon shows similar patterns of variation of development, but not necessarily coupled with the variations of development of the anterior and lateral processes. The variation of development of these structures could be caused by biological processes. Indeed, as observed by Mallison (2010c) for the olecranon of the thyreophoran *Kentrosaurus*, a variation of ossification of the cartilaginous parts the proximal end could explain biologically even important variation of shape at this taxonomic scale. However, it can also be the result of taphonomic bone modifications. As with the radius, it is important to be cautious in the biological interpretations that can be drawn from this region.

On the distal end (Fig. 16F), variations of orientation, following the variation of curvature of the shaft, occur. Some variations of outline and of the development are also observed. Because the variations of orientation and outline are slight, they can represent some biologically plausible shape changes. The variation of development is, however, important and can represent either a biological variation or a taphonomic bone modification, as interpreted for the proximal end. Caution is needed in interpreting the variation of this latter feature.

#### Femur

**Figure 17:**
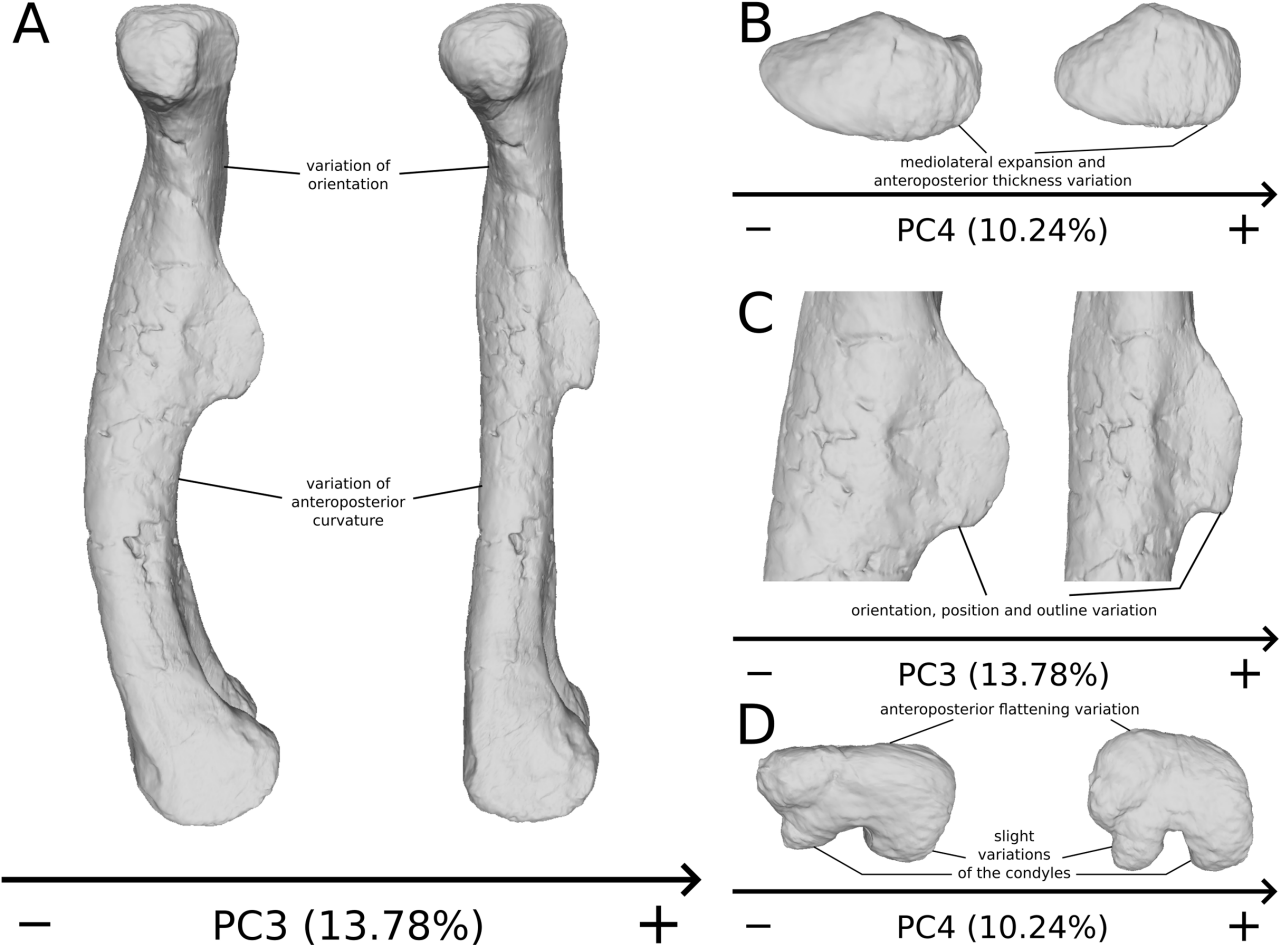
Selected close-ups of biologically plausible femoral variation (i.e., PCs 3, 4 & 6). Variation on PC3 of femoral shaft in medial view (A), on PC4, proximal end in proximal view (B), on PC3, fourth trochanter in medial view (C), and in PC4, distal end in distal view (D). The theoretical shapes corresponding to the minimum of variation along the axis are represented on the left, whereas the shapes corresponding to the maximum of variation are represented on the right.

Due to its complex form (e.g., medially projecting femoral head, presence of trochanters, etc.), the femora of non-sauropod sauropodomorphs can be subject to strong taphonomic patterns. However, the femur also can retain some biologically reliable morphological characteristics:

On the shaft (Fig. 17A), a strong variation of the anteroposterior curvature occurs in the study, from a sigmoid to an almost fully straight shape. Because this variation separates the femora of *Ruehleia* (straight shape) from the femora of *Plateosaurus* and *Efraasia* (sigmoid shape), and does not involve obvious taphonomic patterns such as strong compressions or torsions, it leads us to interpret this strong variation as biological (at an intergeneric level). Furthermore, some slighter variations occur, with minor variations of orientation of the proximal part, associated with an elongation of the anterior ridge extending the lesser trochanter from the proximal to the distal part of the shaft. On the shaft, a slight general variation of curvature and a more important variation of circularity, from a circular to a more eccentric shape, occurs. On the distal half of the shaft, an anteroposterior flattening happens (Fig. 17D). If all of these changes remain biologically plausible, the flattening occurring in the distal end can also be caused by a taphonomic compression of these parts. The variations occurring on the lesser trochanter are mixed with taphonomic deformation occurring on previous obviously taphonomically influenced PCs. It is thus hard to make any biological interpretations on these last two features.

On the fourth trochanter (Fig. 17C), variations of shape outline, orientation, and position occur. If changes of outline and proximodistal position remain slight and more biologically plausible, changes of orientation and mediolateral position are more marked. The variations of position and orientation could be the result of a slight taphonomic compression. They are, moreover, mixed with obvious taphonomically influenced variations, as they occur similarly in the same area. However, because the medial insertion of the caudofemoralis muscles remains preserved (Gatesy, 1990; Langer, 2003; Fechner, 2009; Klinkhamer et al., 2018), these changes remain still biologically plausible, taken cautiously. Considering the variation of outline, two main morphologies are found, from a rounded and smoothly curved shape to sharper and angled one. Some specimens present a distal process that breaks the slope terminating the trochanter. All of the *Ruehleia* femora sampled here present the latter condition, whereas the sampled *Plateosaurus* femora present either the first or the latter morphology.

On the femoral head (Fig. 17B), slight variations of orientation (following variation of orientation observed on the proximal part of the shaft), of mediolateral expansion, and of anteroposterior thickness are observable. Because the variations are slight and do not seem to imply modifications of the femoral head insertion in the hip-joint articulation (which would be unlikely at this given taxonomic scale), these changes can be interpreted as biologically plausible. Because slight variations of orientation are mixed with obviously taphonomically influenced variation, this morphological feature should be interpreted cautiously. Some changes also occur on the greater trochanter, relative to its development, which is relatively well correlated with the development variation of the femoral head.

On the distal end (Fig. 17D), slight variations of outline, width, orientation, and development of the condyles occur. The variations of the width and the orientation seem to follow the variations occurring on the shaft. Depending on the PC observed, the variations linked to morphological variation of the distal part of the shaft can be influenced by the same processes: for example, either biological or anteroposterior taphonomic compression. Any biological conclusion in this area needs to be taken with caution.

#### Tibia

**Figure 18:**
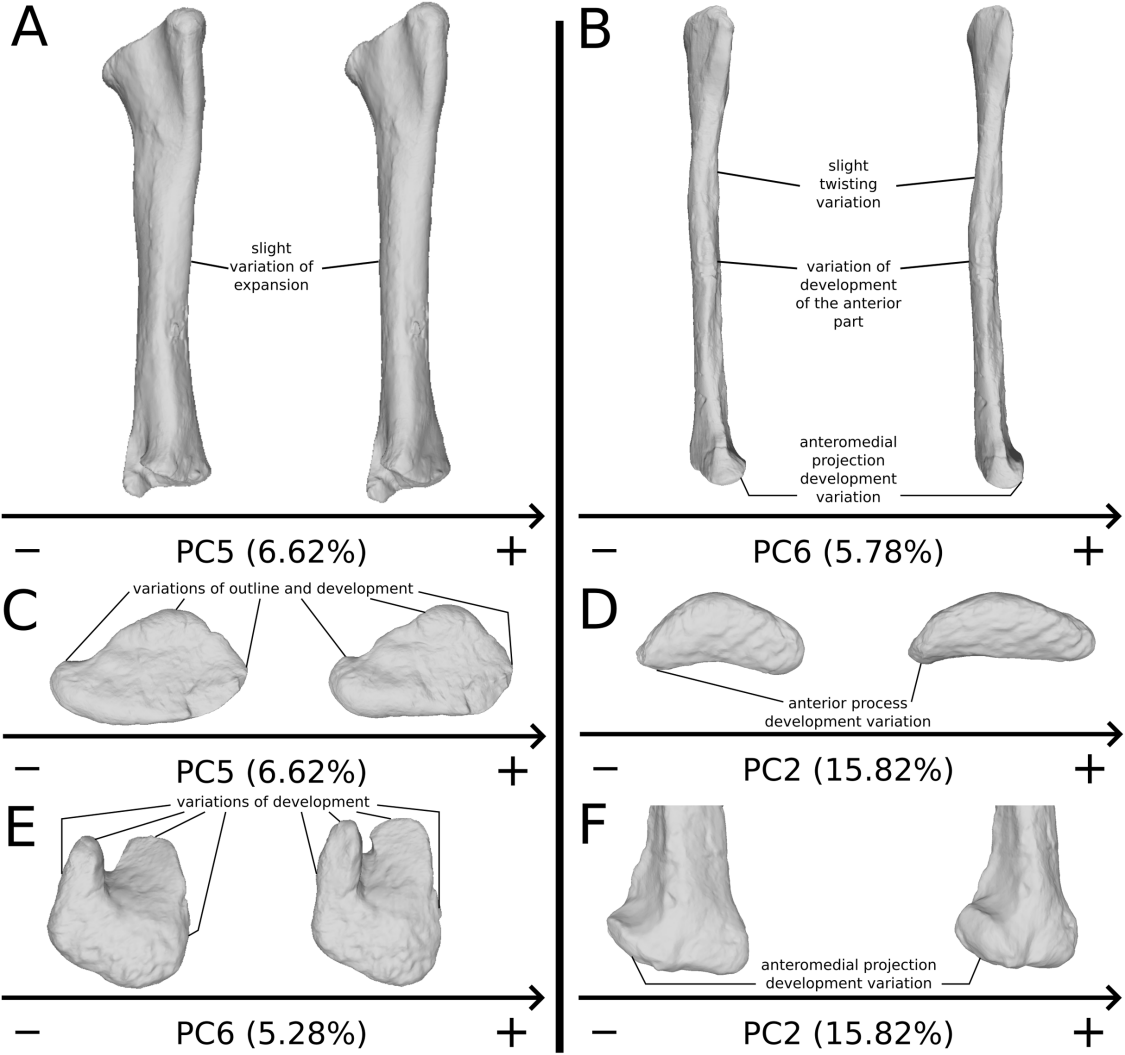
Selected close-ups of biologically plausible tibial (i.e., PCs 5 & 6) and fibular variation (i.e., PCs 2, 6 & 7). For tibiae, variation on PC5 of tibial shaft in anterior view (A) and proximal end in proximal view (C), on PC6, distal end in distal view (E); for fibulae, variation on PC6 of fibular shaft in medial view (B), on PC2, variation of proximal end in proximal view (D), and of distal end in medial view (F). The theoretical shapes corresponding to the minimum of variation along the axis are represented on the left, whereas the shapes corresponding to the maximum of variation are represented on the right.

The shape of the tibia, with a relatively subcircular shaft and complexly shaped proximal and distal ends, is strongly affected by taphonomic patterns, mostly compressions. Once removed, the following biologically plausible morphological features remain:

On the shaft (Fig. 18A), the variation occurs mainly on the orientation of this part of the bone, that is, toward medial and/or posterior directions. Some variations in expansion are also noteworthy. Because these variations occur in an area prominently affected by taphonomic compression, these slighter variations depicted by biologically relevant PCs are mixed with the obviously taphonomically influenced variation.

On the proximal end (Fig. 18C), shape variation is observed on several processes. It occurs globally on the general relative development of this structure, or more specifically on the development of the cnemial crest, the internal condyle, and the complexity of the outline. The development of these specific structures is not necessarily associated with the variation of the general relative development of the proximal end. Additionally, shape variation occurs on the proximal surface of the end, from a domed to a more flattened shape. The orientation of this proximal surface also varies, with its plane anteroproximally orientated when looking in lateral view. Biologically speaking, some slight variations of the development of the cnemial crest and internal condyle seem plausible. All of the other variations, of stronger intensity, could have been influenced by taphonomic variation. Indeed, the changes observed here could take their origin from a taphonomic bending for the orientation of the proximal end and biases linked to differences of ossification or fossilization for the general area, the proximal surface, and some of the variation of development of the cnemial crest. It is thus important to take with extreme caution biological interpretations of this region, because the variation is probably still greatly mixed with taphonomic signal in this study.

The distal end (Fig. 18E), once changes of orientation are discarded, varies in the development of its anterior margin. The surface of the anterior region is variably domed, whereas the posterior margin varies from a convex to a domed outline. Minor shape changes also occur on the development of the medial part and on the posterior margin. Because these changes are slight and do not imply strong changes of shape or orientation of the distal end, they are biologically plausible. However, it could also represent differences of preservation, as seen in the distal ends of other limb bones. These changes should thus be interpreted carefully.

#### Fibula

The fibula is a relatively simply-shaped bone. Some taphonomic variation can occur such as compressions and bendings. The main biologically plausible variation occurs on the following spots:

On the shaft (Fig. 18B), the main biologically plausible variation is a slight general anterior expansion, associated sometimes (but not always) with an inversely correlated posterior expansion. These changes are sometimes also associated with a mediolateral flattening of the shaft, which could indicate a potential taphonomic influence (i.e., compression). Caution on interpretations of these variations are hence needed. A slight variation occurs also around the midshaft region, with a more proximally or distally shifted position of the anteroproximal depression probably corresponding to the iliofibularis insertion (Langer, 2003; McPhee et al., 2014).

On the proximal end (Fig. 18D), relatively strong variation of the anterior part occurs, from a well-developed to an atrophied shape. Because it is also observed on the obviously taphonomically influenced variation, with strong intensity, biological interpretations drawn from this variability should be taken with caution, because it could represent a taphonomic bending of the anterior part of the end. Additionally, variation of development and slight variation of the orientation of the end is also observed in lateral and/or proximal views. Because these variations could be induced by some taphonomic processes, biological conclusions must be cautiously drawn on all these morphological features.

A slight variation of the twisting of the proximal half of the bone is also observable (Fig. 18B), from twisted to nearly straight proximal halves, which could be biological or taphonomic as the orientation variation of the proximal end.

On the distal half, some variation in curvature occurs. Although it might be biological, it can also result from a taphonomic compression, and thus needs to be taken with caution.

The distal end (Figs. 18B and 18F) varies in its global development. A particular variation of the anteromedial projection is noticeable, from undeveloped to developed in the proximal direction. As this variation can be linked to a mediolateral compression, this variation should be taken with caution. Additionally, some variation of the articular outline and development and a slight variation of orientation of the distal end also occur. As for other distal ends, some of these changes may reflect taphonomic bone modifications.

### Interpretation of morphological features: intrageneric variability of sampled bones for *Plateosaurus*

#### Reliability of the interpreted observations as a biological feature

**Figure 19:**
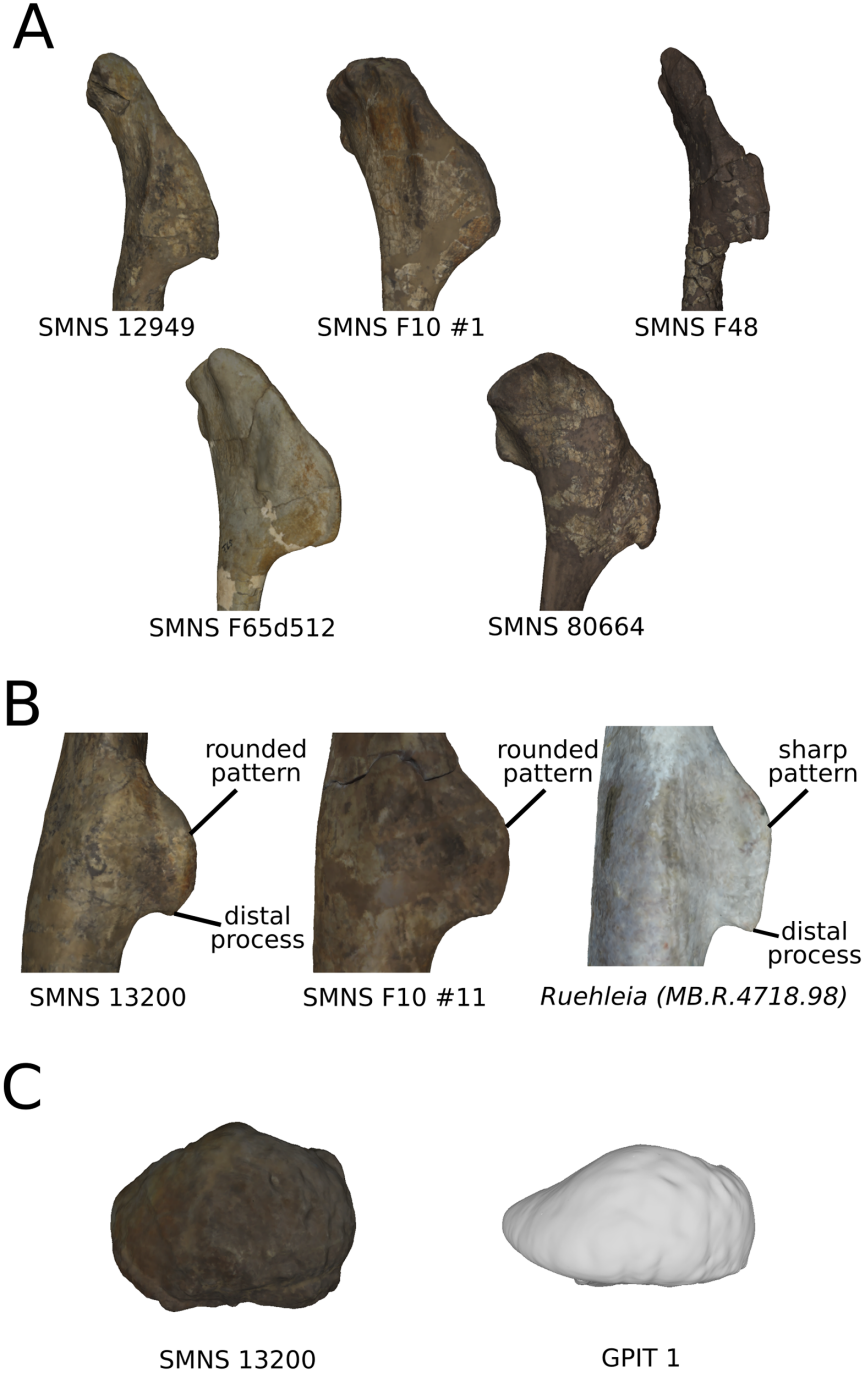
Most biologically compelling variation observed on sampled specimens. Observations of sampled bones for humeral deltopectoral crest outline in lateral view (A), femoral fourth trochanter outline in medial view (B) & head shape in proximal view (C). Not to scale.

The biologically plausible variation occurring in our sample can be classified into three categories, thanks to the corroborative observation of the real sampled specimens:

(1) The most biologically compelling variations occurring in our sample, recognized in our PCs and retrieved in the sampled specimens. All of these features do not seem strongly influenced by any taphonomic processes, with breaks or deformations occasionally present in few specimens, which do not necessarily erase the biological information. For the humerus, this is the case of the deltopectoral crest outline (Fig. 19A) which is highly variable within our sample (Appendix S1 part II). Nearly all of the humeri included in the analysis show a unique combination of character states for this morphological feature. Thus, no clear pattern of distinct morphs can be depicted and any group based on this variation arising from the NJ analysis (Fig. S11).

On the femora, the variation of the shaft is principally driven by the high variability occurring between *Ruehleia* specimens and all the other ones (see below). The depicted variation of the fourth trochanter outline consists of two morphs (Fig. 19B), a rounded one and an angled one, with the presence or absence of a distal process for these two shapes. This distal process, known as the “pendant process”, is also found in some other non-sauropodan sauropodomorphs and many ornitischians (Persons & Currie, 2019). No clear cluster based on these anatomical features is highlighted on the NJ analysis (Fig. S11). All of the specimens sampled present a nearly straight fourth trochanter, with the exception of the left and right femora of GPIT I, which are slightly sigmoid in posterior view. Because this anatomical feature occurs in both femora, it is more plausible that this is a biologically driven feature than a taphonomic one. This trochanter is the attachment of two major muscles (caudofemoralis longus and brevis) involved in sauropodomorph locomotion (notably hip extension; Klinkhamer et al., 2018; Persons & Currie, 2019). Persons & Currie (2019) proposed the hypothesis that the distal elongation of the trochanter with a pendant process resulted in a distal extension of the insertion of the caudofemoralis brevis, conferring better leverage. Thus, the variation observed on this structure could imply subtle changes in the insertion of these muscles, notably the caudofemoralis brevis, hence potentially involving slight variations of locomotion in the genus *Plateosaurus*. The femoral head (Fig. 19C) shows among the sampled specimens some variations of length (from the medial extremity of the head to the most lateral point in proximal view) and width. Two morphs seem to exist, a compact morph (Length/Width around 1.5) and a more expanded one (L/W around 1.9). Considering that each morph has been detected in at least one well-preserved specimen, and that most of the obvious taphonomic deformation for this feature is concentrated in a few specimens, we interpret this morphological variation as biologically compelling. No cluster based on this feature is highlighted by NJ analysis (Fig. S11).

Matching with the “most biologically compelling” features distributions, the NJ complement the description of these traits. It permits evaluation of the importance of the highlighted characters regarding the total kept variation. If a character state distribution is matching with clusters defined in the NJ, we interpret that the variation of this trait is linked to the formation of these clusters. It could thus reflect a higher-than-individual scale variation, which could be linked to a categorical factor (e.g., sex, size). In our study, no “most biologically compelling” morphological feature matches with its corresponding NJ analysis. Several explanations can be assessed to explain this absence of congruence: (1) the variability of the observed character is highly variable among the sample, reflecting an individual variation, not necessarily linked to any categorical factor; (2) the variability is linked to a categorical factor but the majority of the original information is lost with taphonomic deformations, and the residual biological signal is too weak to drive the clustering analysis of the sample; (3) the variation is too slight as compared to the general variation to drive the clustering analysis. It can explain the case of the variation of the deltopectoral crest outline, for which all the sampled specimens display a nearly unique combination of character states. The case of different morphs observed in the fourth trochanter and the femoral heads seems to represent a subtle part of the total variation interpreted as more biologically plausible.

**Figure 20:**
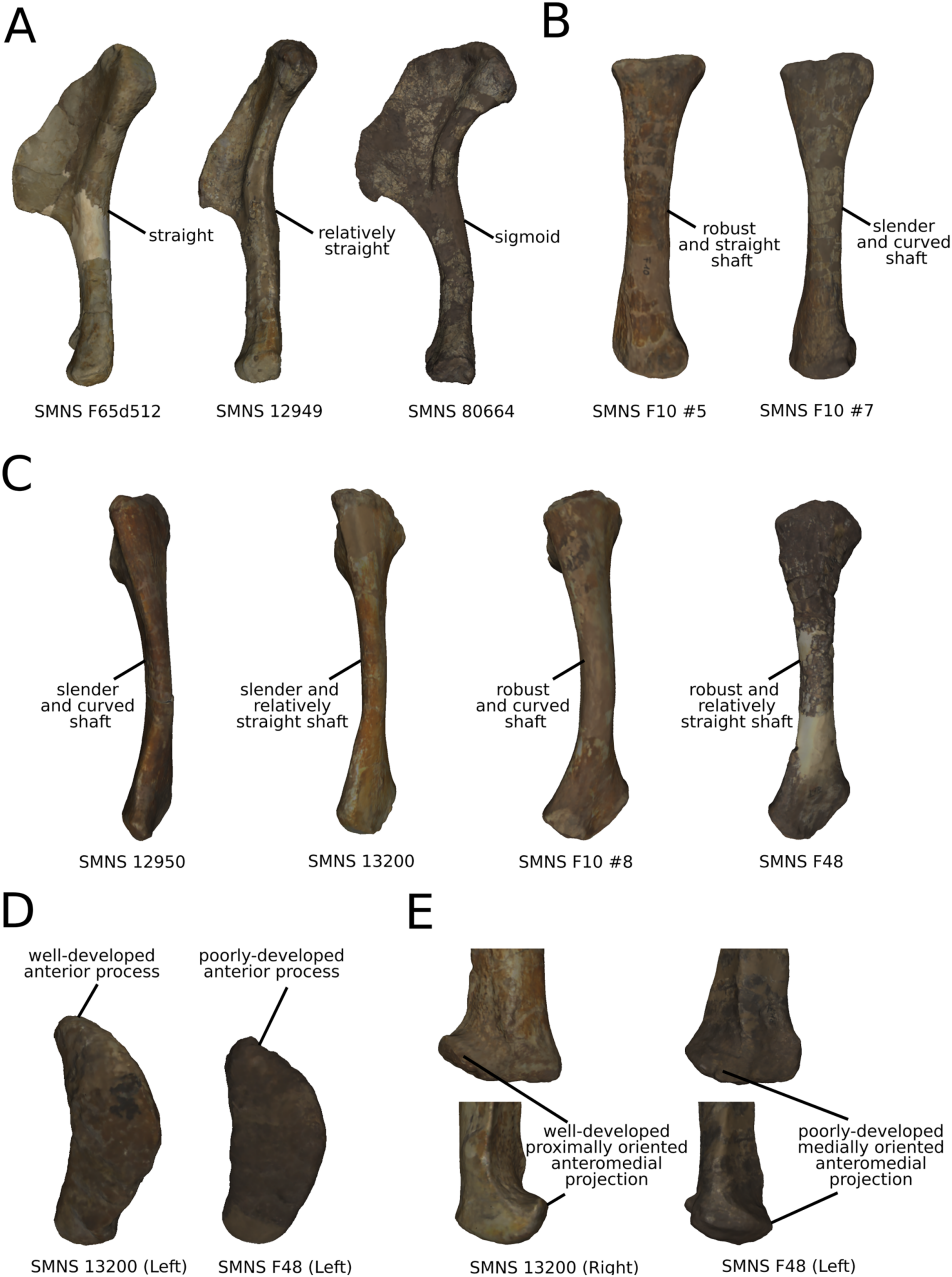
Less biologically compelling variation observed on sampled specimens. Observations of sampled bones for humeral shaft shape in medial view (A), radial shaft shape in posterior view (B), ulnar shaft shape in posterior view (C), fibular proximal end in proximal view (D) & distal end (E) in medial (top) and posterior view (bottom). Not to scale.

**Figure 21:**
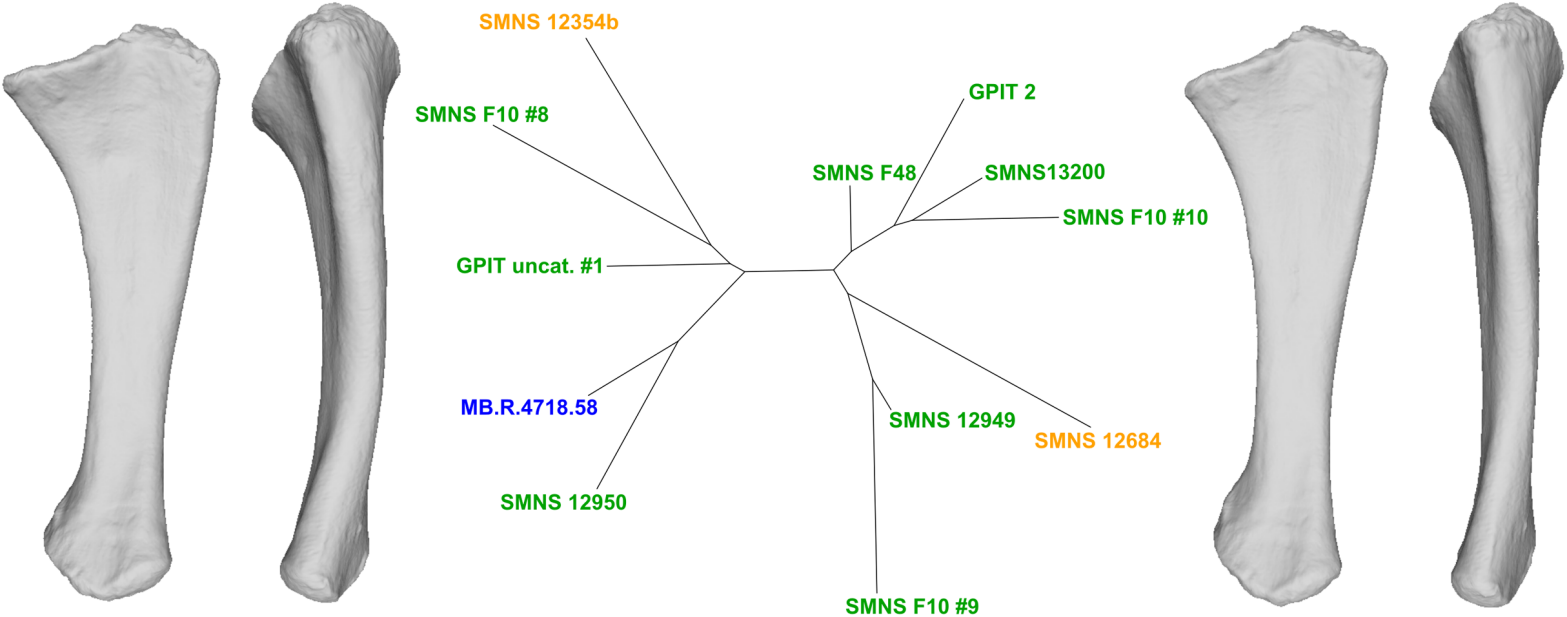
NJ clustering analysis on the biologically plausible variation of the ulna (PCs 4-7). The displayed theoretical shapes correspond to the two subgroups discriminated by the analysis, showing the mean variation between each groups.

(2) The less biologically compelling variations occurring in our sample, recognized in our PCs but not clearly retrieved in the sampled specimens, due to interferences of this information by taphonomic deformations. This is the case for the humeral shaft (Fig. 20A), which shows a sigmoid to straight shape. All of the bones that present a straight shaft are relatively robust, with a robustness index (i.e., proximodistal length/minimum circumference; see Peyre de Fabrègues & Allain, 2016) under 2.60 (Appendix S1 part II). The opposite statement is not true, as some robust bones have a sigmoid shaft. Although the sigmoidal variation of the humerus can be biological, it can be accentuated by taphonomic deformations, notably bendings and torsions (see above). An inspection of the sampled specimens does not permit exclusion of such a possibility. This is also the case for the radius shaft variation (Fig. 20B), with the detection of two potential morphs, a robust one and a more slender one. This kind of variation is seen in many dinosaurs and is generally interpreted as sexual dimorphism (see below). The detection of these two morphs was potentially associated with a variation of curvature. For instance, the slender radii show a well-curved shaft, and the robust ones a nearly straight shaft. These assertions are, however, mixed with taphonomic information occurring in an important number of sampled bones. The existence of two morphs among the bones not showing strong taphonomic influence (with the bulkiest radii showing a robustness index between 2.38 and 2.60 and most slender radii with an index between 2.80 and 3.05) tends to be confirmed by looking back to the sampled specimens. It is, however, not possible to confidently confirm the association with the intensity of curvature, because not all of the bulky radii not show straight or nearly straight shapes (Appendix S1 part II). NJ analysis does not show clear clusters based on these shaft characters (Fig. S11). A similar pattern of robustness is observed with ulnae (Fig. 20C), with two groups: a slender one (robustness index spread around 2.9) and a bulkier one (robustness index from 2.3 to 2.6) (Appendix S1 part II). Moreover, a distinction can be made with the curvature of the shaft of these ulnae, as seen in the PCs, from well-curved shafts to nearly straight ones. This is supported by the NJ clustering (Fig. 21), which clearly discriminates two groups. The ulnae of the first group are clearly more curved than those of the second one. Given the congruence of variation of robustness of the radius and the ulna, it gives more weight to the biological reliability of this feature. Indeed, it seems unlikely that a general circumference variation occurs in only one bone (radius or ulna) and not both at the same time. On the fibulae, a noticeable variation of development of the anterior part of the proximal end is detected in the analysis (Fig. 20D). This signal is, however, mixed with an important part of taphonomic variation occurring in the same area. Biological interpretation of this variation is hence not evident. Indeed, the putative less important development of this part in some specimens is confounded with obvious taphonomic compression of the same area in some others ones. Considering the NJ analysis, the two fibulae belonging to SMNS F48 plot together and present little developed anterior part of the proximal end, which give more support to a biological explanation of this variation (Fig. S11). The morphological features on the shaft seems linked with obvious taphonomy on the specimens, with the exception of the relative position of the probable iliofibularis insertion. Detected on the last PC of the analysis, the variation is too slight to distinguish two or more groups according to this parameter. Biologically speaking, such a variation potentially implies subtle variations in the moment arm of the iliofibularis muscle, notably involved in knee flexion (Langer, 2003) and hip extension (Klinkhamer et al., 2018). Again, a more distally placed insertion would give a better leverage action of the muscle. On the distal end (Fig. 20E), the main variation occurring is the variation of development of the anteromedial projection. This developed process on the distal end of the fibula has been described only in *Sefapanosaurus zastronensis* (Otero et al., 2015). In our sample, it varies from a well-developed and proximally oriented projection to a less developed and more medially oriented projection. No clear cluster based on this feature is found, although the left and right bones that present a less developed shape tend to group more closely together (Fig. S11). The taphonomic signal (mostly deformations) in the structures seems more linked to a few extreme outliers or to occur in a large number of specimens, as compared to the “most biologically compelling” variations.

Matching with the distribution of a “less biologically compelling” feature, the NJ consolidate the biological assertion interpreted from this dubious variation. If the hypothesis that a slight taphonomic pattern common to an important part of the bones could drive the clustering is possible, such a scenario seems a priori less plausible than a biological driving of the clustering, because the NJ is done only on the variation free of obviously taphonomically influenced variation. In our study, for the ulnae, the curvature variation is consolidated by the NJ performed on the most biologically plausible variation (Fig. 21). This reinforces our interpretation as viewing this feature as biological, reflecting a plausible biological dimorphism.

(3) Too much biologically putative variations, on which residual taphonomic bone modification occupies a much too important part of the observed variability in the analysis. This is the case for all of the morphological features of the humeral, radial and ulnar ends, femoral distal end, tibial ends and shaft. The taphonomic bone modifications for these structures seem to affect significantly almost the totality of the specimens for these anatomical features.

To summarize, some biological variability remains among the bones with different degrees of reliability, after removing the most obvious taphonomic one. The tibia is, in the case of our study, the only bone where all the investigated morphological features are too dubious to make any conclusive biological interpretations, regarding the omnipresent part of obvious taphonomy affecting the majority of the specimens. Some other variation is less easily discernible from taphonomic deformation in the remaining variation in the humeri and zeugopod bones, that is, on the radii, ulnae and fibulae. The most plausible variation is principally observed in some morphological features notably present in stylopod bones, that is, the humeri and femora.

#### Additional information of the neighbor-joining analysis

The NJ clustering analysis can be informative in the cases where the left and right bones of the same individual(s) are sampled. A close clustering of these bones provides a good indication that these two bones are probably not very affected by taphonomy. *A fortiori*, this indication permits assessment with a higher degree of confidence that the observed variation is biologically driven. Indeed, the probability that an identical break or any other taphonomic variation occurs similarly in the left and right bones of the same individual seems a priori unlikely such as in the case of the femora of GPIT I and of the fibulae of F48 (Fig. S11). On the other hand, an absence of close clustering would indicate a possible residual taphonomic influence, which would lower the confidence of the biological reliability of the analyzed variation. This is the case for the tibiae and fibulae of SMNS 13200 and GPIT I.

#### Influence of size on observed shape variation

The ulna is the only bone significantly variating with size found in our study, when considering the aligned landmark conformations (Table S2). The results obtained for correlation tests looking at each PC are more nuanced: the PC1 of the humerus is obviously taphonomically influenced, which is also the case for PC1 of the ulna. For the variation of PC1 in the ulna analysis, size variation is clearly influenced by the general flattening highlighted along the axis, whereas for PC1 of the humerus it seems linked to the flattening and deformation of the deltopectoral crest. The link between taphonomy and size variation, however, remains unclear, as the significant effect is not generalized to every PC1 of the six analyses (i.e., the strongest obviously taphonomically influenced variation patterns). Instead, a bone-specific response occurs, maybe depicting for each type of bone a difference of sensitivity to taphonomic influence. Concerning the more biologically plausible variation, the impact of size on PC3 of the femur seems associated with the intergeneric shape variation depicted along the axis. Concerning PC7 of the fibula, the changes are extremely slight and do not seem to depict a strong, clearly identifiable variation (c.f. variation of the relative iliofibularis muscle probable insertion on the fibula). An assessment of any causality seems here too speculative.

### Intrageneric variation of *Plateosaurus*

The “most compelling” and “less compelling” morphological features depicted above constitute the most reliable range of intrageneric variation assessed in our *Plateosaurus* limb long bone sample, taking taphonomic bias into account. Our study highlights two morphs, a robust and a more slender one, for the radii and ulnae and, less obviously, for the humeri. The presence of a robust and a more slender morph is a result also seen in other dinosaur studies, often used as an argument for sexual dimorphism (Raath, 1990; Larson, 2008). It is, however, surprising to find such a pattern on the forelimb bones only. Indeed, several studies which show dimorphism in dinosaur appendicular skeleton have been performed on femora (Raath, 1990; Weishampel & Chapman, 1990; Schweitzer, Wittmeyer & Horner, 2005; Barden & Maidment, 2011). In the case of *Plateosaurus*, Weishampel & Chapman (1990) have found the existence of two clusters not related to size. These two clusters were characterized by the correlation of four linear measurements: the distal femoral width and the caudofemoralis longus attachment site width correlated positively together, in opposition to the femoral distal breadth and the femoral proximal width. By comparing these results with ours, we also highlight the variation of the width of the proximal end, with a relatively high degree of confidence in the biological nature of this feature. Our study also highlights to a certain extant the variation of the caudofemoralis longus attachment site width in our study, as this site is located on the medial side of the fourth trochanter. Therefore, all the shape variation described above for the fourth trochanter implies variation of this measurement at the same time. Thus, this variation of the width measurement implies the same caution on the biological interpretation that we have taken in our study. Finally, the variation of the distal end detected by Weishampel & Chapman (1990) is not discussed here. Indeed, we chose not to interpret biologically the variations occurring in this area, as the potentially biological information was mixed with some taphonomic signal. This taphonomic signal may not have been detected by the linear measurement approach used in Weishampel & Chapman (1990). This suggests that a linear measurement approach might not be reliable to support the biological origin by dimorphism in *Plateosaurus* femora. However, such results also could have not been retrieved because the sample in our study for the femora is limited. A consideration of a larger sample of specimens would permit to conclude on this assertion.

In the case of other dinosaur geometric morphometric studies, Barden & Maidment (2011) have found on *Kentrosaurus* femora some variation on the proximal end, interpreted as sexually dimorphic. In our study, some variations are observed in the greater trochanter, linked to the more global variation of the shaft circularity. Indeed, they are not clearly retrieved when returning to the sampled specimens. A larger sample of better-preserved specimens would possibly permit a conclusion about this result. Because *Plateosaurus* is generally interpreted as a habitual biped (Bonnan & Senter, 2007; Mallison, 2010a), we could assess that the forelimb of this dinosaur is less constrained than the hindlimb. Thus, it appears logical to see a greater biological variation in the forelimb than in the hindlimb elements. More generally, biological explanation of dimorphism in *Plateosaurus* is rather difficult. The variation depicted herein cannot be linked to ontogeny. Indeed, this genus is known to show high developmental plasticity, so that there is no correlation between size and age of the animal (Sander & Klein, 2005; Klein & Sander, 2007). Moreover, given the taphonomic nature of the *Plateosaurus* bonebeds, juveniles were preserved in only a few exceptional cases (Sander, 1992; Hofmann & Sander, 2014). Considering sexual dimorphism, although dimorphism of shaft robustness is traditionally seen as sexual dimorphism, Bonnan, Farlow & Masters (2008) have shown that dimorphic variations on an extant sample of *Alligator mississipiensis* can be hard to attribute to sexual dimorphism without knowing a priori the sex of the specimens. This variation, although existent, can be indeed mixed with individual variation. Moreover, Klein & Sander (2007) found no histological variation linked to sexual dimorphism in *Plateosaurus*. In a more general way, Mallon (2017) argues that even if dimorphism in a dinosaur population is statistically well-established, it is not a sufficient argument per se to characterize it as sexual, because confounding factors (e.g., individual variation, taxonomy, ontogeny, taphonomy) can be the reason of such a variation. The identification of females by positive evidence, such as the presence of medullary bone (Schweitzer, Wittmeyer & Horner, 2005), would permit robust assessment of sexual dimorphism. Given our sample size and the important influence of taphonomy, any definitive assessment of sexual dimorphism seems thus too tentative.

### Intergeneric variation: the cases of *Ruehleia* and *Efraasia*

#### Ruehleia

**Figure 22:**
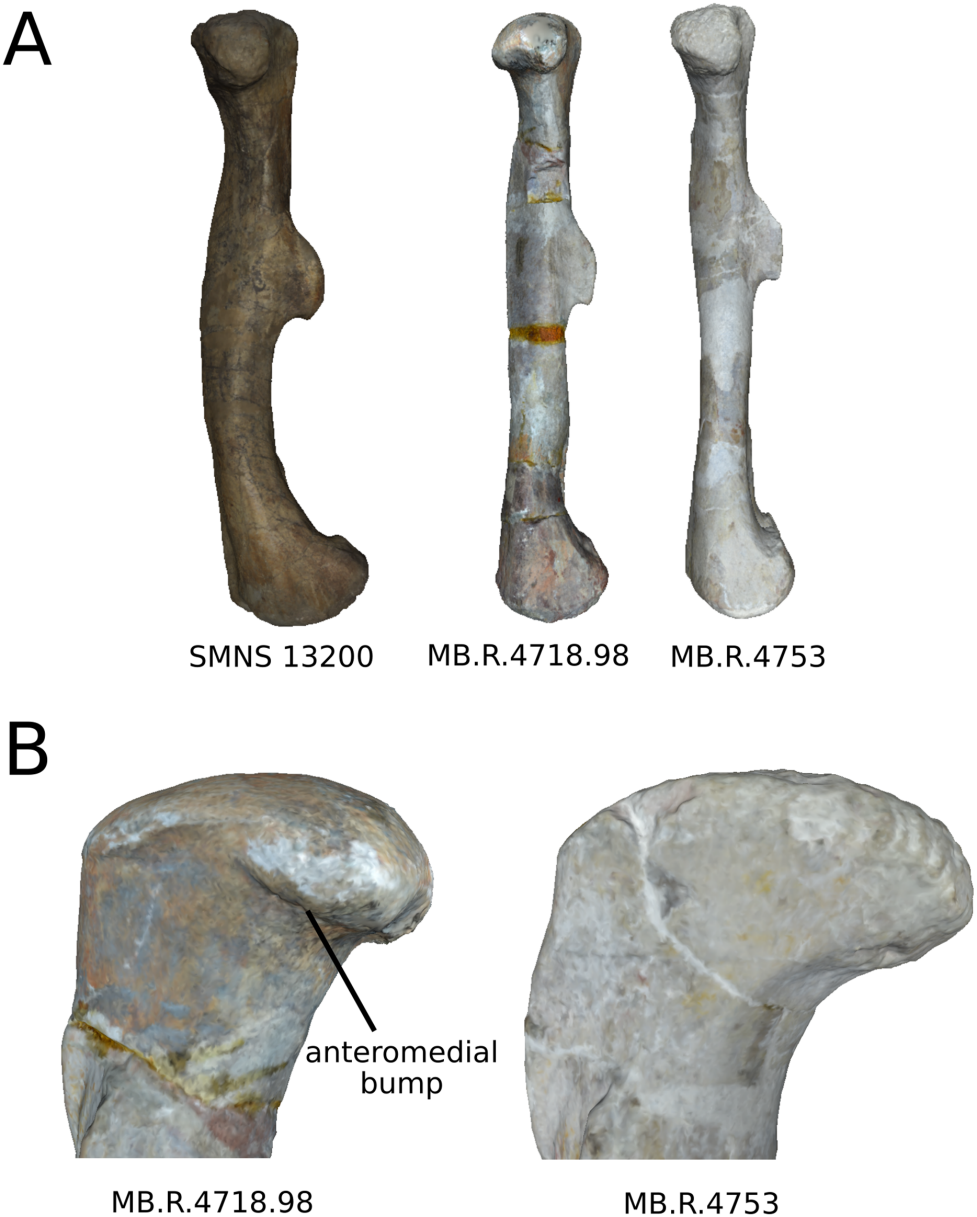
Intergeneric variation occurring between *Plateosaurus* and *Ruehleia*. Observations of sampled specimens of the variation on femoral shaft curvature between SMNS 13200 (*Plateosaurus*) and the two sampled specimens of *Ruehleia* in medial view (A), and variation occurring between the two sampled of *Ruehleia* femora on femoral head in anterior view (B). Not to scale.

As seen in the femora analysis, we detect an important intergeneric signal delimiting *Ruehleia* from the other sampled specimens. The main anatomical feature characterizing their femora is a straight shaft in medial and lateral views (Fig. 22A). We have interpreted this variation as biologically reliable because this variation occurs along the anteroposterior axis and is not correlated in our analysis with variation in strong general flattening. Moreover, the sigmoidicity can be altered by taphonomic influence but is never totally removed. Even in the extreme case of the anteroposteriorly flattened femora of *Sarahsaurus* (Marsh & Rowe, 2018), residual patterns of sigmoidicity are still observable. The examined femora belonging to *Ruehleia* are totally straight in medial and lateral views, which means that there was not any pre-existent sigmoidicity in this sauropodomorph. This observation is corroborated by the two sampled specimens (MB.R4718.98 and MB.R4753) and the incomplete right femur MB.R4718.99 (belonging to the same individual as MB.R4718,98). Straight femora are widely found in sauropods, and straight patterns are also seen in anterior view in some non-sauropodan sauropodomorphs such as *Melanorosaurus* (Yates et al., 2009). The femora of *Ruehleia* are, however, slightly sigmoid in anterior view, when looking at their lateral margin). Some patterns, such as the angled shape of the fourth trochanter (Fig. 19C) and some variation of circularity, are also noticeable. Our study supports the splitting of Galton (2001a), by highlighting the straight shaft as a main character of the appendicular skeleton of *Ruehleia*, which was, however, not used in the diagnosis of the genus. No clear anatomical feature characterizing *Ruehleia* has been found in analyses of the radius and ulna. The mediolaterally straight shafts of *Ruehleia* femora are a relatively surprising finding, because the shaft of the “core prosauropods” (non-sauropodiforms sauropodomorphs) is traditionally considered as sigmoid in lateral view (Galton & Upchurch, 2004). The straightening of the shaft is, moreover, supposed to be linked to a trend to graviportality and obligate quadrupedalism in sauropods and some non-sauropod sauropodiforms (Yates et al., 2009). Such an observation is uncommon in presumed ordinary bipedal sauropodomorphs. Contrasting with the non-sauropod sauropodiform *Meroktenos*, also inferred as probably biped with a straight shaft (Peyre de Fabrègues & Allain, 2016), *Ruehleia*’s femora do not display the same low robustness index and high eccentricity values. Moreover, the two femora used in this analysis show relatively strong differences of eccentricity and femoral head shape (Appendix S1 part II). Indeed, the specimen MB.R.4753 reaches an eccentricity between that of *Melanorosaurus* and all the “core prosauropod” (Peyre de Fabrègues & Allain, 2016), and does not present a anteromedial bump seen in the holotype right femur of *Ruehleia* used in this analysis (Fig. 22B; This bump is less marked in the left femur of the holotype, suggesting maybe preservation biases or a pathological accentuation of the feature on the right femur). Thus, it is possible that these two femora belong to separate taxa. Consequently, a revision of the material attributed to *Ruehleia* seems necessary.

#### Efraasia

Due to the large amount of taphonomic deformation occurring on material of *Efraasia*, no information concerning the expected intergeneric variability relatively to *Plateosaurus* has been found. Moreover, the different NJ clustering analyses tend not to cluster them together. This result can be interpreted as: (1) an absence of limb long bones characters clearly separating the genus *Efraasia* from *Plateosaurus*; (2) the limb long bones characters permitting differentiating *Efraasia* from *Plateosaurus* are correlated with strong taphonomic variation that we removed in our analysis, and no conclusion concerning the variation of this genus could be done by our approach. A revision of the material referred to this genus and from *P. gracilis* (Hungerbühler, 1998; Yates, 2003) would be helpful to clarify this case.

Given these results, it clearly appears clearly that potentially an intergeneric variation can be identified if the taphonomic influence on the sampled specimens is sufficiently low. Again, it shows that the management of taphonomy is critical. Consequently, using strongly deformed specimens such as most of the known material of *Efraasia* in quantitative analyses should be avoided, and these should be analyzed in qualitative analyses taking taphonomy into account.

## Conclusion and Perspectives

The main objective of this study is to evaluate the possibility of quantitatively identifying biological shape variation of a sample of fossil bones after assessing the influence of taphonomy. Using 3D geometric morphometrics, supported by principal component analysis, thin-plates splines and neighbor-joining clustering, we have shown that the variation is dominated by strong, obviously taphonomic patterns. However, even on morphological features influenced by taphonomic processes, the analysis of the uncorrelated variation permits minimization a posteriori of the taphonomic signal by looking only at the most biologically plausible variation. The highlighted anatomical features were categorized according to their reliability as a biological variation: first, the most compelling ones present low taphonomic influence. The use of NJ clustering analysis permits highlighting of the importance of a feature as a driver of the separation of two or more groups in the sample. Second, the less biologically convincing variation, although legitimately found as biologically plausible, is confounded with some taphonomic variation. This influence gives a lesser degree of confidence to these features. The biological plausibility of these features can be, however, reinforced by NJ clustering analysis when they match with the separation of two or more groups. The last category is highly mixed with taphonomic bone modifications (i.e., deformations, breaks, abrasion or preservation biases), when looking back to specimens, such that most of them have taphonomic information obscuring the biological information. Our study has successfully highlighted the most biologically compelling variation, relevant at an intrageneric scale. They may represent a subtle diversity of features illustrating the individual variation, potential dimorphism, or subtle locomotor-related variations. Moreover, the analysis also identified interspecific variation when taphonomic influence was low (e.g., *Ruehleia*). Thus, our study illustrates that 3D GM is a powerful tool for the study of shape variation of fossil bones, even within a small sample affected by strong deformations.

## Supplemental Information

10.7717/peerj.9359/supp-1 Supplemental Information 1 List of landmarks used in this study.

10.7717/peerj.9359/supp-2 Supplemental Information 2 Tests of the size effect on the shape of the bones.

10.7717/peerj.9359/supp-3 Supplemental Information 3 Results of the PCA on the PC1 and PC2 of the repeatability tests. For each bones, three specimens were selected. The set of anatomical landmarks of those specimens has been digitized ten times. On each plot, each specimens are recognizable by their color. For all the bones, the inter-specimen variation was gretaer than the intra-specimen variation.

10.7717/peerj.9359/supp-4 Supplemental Information 4 Results of the PCA on the PC3 and PC4 of the humerus analysis (right side illustrated). On the PCA plot (A), the green cluster represents the morphospace occupied by the genus *Plateosaurus*, the orange dots correspond to the *Efraasia* specimens. Extrema of shape changes along PC3 (B) and PC4 (C) are represented in anterior and lateral views.

10.7717/peerj.9359/supp-5 Supplemental Information 5 Results of the PCA on the PC3 and PC4 of the radius analysis (right side illustrated). On the PCA plot (A), the green cluster represents the morphospace occupied by the genus *Plateosaurus*, the orange dot corresponds to the *Efraasia* specimen, the blue dot corresponds to the *Ruehleia* specimen. Extrema of shape changes along PC3 (B) and PC4 (C) are represented in medial and posterior views.

10.7717/peerj.9359/supp-6 Supplemental Information 6 Results of the PCA on the PC7 of the radius analysis (right side illustrated). On the PCA plot (A), the green cluster represents the morphospace occupied by the genus *Plateosaurus*, the orange dot corresponds to the *Efraasia* specimen, the blue dot corresponds to the *Ruehleia* specimen. Extrema of shape changes along PC7 (B) are represented in medial and posterior views.

10.7717/peerj.9359/supp-7 Supplemental Information 7 Results of the PCA on the PC3 and PC4 of the ulna analysis (right side illustrated). On the PCA plot (A), the green cluster represents the morphospace occupied by the genus *Plateosaurus*, the orange dots correspond to the *Efraasia* specimens, the blue dot corresponds to the *Ruehleia* specimen. Extrema of shape changes along PC3 (B) and PC4 (C) are represented in medial and posterior views.

10.7717/peerj.9359/supp-8 Supplemental Information 8 Results of the PCA on the PC7 of the ulna analysis (right side illustrated). On the PCA plot (A), the green cluster represents the morphospace occupied by the genus *Plateosaurus*, the orange dots correspond to the *Efraasia* specimens, the blue dot corresponds to the *Ruehleia* specimen. Extrema of shape changes along PC7 (B) are represented in medial and posterior views.

10.7717/peerj.9359/supp-9 Supplemental Information 9 Results of the PCA on the PC5 and PC6 of the femur analysis (right side illustrated). On the PCA plot (A), the green cluster represents the morphospace occupied by the genus *Plateosaurus*, the orange dot corresponds to the *Efraasia* specimens, the blue dots correspond to the *Ruehleia* specimen and the brown dot correspond to SMNS 12220. Extrema of shape changes along PC5 (B) and PC6 (C) are represented in anterior and lateral views.

10.7717/peerj.9359/supp-10 Supplemental Information 10 Results of the PCA on the PC3 and PC4 of the tibia analysis (right side illustrated). On the PCA plot (A), the green dots correspond to the specimens of the genus *Plateosaurus*. Extrema of shape changes along PC3 (B) and PC4 (C) are represented in lateral and posterior views.

10.7717/peerj.9359/supp-11 Supplemental Information 11 Results of the PCA on the PC3 and PC4 of the fibula analysis (right side illustrated). On the PCA plot (A), the green dots correspond to the specimens of the genus *Plateosaurus*. Extrema of shape changes along PC3 (B) and PC4 (C) are represented in lateral and posterior views.

10.7717/peerj.9359/supp-12 Supplemental Information 12 Results of the PCA on the PC7 of the fibula analysis (right side illustrated). On the PCA plot (A), the green dots correspond to the specimens of the genus *Plateosaurus*. Extrema of shape changes along PC7 (B) are represented in anterior and lateral views.

10.7717/peerj.9359/supp-13 Supplemental Information 13 NJ clustering analyses on the biologically plausible variation. Analyses performed for the humerus (PCs 4-6), the radius (PCs 4-7), the femur (PCs 3,4 and 6), the tibia (PCs 5-6) and the fibula (PCs 2, 6 and 7).

10.7717/peerj.9359/supp-14 Supplemental Information 14 Summary of interpretations of PCs. The following list enumerates with a brief description the main variations depicted in all the PCs (see results), associated with an interpretation in terms of taphonomic influence. The variation is categorized in three categories: obviously taphonomically ingluenced, ambiguous or biologically plausible. Only the PCs without any obviously taphonomically influenced variation are analyzed in terms of biological discussion. The biological discussion is supported by direct observation of the discrete (character state) or continuous (measurements) features on each analyzed specimen, recorded in tables in the second part of this appendix.

10.7717/peerj.9359/supp-15 Supplemental Information 15 Raw data: coordinates of the anatomical landmarks and curves and surface semilandmarks. The format (.pts) is readable by the packages used in the study and can be opened like a ".txt" file.

## Additional Information and Declarations

### Data Availability

The following information was supplied regarding data availability: The data are available in a Supplemental File. The specimen collection numbers are available in Table 1. The specimens used in this study were accessioned in the German collections from the Staatliches Museum für Naturkunde, Stuttgart (SMNS), Institute for Geosciences, Eberhard-Karls-Universität, Tübingen (GPIT) and Museum für Naturkunde, Berlin (MB.R).
