# Supplementary material for: Disentangling biological variability and taphonomy: shape analysis of the limb long bones of the sauropodomorph dinosaur Plateosaurus
Source: PeerJ. 2020 Jul 23;8:e9359. doi: 10.7717/peerj.9359 (PMC7382942; doi:10.7717/peerj.9359)

**Table S1**

**List of landmarks in this study**

**Number of landmarks per bone**

|  | Anatomical landmarks | Curve sliding semiladmarks | Surface sliding semilandmarks | Total |
| --- | --- | --- | --- | --- |
| Humerus | 19 | 278 | 481 | 778 |
| Radius | 8 | 136 | 210 | 354 |
| Ulna | 8 | 235 | 572 | 815 |
| Femur | 20 | 324 | 501 | 805 |
| Tibia | 14 | 247 | 638 | 885 |
| Fibula | 10 | 217 | 280 | 507 |

**Humerus (following nomenclature of Remes, 2008)**

| # | Anatomical Landmark Definition |
| --- | --- |
| 1 | Most posterior point of the lateral tubercule |
| 2 | Most posterior point of the humeral head |
| 3 | Maximum of concavity between the proximal outline of the humeral head and the medial tuberosity |
| 4 | Most posteromedial point of the medial tuberosity |
| 5 | Most anteromedial point of the medial tuberosity |
| 6 | Most anterior point of the humeral head |
| 7 | Beginning of the deltopectoral crest apex |
| 8 | End of the deltopectoral crest apex |
| 9 | End of the deltopectoral crest (break of slope in medial view) |
| 10 | Most anteromedial point of the ulnar condyle |
| 11 | Most posteromedial point of the ulnar condyle |
| 12 | Most posterior point of the ulnar condyle |
| 13 | Maximum of curvature between posterior outline of the ulnar condyle and the radial condyle |
| 14 | Most posterolateral point of the radial condyle |
| 15 | Most anterolateral point of the radial condyle |
| 16 | Most anterior point of the radial condyle |
| 17 | Intersection between the radial condyle proximal outline and the ventral intercondylar pit |
| 18 | Intersection between the ulnar condyle proximal outline and the ventral intercondylar pit |
| 19 | Most anterior point of the ulnar condyle |


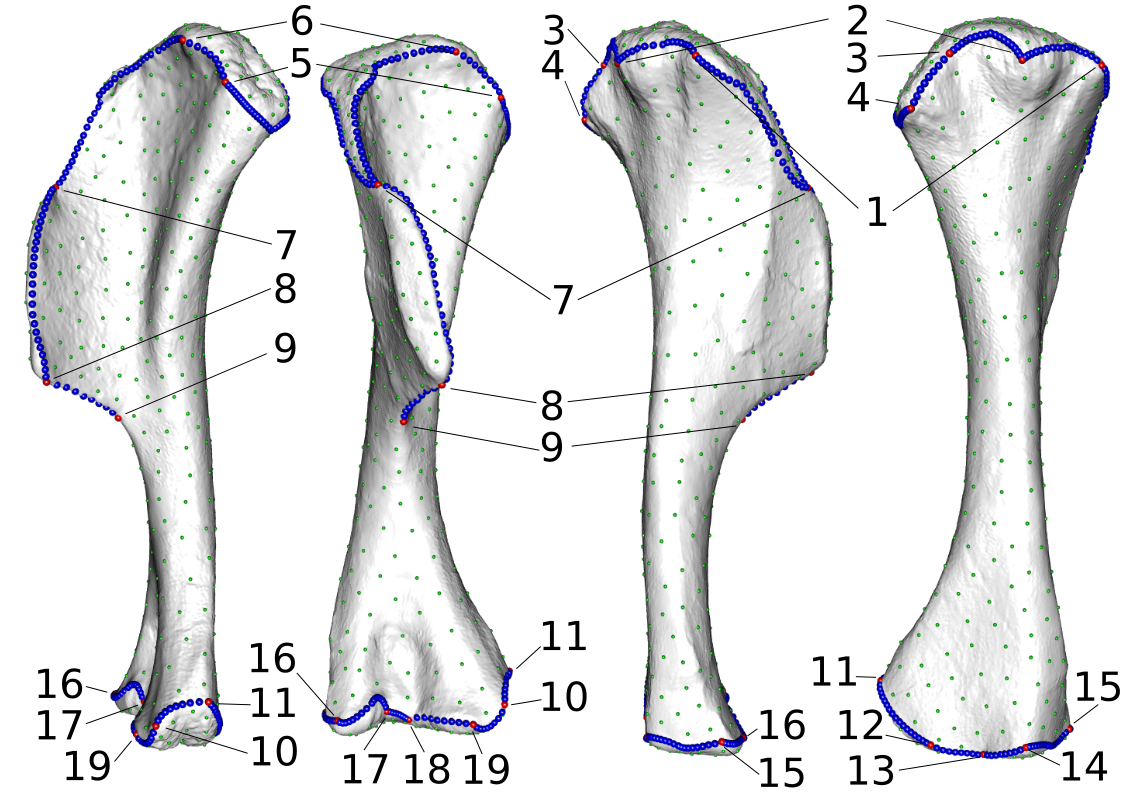


**Radius (mostly following Remes, 2008)**

| # | Anatomical Landmark Definition |
| --- | --- |
| 1 | Most posterior point of the radial posteroproximal process |
| 2 | Maximum of curvature of the medial margin of the proximal end |
| 3 | Most anterior point of the proximal end |
| 4 | Maximum of curvature of the lateral margin of the proximal end |
| 5 | Most posterolateral point of the distal end |
| 6 | Most lateral point of the distal end |
| 7 | Most anterior point of the distal end |
| 8 | Posteromedial tip of the posteromedial process of the distal end |


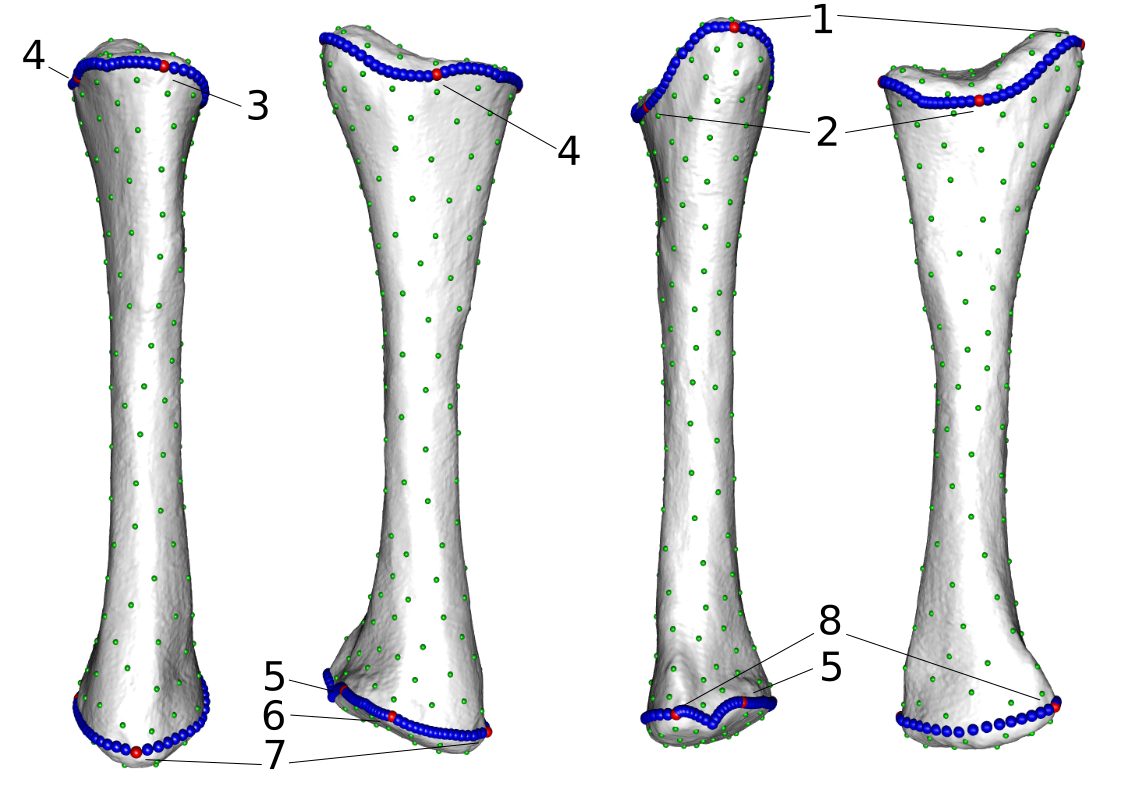


**Ulna (following Remes, 2008)**

| # | Anatomical Landmark Definition |
| --- | --- |
| 1 | Most lateral point of the lateral process |
| 2 | Most dorsal point of the olecranon |
| 3 | Maximum of curvature of the medial margin of the distal end |
| 4 | Most anterior point of the anterior process |
| 5 | Most anterior point of the distal end |
| 6 | Maximum of curvature on the medial margin of the distal end |
| 7 | Most posterior point of the distal end |
| 8 | Maximum of curvature on the lateral margin of the distal end |


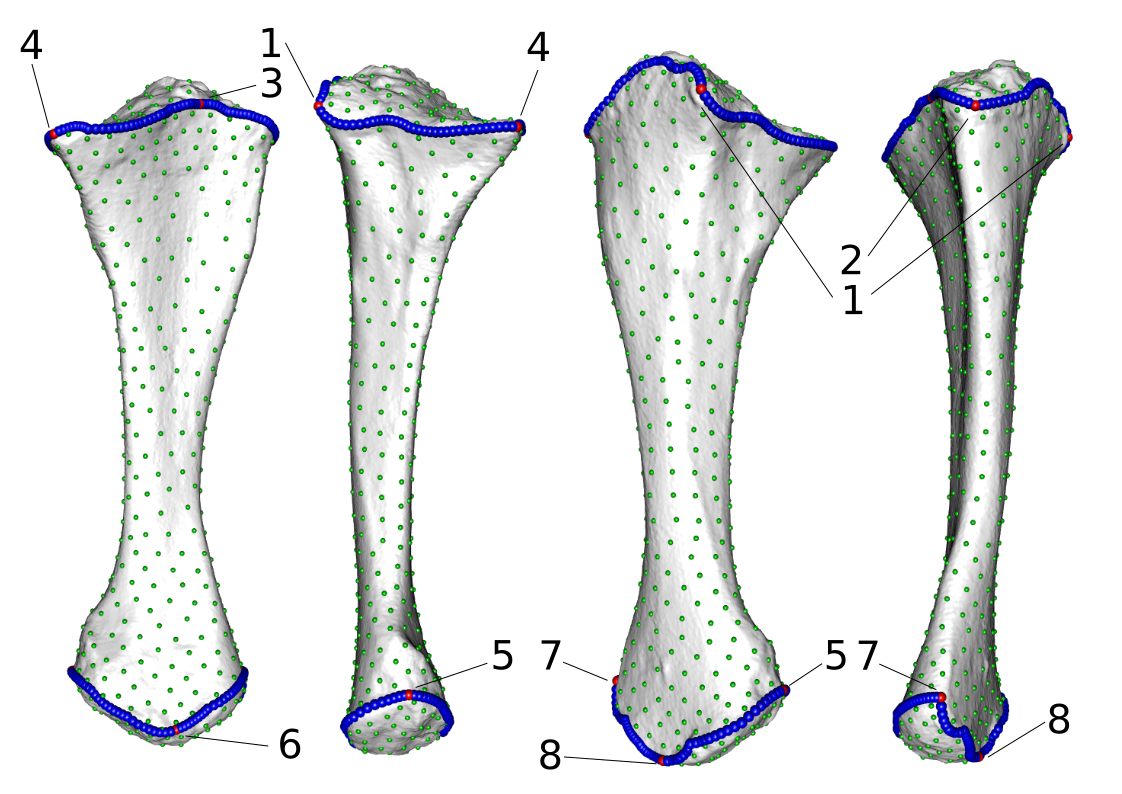


**Femur (following Langer, 2003; Galton&Upchurch 2004)**

| # | Anatomical Landmark Definition |
| --- | --- |
| 1 | Most mediodistal point of the femoral head |
| 2 | Maximum of concavity on the posterior margin between the femoral head and the medial tuber in posterior view |
| 3 | Apex of the medial tuber of the proximal end |
| 4 | Inflexion point on the posterior margin between the medial tuber and the posterior corner of the greater trochanter |
| 5 | Intersection between the posterior margin of the femoral head and the greater trochanter |
| 6 | Intersection between the anterior margin of the femoral head and the greater trochanter |
| 7 | Maximum of concavity on the anterior margin of the femoral head |
| 8 | Beginning of the fourth trochanter |
| 9 | Last distal slope inflexion point of fourth trochanter |
| 10 | End of the fourth trochanter |
| 11 | Maximal proximal extension of the lesser trochanter |
| 12 | Maximal distal extension of the lesser trochanter |
| 13 | Intersection of the medial condyle and the intercondylar fossa on the posterior margin of the distal end |
| 14 | Intersection of the lateral condyle and the intercondylar fossa on the posterior margin of the distal end |
| 15 | Most posterior point of the lateral condyle |
| 16 | Maximum of curvature of the lateral margin of the lateral condyle |
| 17 | Most lateral point of the lateral condyle |
| 18 | Maximum of curvature of the anterior margin of the distal end |
| 19 | Most medial point of the medial of the medial condyle |
| 20 | Most posterior point of the medial condyle |


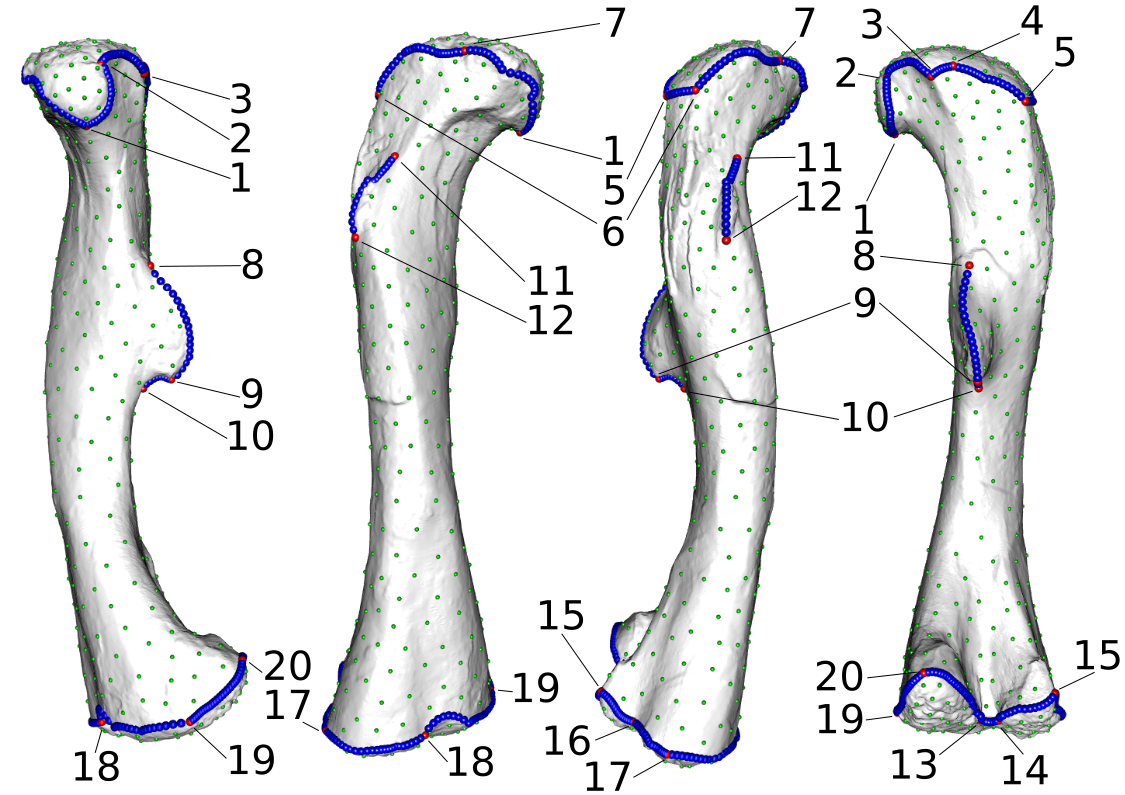


**Tibia (following Langer, 2003)**

| # | Anatomical Landmark Definition |
| --- | --- |
| 1 | Most anterior point of the anterior margin of the cnemial crest |
| 2 | Most lateral point of the cnemial crest |
| 3 | Maximum of concavity between the cnemial crest and the fibular condyle |
| 4 | Most lateral point of the fibular condyle |
| 5 | Maximum of concavity between the fibular condyle and the internal condyle |
| 6 | Most posterior point of the internal condyle |
| 7 | Most anterior point of the ascending process |
| 8 | Maximum of concavity of the anterior margin of the ascending process in anterior view |
| 9 | Most anterolateral point of the ascending process |
| 10 | Maximum of concavity of the lateral margin of the distal end |
| 11 | Most posterolateral point of the distal end |
| 12 | Most anterolateral point of the descending process |
| 13 | Intersection between the ascending process and the tibial mediodistal groove |
| 14 | Tip of the anterior and lateral margins of the ascending process |


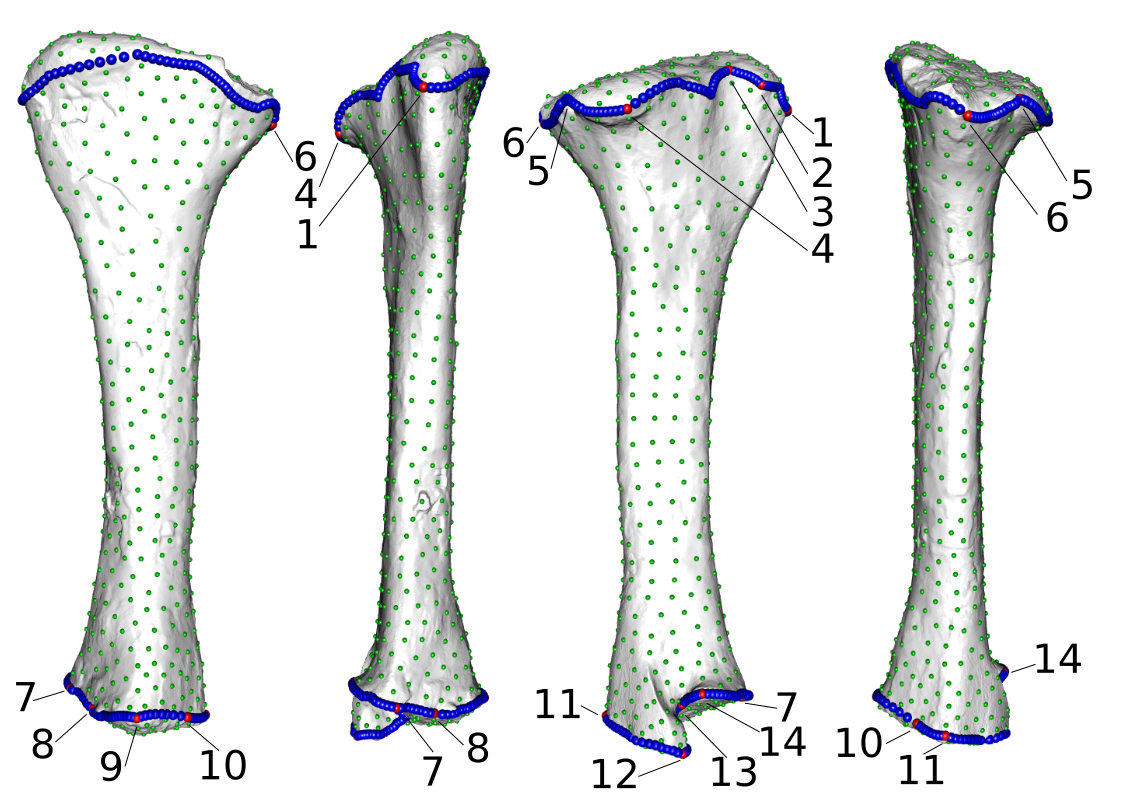


**Fibula (following Langer, 2003)**

| # | Anatomical Landmark Definition |
| --- | --- |
| 1 | Most lateral point of the proximal end |
| 2 | Most posterior point of the proximal end |
| 3 | Most ventral point of the proximal end |
| 4 | Most anterior point of the proximal end |
| 5 | Intersection between the anterior ridge and the distal end |
| 6 | Medial maximum of concavity between the anterior and distal part of the distal end |
| 7 | Most posterior point of the distal end |
| 8 | Lateral maximum of concavity between the anterior and distal part of the distal end |
| 9 | Most lateral point of the distal end |
| 10 | Most proximal point of the distal end |


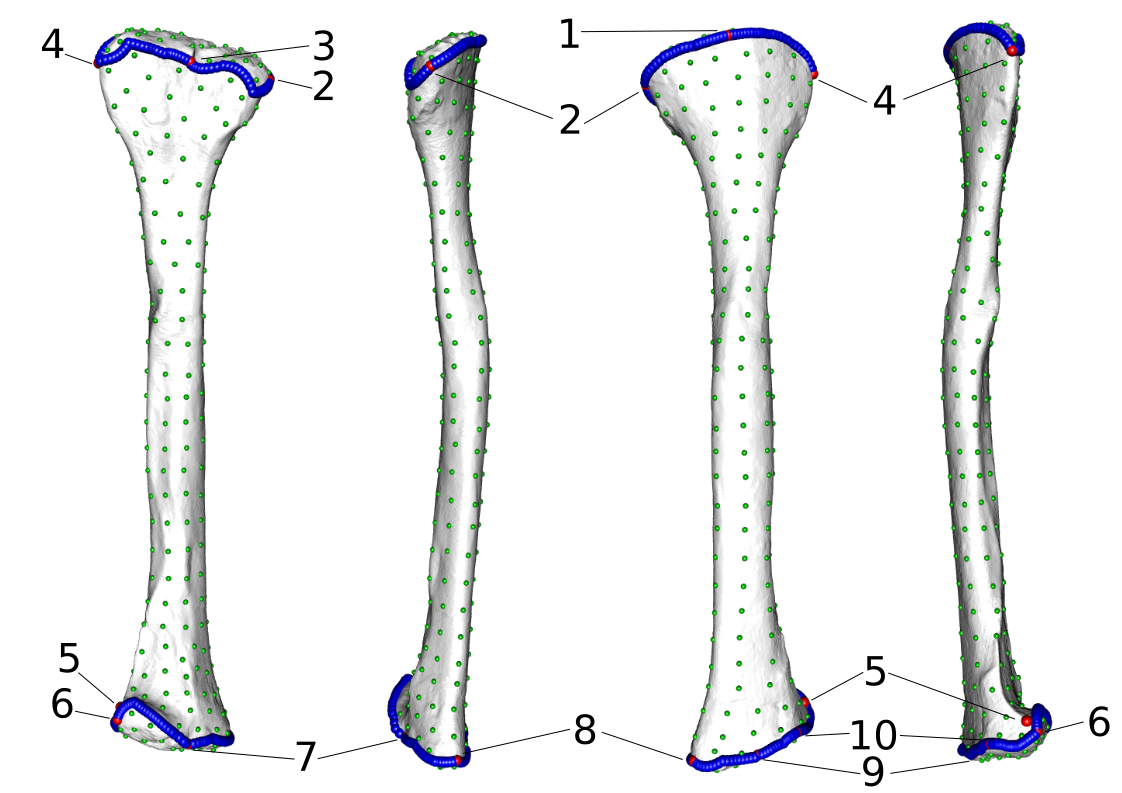

Supplement: Supplemental Information 1 [file peerj-08-9359-s001.docx]
