# Supplementary material for: Disentangling biological variability and taphonomy: shape analysis of the limb long bones of the sauropodomorph dinosaur Plateosaurus
Source: PeerJ. 2020 Jul 23;8:e9359. doi: 10.7717/peerj.9359 (PMC7382942; doi:10.7717/peerj.9359)
Supplement: Supplemental Information 2 [file peerj-08-9359-s002.docx]

**Table 2**

**Tests of the size effect on shape conformations**

Test of the effect of size (*i.e.* natural logarithm of centroid size) on aligned landmark conformations (Procrustes ANOVA with the logarithm of centroid size; significant results (p-value < 0.05) in bold)

| Bone | R² | F | p-value |
| --- | --- | --- | --- |
| Humerus | 0.19458 | 2.1743 | 0.067 |
| Radius | 0.1044 | 1.0492 | 0.373 |
| **Ulna** | **0.21147** | **2.6818** | **0.012** |
| Femur | 019486 | 1.9361 | 0.072 |
| Tibia | 0.09549 | 0.8445 | 0.534 |
| Fibula | 0.05369 | 0.6241 | 0.823 |

Pearson correlation test between the natural logarithm of centroid size and each PC (significant results (p-value < 0.05) in bold)

| Bone | PC | R² | t | p-value |
| --- | --- | --- | --- | --- |
| Humerus | **PC1** | **0.3709** | **2.303** | **0.0467** |
|  | PC2 | 0.02277 | -0.458 | 0.658 |
|  | PC3 | 0.01727 | 0.398 | 0.7 |
|  | PC4 | 0.04998 | -0.688 | 0.509 |
|  | PC5 | 0.1343 | -1.181 | 0.268 |
|  | PC6 | 0.008997 | 0.286 | 0.781 |
| Radius | PC1 | 0.06648 | -0.801 | 0.444 |
|  | PC2 | 0.2801 | -1.871 | 0.0941 |
|  | PC3 | 0.03405 | 0.175 | 0.8647 |
|  | PC4 | 0.006694 | -0.246 | 0.811 |
|  | PC5 | 0.3091 | 2.006 | 0.0758 |
|  | PC6 | 0.01229 | 0.335 | 0.746 |
|  | PC7 | 0.01616 | 0.385 | 0.71 |
| Ulna | **PC1** | **0.4737** | **3** | **0.0133** |
|  | PC2 | 0.1217 | 1.177 | 0.266 |
|  | PC3 | 0.0001776 | 0.042 | 0.967 |
|  | PC4 | 0.02147 | -0.468 | 0.65 |
|  | PC5 | 0.01259 | -0.357 | 0.728 |
|  | PC6 | 0.152 | -1.339 | 0.21 |
|  | PC7 | 0.1891 | 1.527 | 0.158 |
| Femur | PC1 | 0.2985 | -1.845 | 0.102 |
|  | PC2 | 0.02317 | -0.436 | 0.675 |
|  | **PC3** | **0.508** | **2.874** | **0.0207** |
|  | PC4 | 0.01641 | 0.365 | 0.724 |
|  | PC5 | 0.03946 | 0.573 | 0.582 |
|  | PC6 | 0.001382 | -0.105 | 0.919 |
| Tibia | PC1 | 0.08844 | 0.881 | 0.404 |
|  | PC2 | 0.1036 | -0.961 | 0.3645 |
|  | PC3 | 0.09302 | 0.906 | 0.3915 |
|  | PC4 | 0.0446 | 0.611 | 0.558 |
|  | PC5 | 0.05552 | 0.686 | 0.512 |
|  | PC6 | 0.1905 | -1.372 | 0.207 |
| Fibula | PC1 | 0.0002076 | 0.048 | 0.963 |
|  | PC2 | 0.01512 | 0.411 | 0.689 |
|  | PC3 | 0.0005997 | 0.081 | 0.937 |
|  | PC4 | 0.1347 | -1.309 | 0.217 |
|  | PC5 | 0.08765 | 1.028 | 0.326 |
|  | PC6 | 0.1718 | -1.51 | 0.1591 |
|  | **PC7** | **0.3573** | **2.473** | **0.031** |
