# Supplementary figures and images for: Disentangling biological variability and taphonomy: shape analysis of the limb long bones of the sauropodomorph dinosaur Plateosaurus
Source: PeerJ. 2020 Jul 23;8:e9359. doi: 10.7717/peerj.9359 (PMC7382942; doi:10.7717/peerj.9359)

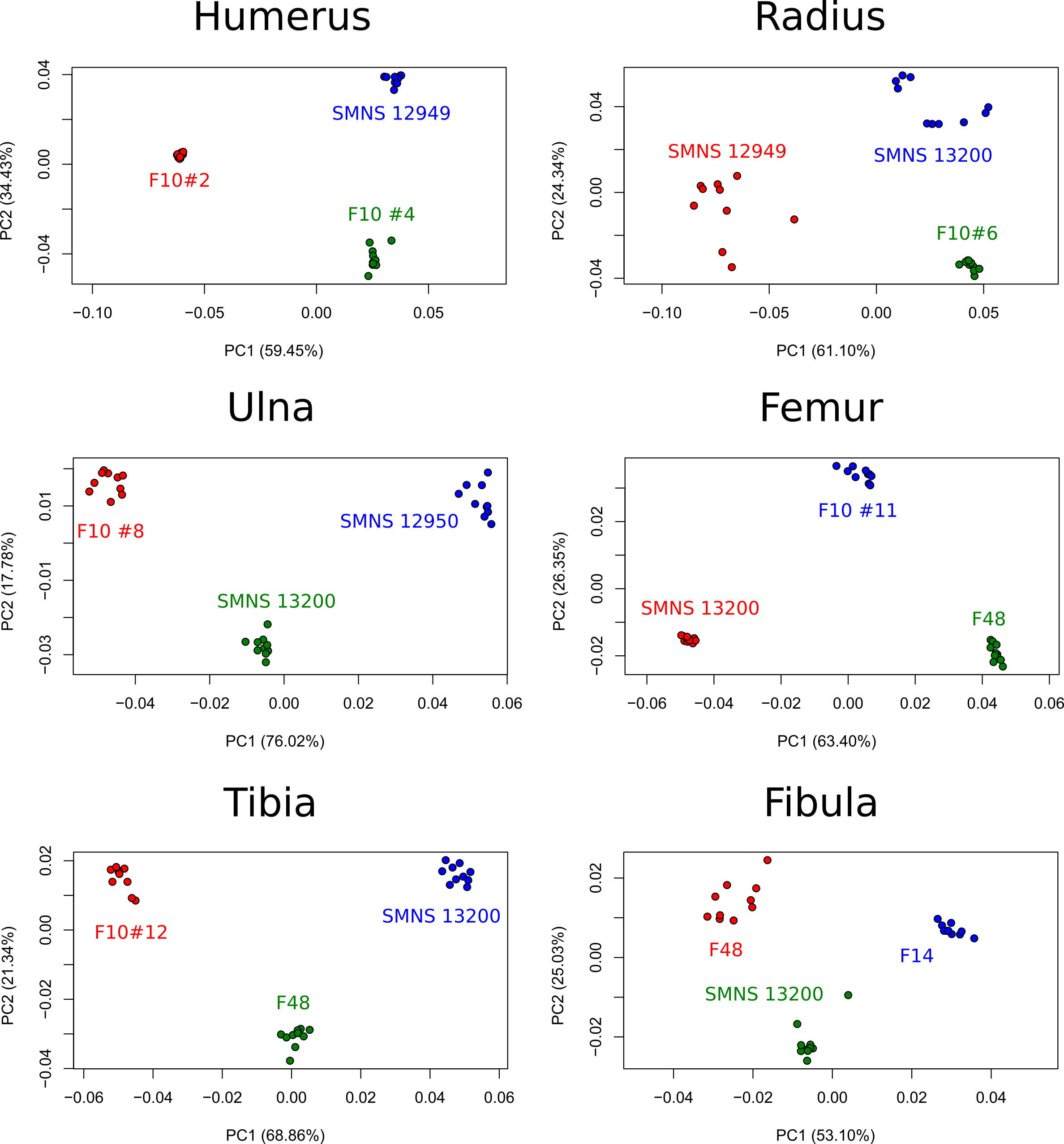

Supplement: Supplemental Information 3 — For each bones, three specimens were selected. The set of anatomical landmarks of those specimens has been digitized ten times. On each plot, each specimens are recognizable by their color. For all the bones, the inter-specimen variation was gretaer than the intra-specimen variation. [file peerj-08-9359-s003.png]

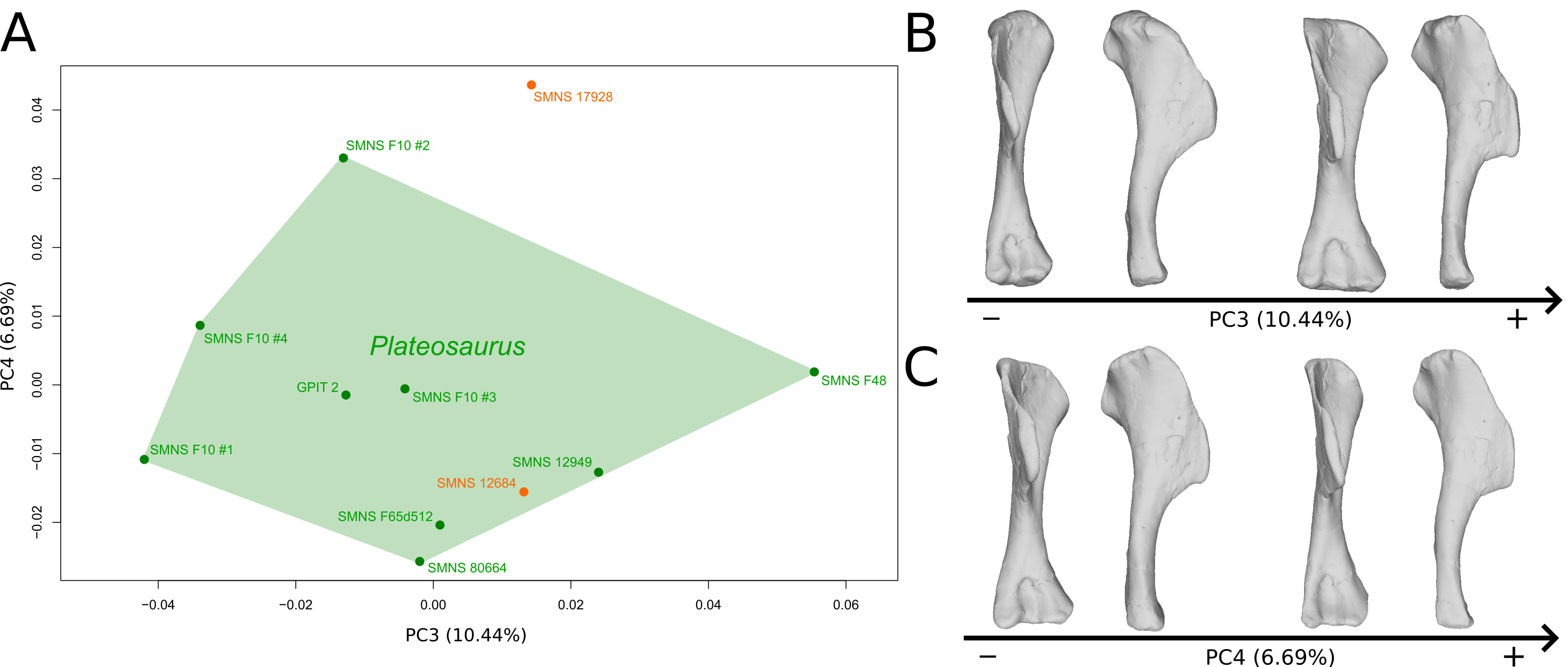

Supplement: Supplemental Information 4 — On the PCA plot (A), the green cluster represents the morphospace occupied by the genus Plateosaurus, the orange dots correspond to the Efraasia specimens. Extrema of shape changes along PC3 (B) and PC4 (C) are represented in anterior and lateral views. [file peerj-08-9359-s004.png]

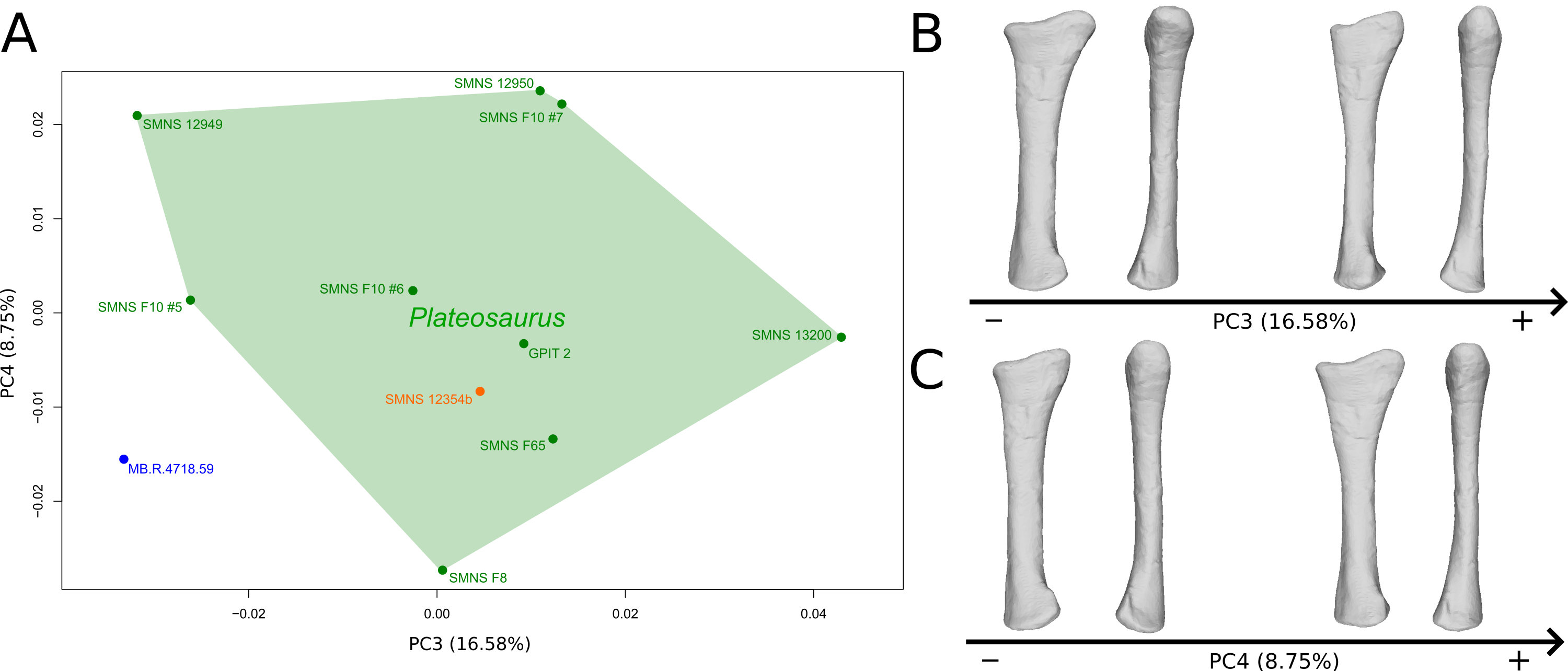

Supplement: Supplemental Information 5 — On the PCA plot (A), the green cluster represents the morphospace occupied by the genus Plateosaurus, the orange dot corresponds to the Efraasia specimen, the blue dot corresponds to the Ruehleia specimen. Extrema of shape changes along PC3 (B) and PC4 (C) are represented in medial and posterior views. [file peerj-08-9359-s005.png]

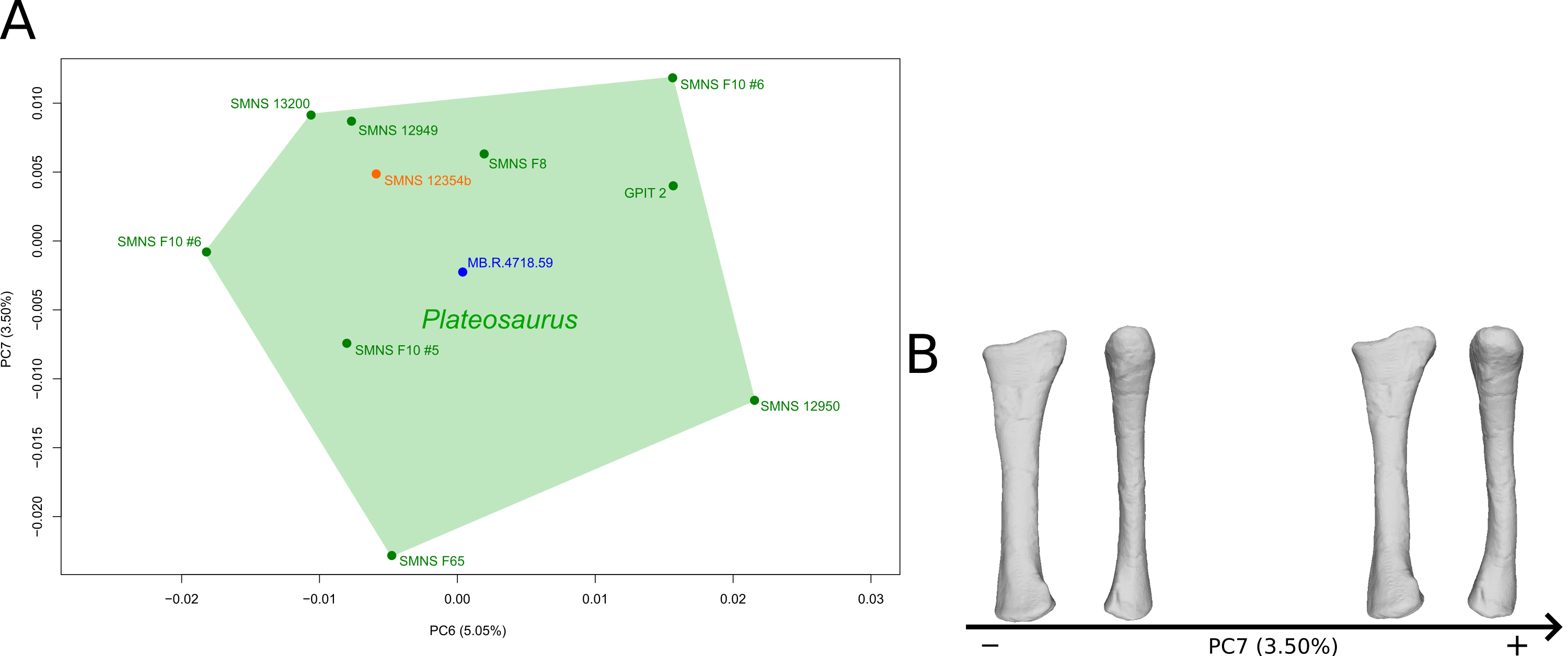

Supplement: Supplemental Information 6 — On the PCA plot (A), the green cluster represents the morphospace occupied by the genus Plateosaurus, the orange dot corresponds to the Efraasia specimen, the blue dot corresponds to the Ruehleia specimen. Extrema of shape changes along PC7 (B) are represented in medial and posterior views. [file peerj-08-9359-s006.png]

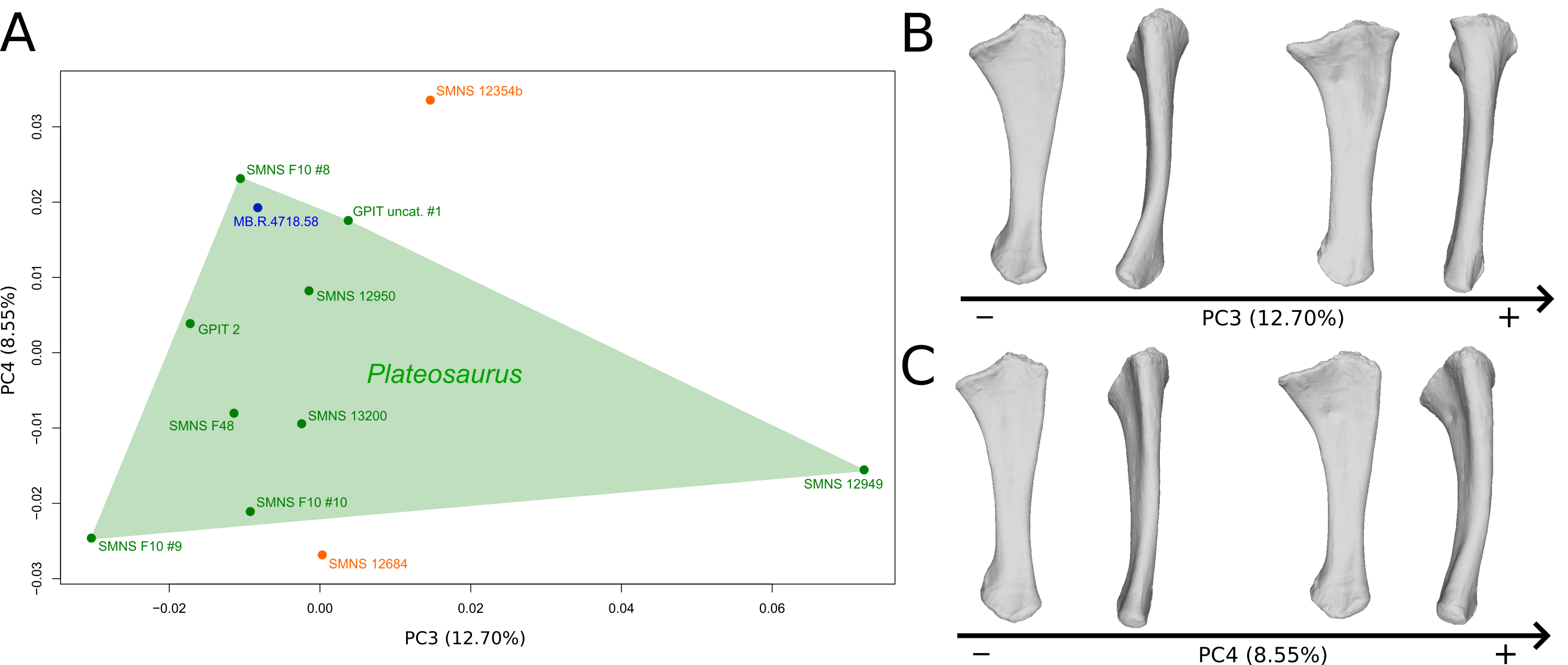

Supplement: Supplemental Information 7 — On the PCA plot (A), the green cluster represents the morphospace occupied by the genus Plateosaurus, the orange dots correspond to the Efraasia specimens, the blue dot corresponds to the Ruehleia specimen. Extrema of shape changes along PC3 (B) and PC4 (C) are represented in medial and posterior views. [file peerj-08-9359-s007.png]

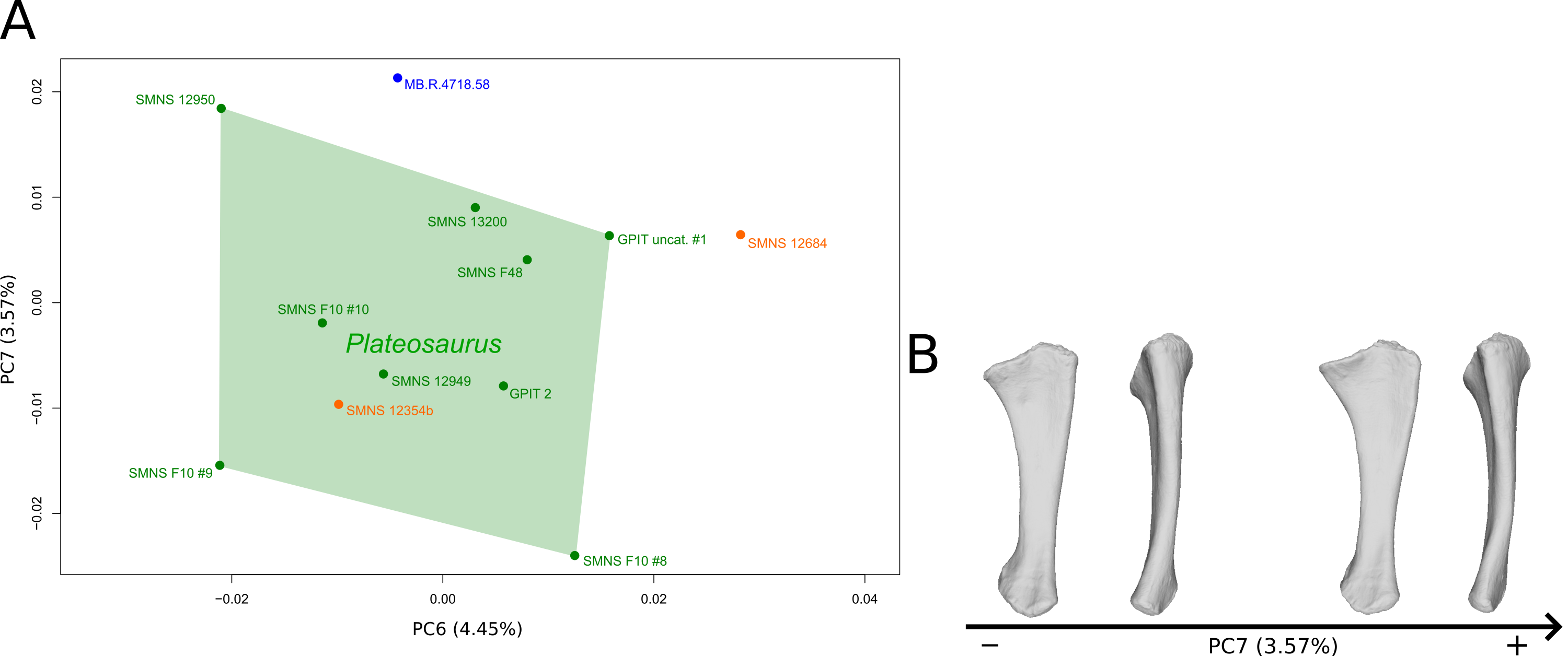

Supplement: Supplemental Information 8 — On the PCA plot (A), the green cluster represents the morphospace occupied by the genus Plateosaurus, the orange dots correspond to the Efraasia specimens, the blue dot corresponds to the Ruehleia specimen. Extrema of shape changes along PC7 (B) are represented in medial and posterior views. [file peerj-08-9359-s008.png]

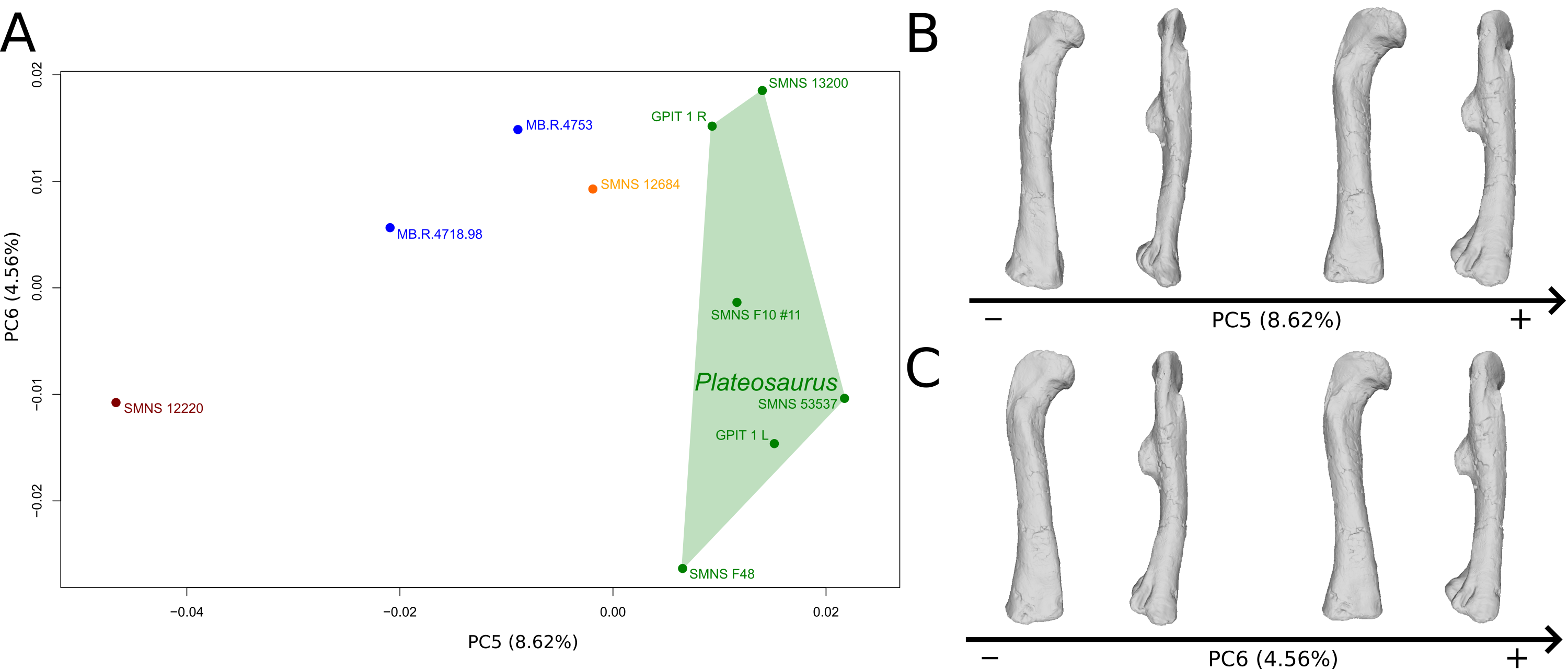

Supplement: Supplemental Information 9 — On the PCA plot (A), the green cluster represents the morphospace occupied by the genus Plateosaurus, the orange dot corresponds to the Efraasia specimens, the blue dots correspond to the Ruehleia specimen and the brown dot correspond to SMNS 12220. Extrema of shape changes along PC5 (B) and PC6 (C) are represented in anterior and lateral views. [file peerj-08-9359-s009.png]

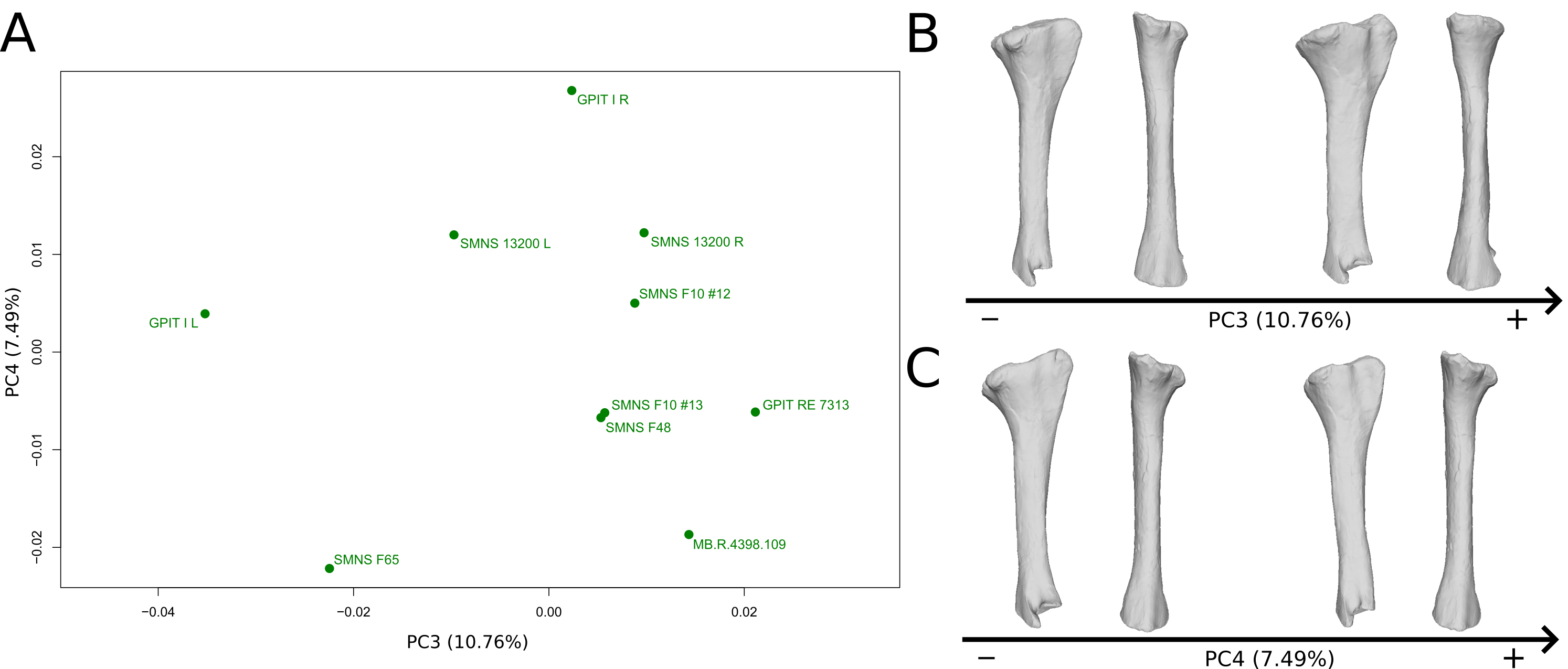

Supplement: Supplemental Information 10 — On the PCA plot (A), the green dots correspond to the specimens of the genus Plateosaurus. Extrema of shape changes along PC3 (B) and PC4 (C) are represented in lateral and posterior views. [file peerj-08-9359-s010.png]

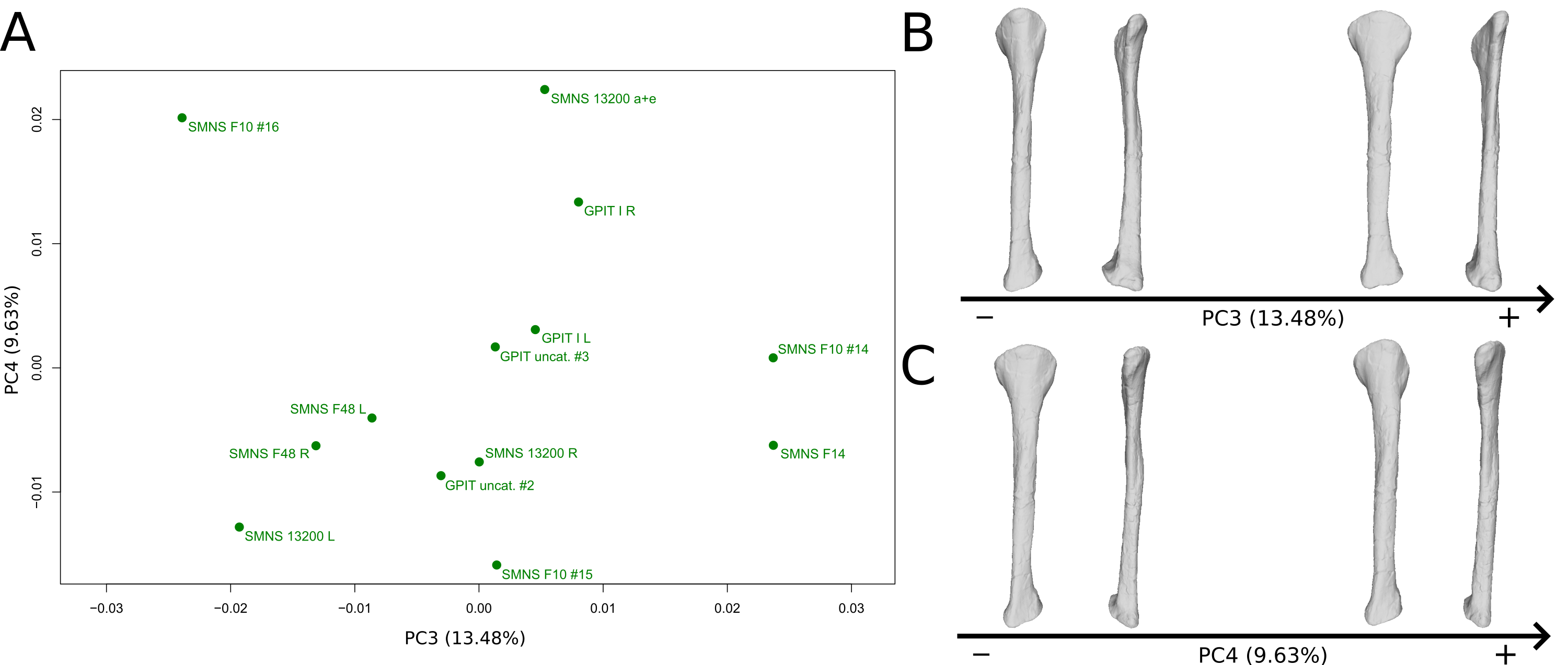

Supplement: Supplemental Information 11 — On the PCA plot (A), the green dots correspond to the specimens of the genus Plateosaurus. Extrema of shape changes along PC3 (B) and PC4 (C) are represented in lateral and posterior views. [file peerj-08-9359-s011.png]

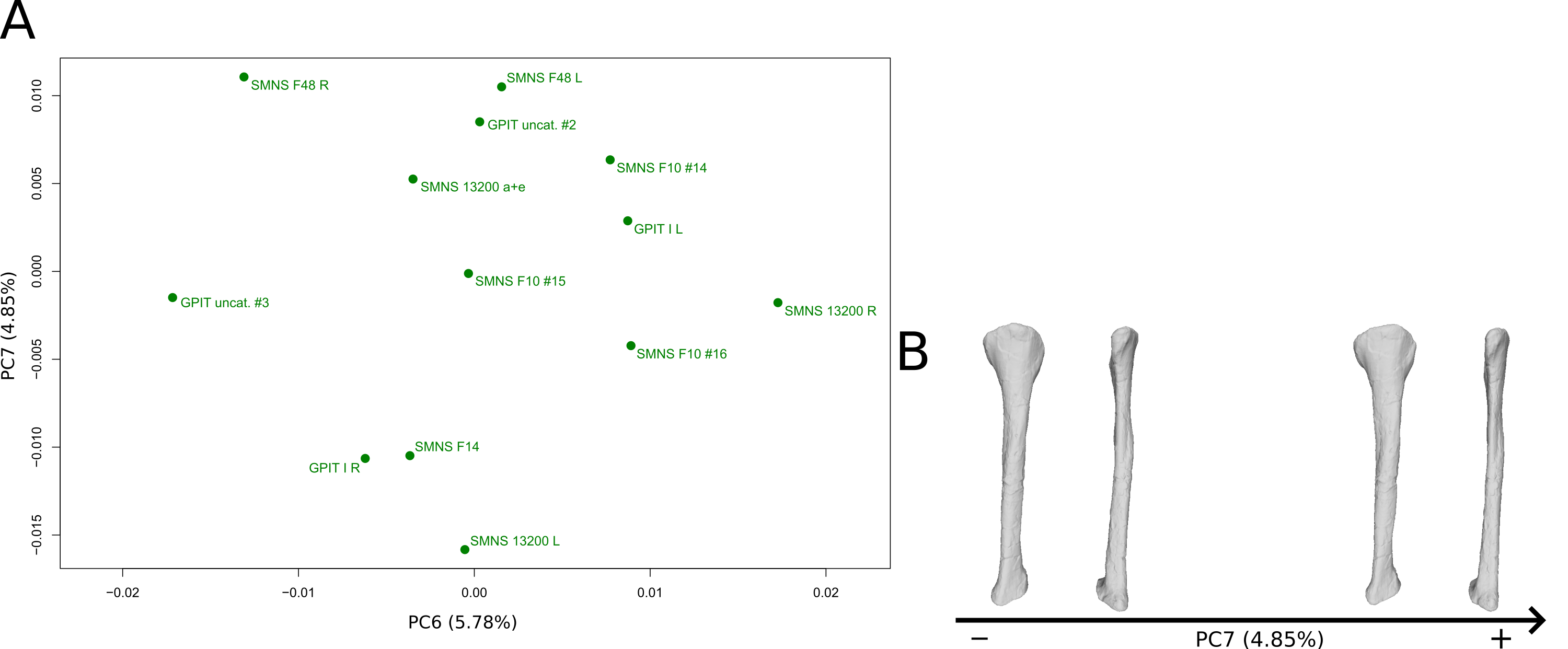

Supplement: Supplemental Information 12 — On the PCA plot (A), the green dots correspond to the specimens of the genus Plateosaurus. Extrema of shape changes along PC7 (B) are represented in anterior and lateral views. [file peerj-08-9359-s012.png]

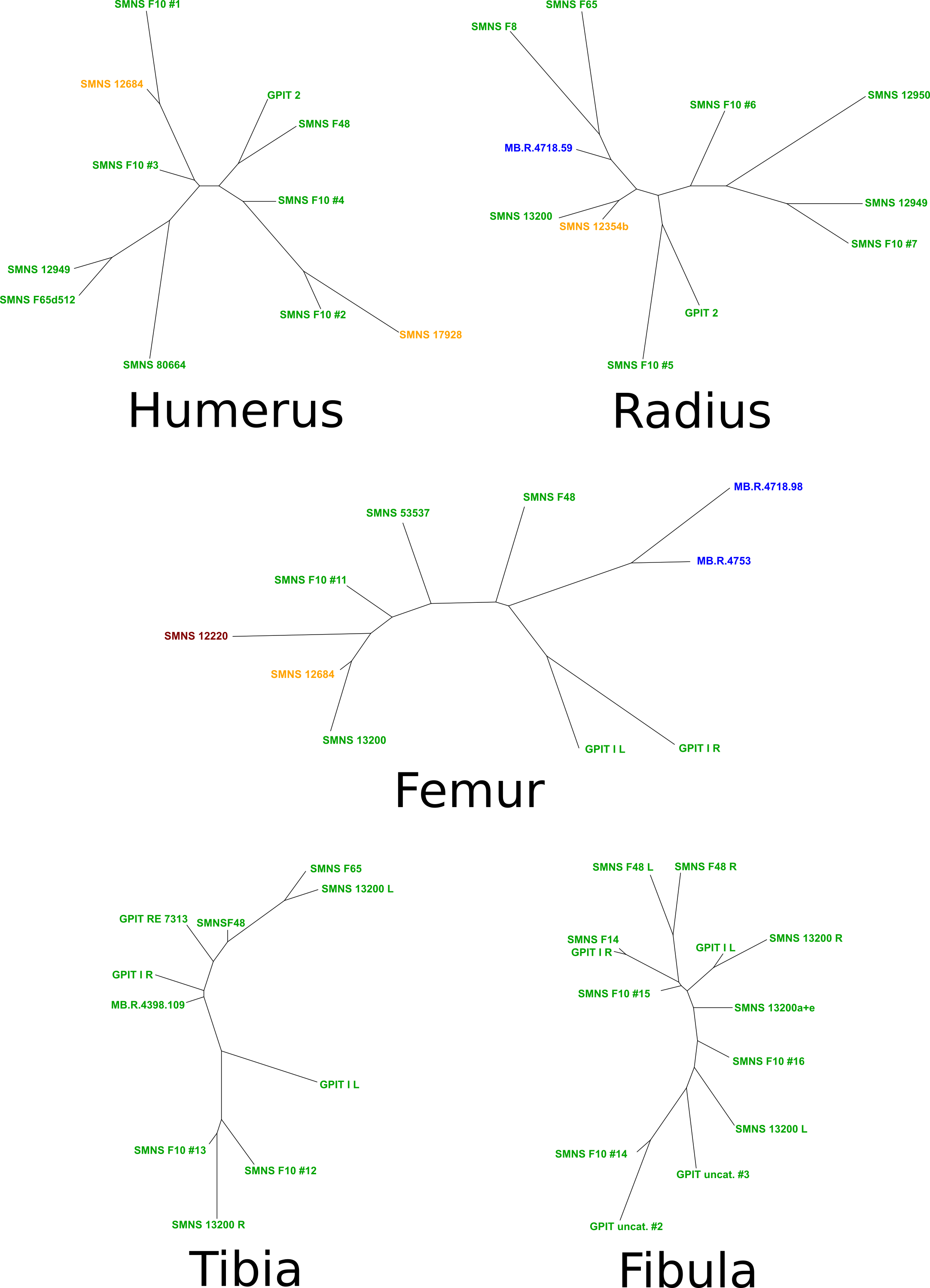

Supplement: Supplemental Information 13 — Analyses performed for the humerus (PCs 4-6), the radius (PCs 4-7), the femur (PCs 3,4 and 6), the tibia (PCs 5-6) and the fibula (PCs 2, 6 and 7). [file peerj-08-9359-s013.png]
