# Supplementary material for: Disentangling biological variability and taphonomy: shape analysis of the limb long bones of the sauropodomorph dinosaur Plateosaurus
Source: PeerJ. 2020 Jul 23;8:e9359. doi: 10.7717/peerj.9359 (PMC7382942; doi:10.7717/peerj.9359)
Supplement: Supplemental Information 14 — The following list enumerates with a brief description the main variations depicted in all the PCs (see results), associated with an interpretation in terms of taphonomic influence. The variation is categorized in three categories: obviously taphonomically ingluenced, ambiguous or biologically plausible. Only the PCs without any obviously taphonomically influenced variation are analyzed in terms of biological discussion. The biological discussion is supported by direct observation of the discrete (character state) or continuous (measurements) features on each analyzed specimen, recorded in tables in the second part of this appendix. [file peerj-08-9359-s014.docx]

**Appendix S1 – Summary of interpretations of PCs**

The following list enumerates with a brief description the main variations depicted in all the PCs (see results), associated with an interpretation in terms of taphonomic influence. The variation for each feature of each PC is categorized in three categories: obviously taphonomically ingluenced, ambiguous or biologically plausible. Only the PCs without any obviously taphonomically influenced variation are analysed in terms of biological discussion.

The biological discussion is supported by direct observation of the discrete (character state) or continuous (measurements) features on each analysed specimen, recorded in tables in the second part of this appendix.

**Part I – Interpretation of the influence of taphonomy on the described morphological features**

**Humerus**

**PC1**

#1: Deltopectoral crest: strong variation of orientation 🡪 This variation of orientation seems to be biologically unlikely, because it would imply a big constraint on the insertion of pectoralis muscle, on the medial side of the crest, responsible of forearm adduction. 🡪 Obviously taphonomically influenced variation

#2: Proximal end: strong mediolateral flattening variation and slight elevation of the area connected to the lateral tubecle 🡪 This pattern suggesting a strong mediolateral compression (representing an important variation of humerus morphology among the sample) of the proximal head seems to be biologically unlikely, because of the anatomical constraints imposed by the glenoid articulation with the scapulocoracoid 🡪 Obviously taphonomically influenced variation

#3: Shaft: strong anteroposterior compaction variation 🡪 This suggest a strong compression 🡪 Obviously taphonomically influenced variation

#4: Distal end: strong twist and flattening variation 🡪 The pattern described here suggest a strong humeral taphonomic modification of torsion, considering the intensity of the shape variation. The extremely important variation and the associated flattening of the other features of the bone give strong arguments to avoid to interpret the variation observed for this character. 🡪 Obviously taphonomically influenced variation

**PC2**

#5: Deltopectoral crest: strong variation of orientation (less important than in PC1), variation of thickness 🡪 This feature seems to be influenced by an anteroposterior compression of the bone strongly affecting the crest 🡪 Obviously taphonomically influenced variation

#6: Proximal end: overall and medial anteroposterior flattening variation, proximal elevation variation of lateral tubercle, proximolateral expansion variation of the medial tuberosity 🡪 These features seem caused by a strong compression distorting the location of the anatomical features. 🡪 Obviously taphonomically influenced variation

#7: shaft: sigmoidicity variation, flattening variation of the proximal part (linked to medial tuberosity variation) 🡪 As the proximal part is flattened, this variation seems biologically unlikely 🡪 Obviously taphonomically influenced variation

#8: Distal end: expansion variation of the condyles, slight twist variation 🡪 The changes are slighter than in PC1 and more biologically plausible. However, such variations, notably the twist, remains ambiguous at this taxonomic scale 🡪 Ambiguous variation

**PC3**

#9: Deltopectoral crest: Apex more or less projecting anteriorly, with more or less slope variation, slight orientation variation 🡪 These changes are more biologically plausible, as they do not seem to impact the muscles insertions on the crest. However, little variations of orientation still appear to be a little less biologically plausible than the others, and potentially affected by some taphonomy. 🡪 Ambiguous variation

#10: Proximal end: flattening variation of humeral head, with development variation of the lateral tubercle and elevation variation of the medial tuberosity 🡪 Strong compression of this part 🡪 Obviously taphonomically influenced variation

#11: shaft: Variation of sigmoidicity at midshaft 🡪 As this variation is relatively slight, do not prevent a biologically plausible insertion of muscles, and that the two morphs are seen in other non-sauropodan sauropodomorphs, we can assess that this variation is biologically likely. However, this feature can be accentuated by taphonomic deformations such as bendings or modification of torsions, as it can be seen in bones coming from the same specimen in morphologically similar taxa (e.g. the saurischian “*Ischisaurus cattoi*”, for which the type specimen have a sigmoid humerus and a straighter humerus, pers. obs.) 🡪 Ambiguous variation

#12: Distal end: expansion and flattening variation of the condyles and the intercondylar pit 🡪 Strong variation highly suggesting a compression of the intercondylar pit area 🡪 Obviously taphonomically influenced variation

**PC4**

#13: Deltopectoral crest: Slight proximodistal shift variation of the apex; flattening variation of the lateral side, variations of outline. 🡪 As the changes are slight and does not impact the orientation of the crest, they seem more biologically plausible 🡪 Most biologically plausible variation

#14: Proximal end: anteroposterior flattening variation 🡪 As the variation is slight and does not involve strong displacements of the features of the humeral head, the variation is more biologically plausible than those seen in the previous PCs, but the anteroposterior flattening highlighted here still suggests a taphonomic compression pattern. 🡪 Ambiguous variation

#15: shaft: sigmoidicity variation 🡪 same as #11 🡪 Ambiguous variation

#16: Distal end: flattening variation of medial and lateral margins, depression variation of anterior and posterior margins 🡪 The changes are slight but the flattening of medial and lateral borders is taphonomically suspiscious, suggesting potential missings of the distal part. 🡪 Ambiguous variation

**PC5**

#17: Deltopectoral crest: slight changes of apex expansion 🡪 same as in #13 🡪 Most biologically plausible variation

#18: Proximal end: slight anteroposterior flattening variation 🡪 same as in #14 🡪 Ambiguous variation

#19: shaft: sigmoidicity variation of the midshaft and the proximal part of shaft 🡪 same as #11 🡪 Ambiguous variation

#20: Distal end: smooth twist variation of orientation🡪 same as in #8 🡪 Ambiguous variation

**PC6**

#21: Deltopectoral crest: relatively strong outline variations 🡪 same as in #13 🡪 Most biologically plausible variation

#22: Proximal end: slight flattening variation of anterior and posterior margins 🡪 The changes are smoother than those observed previously in this area and are therefore the most biologically plausible 🡪 Most biologically plausible variation

#23: shaft: robustness and sigmoidicity variation 🡪 Similarly as in #11, as the changes are slight and do not prevent plausible insertion of muscles, the variation seem biologically likely. Given their intensity, they seem to be more plausible biologically than those seen in the previous PCs 🡪 Most biologically plausible variation

#24: Distal end: roundness variation of the radial condyle 🡪 The changes are smoother than those observed previously in this area and are therefore the most biologically plausible 🡪 Most biologically plausible variation

**PC4, 5 and 6 are here considered as the most biologically plausible PCs. They are thus the only biologically discussed PCs.**

**Radius**

**PC1**

#1: proximal half: flattening variation 🡪 The variation is important, and suggest a relatively strong taphonomic compression 🡪 Obviously taphonomically influenced variation

#2: distal half: curvature variation 🡪 The variation is important, and suggest a relatively strong taphonomic bending 🡪 Obviously taphonomically influenced variation

#3: distal end: outline variation 🡪 The variation is relatively slight and do not involve strong anatomically aberrant variations, but the pattern can suggest also a taphonomic compression, which is concordant with the features #1 and #2 🡪 Ambiguous variation

**PC2**

#4: strong general flattening variation 🡪 The variation is extreme and is very likely caused by a strong taphonomic compression 🡪 Obviously taphonomically influenced variation

**PC3**

#5: proximal end: expansion variation, with a depth variation of the humeral cotyle 🡪 The shape variation is strong and affects the development of the proximal end, suggesting potentially breaks, abrasion or variation of preservation 🡪 Obviously taphonomically influenced variation

#6: shaft: robustness variation 🡪 The variation does not imply aberrant shape changes concentrated in a single axis (as we could expect with a taphonomic compression). The changes are not anatomically aberrant, they seem biologically likely 🡪 Most biologically plausible variation

#7: distal end: outline variation 🡪 same as in #5 🡪 Obviously taphonomically influenced variation

**PC4**

#8: proximal end: expansion variation, hourglass shaped 🡪 The shape changes are slight but possibly suggest variations linked to breaks, abrasion or variation of preservation 🡪 Ambiguous variation

#9: shaft: distal robustness variation 🡪 same as in #6 🡪 Most biologically plausible variation

#10: distal end: slight posterior development and orientation variation 🡪 The variation is relatively slight and do not involve strong anatomically aberrant variations 🡪 Most biologically plausible variation

**PC5**

#11: shaft: robustness variation 🡪 same as in #6 🡪 Most biologically plausible variation

#12: shaft: slight anteroposterior curvature variation 🡪 The variation is relatively slight and do not involve strong anatomically aberrant variations 🡪 Most biologically plausible variation

#13: distal end: slight twist variation 🡪 same as in #10 🡪 Most biologically plausible variation

**PC6**

#14: shaft: robustness variation 🡪 same as in #6 🡪 Most biologically plausible variation

#15: proximal end: slight development and flattening variation 🡪 same as in #12 🡪 Most biologically plausible variation

#16: distal end: slight flattening and orientation variation 🡪 same as in #8 🡪 Ambiguous variation

**PC7**

#17: shaft: robustness and curvature variations 🡪 same as in #6 🡪 Most biologically plausible variation

#18: proximal end: outline and curvature variations 🡪 same as in #12 🡪 Most biologically plausible variation

#19: distal end: outline and curvature variations 🡪 same as in #12 🡪 Most biologically plausible variation

#20: distal half: slight posterior curvature variation 🡪 same as in #12 🡪 Most biologically plausible variation

**PC4, 5, 6 and 7 are here considered as the most biologically plausible PCs. They are thus the only biologically discussed PCs.**

**Ulna**

**PC1**

#1: strong mediolateral general flattening variation 🡪 The observed pattern is unambiguously taphonomic, with a general strong flattening probably originating from a compression 🡪 Obviously taphonomically influenced variation

**PC2**

#2: strong mediolateral general flattening variation 🡪 Same as in #1 🡪 Obviously taphonomically influenced variation

#3: proximal end: strong curvature variation 🡪 The shape changes are here unambiguously taphonomic, resulting from the general flattening, associated to a bending of the shaft near the proximal end 🡪 Obviously taphonomically influenced variation

#4: distal end: acuteness and twist variation 🡪 The changes are strong, probably resulting respectively from a break or an abrasion, and from the general flattening (resulting in a variation of torsion of the distal end) 🡪 Obviously taphonomically influenced variation

**PC3**

#5: mediolateral general flattening variation 🡪 Same as in #1 🡪 Obviously taphonomically influenced variation

#6: proximal end: expansion variation of the olecranon and the anterior process, with a strong variation of concavity on the medial side 🡪 The anterior expansion and the concavity are unambiguously taphonomic, originating from a medio lateral compression, extremely marked on the medial side of the proximal part of the shaft. 🡪 Obviously taphonomically influenced variation

#7: shaft: straigthening of the shaft 🡪 The shape changes are plausible biologically, but remain strong, and seem influenced by the general flattening and the variation of concavity 🡪 Ambiguous variation

#8: distal half: twist variation associated with a flattening and expansion variation 🡪 It suggests a taphonomic modification of torsion of this area, asssociated with a compression or maybe a break 🡪 Obviously taphonomically influenced variation

**PC4**

#9: shaft: relatively intense curvature variation 🡪 The change is sufficiently slight to be biologically plausible, but can also possibly suggest a mediolateral compression🡪 Ambiguous variation

#10: proximal end: development variation of the anterior process 🡪 Although the shape change is biologically plausible, breaks, abrasion and differences of preservation could have affected the development of the anterior process 🡪 Ambiguous variation

#11: proximal end: development variation of the olecranon and the lateral process 🡪 The variation is relatively slight and do not involve strong anatomically aberrant variations 🡪 Most biologically plausible variation

#12: distal end: slight variation of expansion and orientation 🡪 same as in #11 🡪 Most biologically plausible variation

**PC5**

#13: shaft: curvature variation (less intense than in PC4), slight variation of robustness 🡪 The shape change is substantially slighter than in #9, and is not associated with anatomically aberrant variation in any areas. Thus, the variation seems substantially more plausible biologically. 🡪 Most biologically plausible variation

#14: proximal end: lateral margin elevation variation, humeral cotyle sharpness variation 🡪 The variation is slight and not anatomically aberrant, so that it is biologically likely. However, slight deformation or breaks, abrasion and preservation variation could have caused this kind of variation 🡪 Ambiguous variation

#15: proximal end: olecranon development variation (conversely to #14) 🡪 same as #11 🡪 Most biologically plausible variation

#16: distal end: acuteness (development) variation 🡪 same as #11 🡪 Most biologically plausible variation

**PC6**

#17: shaft: robustness and curvature variation 🡪 same as #13 🡪 Most biologically plausible variation

#18: proximal end: anterior process elevation variation 🡪 same as #10 🡪 Ambiguous variation

#19: proximal end: olecranon development variation (conversely to #18) 🡪 same as #11 🡪 Most biologically plausible variation

#20: distal end: general development variation 🡪 Although this kind of shape variation is plausible, the strong intensity of variation could also remain a relatively important break or a strong compression of the highly variating areas 🡪 Ambiguous variation

**PC7**

#18: robustness and slight sigmoidicity variation 🡪 same as #13 🡪 Most biologically plausible variation

#21: proximal end: apex of anterior process elevation variation 🡪 same as #10 🡪 Ambiguous variation

#22: proximal end: width variation, associated with a posteromedial margin variation 🡪 same as #10 🡪 Ambiguous variation

#23: distal end: general development variation 🡪 same as #20 🡪 Ambiguous variation

**PC4, 5, 6 and 7 are here considered as the most biologically plausible PCs. They are thus the only biologically discussed PCs**

**Femur**

**PC1**

#1: general mediolateral flattening variation 🡪 The deformation observed here is unambiguously taphonomic. The general flattening, resulting from a mediolateral compression crushed femoral head, the fourth trochanter and the distal epiphysis toward the shaft. 🡪 Obviously taphonomically influenced variation

#2: greater and lesser trochanter: shift of position 🡪 The location of the greater and the lesser trochanter, cannot be definitively discussed in terms of biological variation, because these features may be directly related to the taphonomic compression observed along this PC 🡪 Obviously taphonomically influenced variation

#3: distal half: mediolateral curvature variation of the shaft 🡪 As for #2, the variation is directly linked to taphonomic compression, as a curvature pattern occurring in this plan is anatomically aberrant 🡪 Obviously taphonomically influenced variation

**PC2**

#4: greater and lesser trochanter: variation of expansion and of the lateral projection 🡪 The important positional shift of trochanters and femoral head may have been caused by taphonomic compression, but may also be partly caused by some errors of restoration. 🡪 Obviously taphonomically influenced variation

#5: fourth trochanter: variation of position and orientation 🡪 Intense variation which suggests a mediolateral compression 🡪 Obviously taphonomically influenced variation

#6: femoral head: strong variation of orientation 🡪 same as in #4 🡪 Obviously taphonomically influenced variation

#7: distal end: strong mediolateral expansion variation, with slight lateral variation of orientation of the condyles 🡪 This strong variation does not involve anatomical aberrations such as mediolateral variations of orientation, but could suggest a taphonomic compression pattern 🡪 Ambiguous variation

**PC3**

#8: shaft: variation of curvature 🡪 This strong variation is occurring in the anteroposterior plan without any flattening, which is not anatomically aberrant, as it reminds the columnar shape of sauropod femora, and seem to reflect intergeneric variation (separating *Ruehleia* femora from the other ones), which thus seem biologically plausible 🡪 Most biologically plausible variation

#9: fourth trochanter: little variation of position and outline 🡪 the variation is relatively slight and do not involve anatomically aberrant changes 🡪 Most biologically plausible variation

#10: femoral head: very slight variation of orientation 🡪 same as #9 🡪 Most biologically plausible variation

#11: distal end: expansion variation and orientation (relatively to the shaft) 🡪 same as #8 (but here the variation is slighter) 🡪 Most biologically plausible variation

**PC4**

#12: shaft: variation of orientation of the distal part, with elongation of the anterior ridge along the shaft 🡪 same as #9 🡪 Most biologically plausible variation

#13: shaft: slight variation of curvature and circularity 🡪 This variation occurs in the anteroposterior plan (curvature) and is slight 🡪 Most biologically plausible variation

#14: shaft: flattening variation of the distal part 🡪 same as #7 🡪 Ambiguous variation

#15: fourth trochanter: strong variation of location and orientation 🡪 These shape changes could result from a relatively slight mediolateral compression, shifting the position and orientation of fourth trochanter. However, these features remain still biologically plausible, as the changes are slight. The location of caudofemoralis brevis and caudofemoralis longus muscles are preserved in a biologically plausible way (no variation of orientation of the trochanter) 🡪 Ambiguous variation

#16: femoral head: variation of expansion 🡪 The variation is marked but not anatomically aberrant. It is associated with a smooth variation of thickness/flattening, sufficiently slight to be interpreted as biologically plausible 🡪 Most biologically plausible variation

#17: distal end: slight variation of roundness and orientation (following the shaft) 🡪 same as #9 🡪 Most biologically plausible variation

**PC5**

#18: distal half: strong anteroposterior flattening variation, variation of distal condyles orientation 🡪 The strong pattern observed here suggests a strong compression, which is taphonomically aberrant 🡪 Obviously taphonomically influenced variation

#19: fourth trochanter: slight shift of position 🡪 same as #9 🡪 Most biologically plausible variation

#20: proximal end: femoral head & greater trochanter development variation 🡪 same as #9 🡪 Most biologically plausible variation

**PC6**

#21: shaft: variation of robusntess and circularity 🡪 same as #13 🡪 Most biologically plausible variation

#22: femoral head: flattening (thickness) variation 🡪 The variation occurs on the anteroposterior plan and is not associated with any mediolateral expansion variation that could be expected in the case of a taphonomic compression. Consequently, the variation seems highly biologically plausible 🡪 Most biologically plausible variation

#23: distal end: variation of outline and development of the condyles 🡪 same as #9 🡪 Most biologically plausible variation

**PC3, 4 and 6 are here considered as the most biologically plausible PCs. They are thus the only biologically discussed PCs.**

**Tibia**

**PC1**

#1: shaft: general mediolateral flattening variation 🡪 The two opposite extrema of this PC are unambiguously taphonomically influenced: the negative one is resulting from a lateral compression pushing the fibular condyle anteriorly and accentuating the torsion of the distal end toward anterior direction, while the positive one is resulting from a lateral compression pushing the fibular condyle posteriorly, accentuating the torsion of the distal end toward lateral direction. 🡪 Obviously taphonomically influenced variation

#2: proximal end: flattening variation, with variation of orientation of the fibular condyle and the cnemial crest 🡪 see #1 🡪 Obviously taphonomically influenced variation

#3: distal end: strong variation of orientation 🡪 see #1 🡪 Obviously taphonomically influenced variation

**PC2**

#4: strong general mediolateral flattening variation 🡪 unambiguously caused by a taphonomic compression 🡪 Obviously taphonomically influenced variation

**PC3**

#5: proximal end: strong variation of development, outline variation, cnemial crest variation of position, fibular condyle orientation variation, internal condyle expansion variation, medial side margin shape variation (straight to rounded). 🡪 The changes are strong and seem to reflect breaks, abrasion, deformations or preservation biases 🡪 Obviously taphonomically influenced variation

#6: shaft: variation of expansion of the posterior part 🡪 This variation could represent biological differences of robustness, but could also have been caused by taphonomic compression 🡪 Ambiguous variation

#7: distal end: anteroposterior variations of expansion 🡪 same as #6 🡪 Ambiguous variation

**PC4**

#8: proximal end: development variation, outline variations (conversely variating relatively to the development variation), cnemial crest development variation, expansion variation of the fibular condyle and the posterior margin 🡪 same as #5 🡪 Obviously taphonomically influenced variation

#9: shaft & distal end: slight twist toward anterior direction (positive side) 🡪 The changes are relatively slight and do not involve strong variation of the shape of the condyles, thus it is more biologically plausible that the features seen in the previous PCs 🡪 Most biologically plausible variation

**PC5**

#10: proximal end: development variation of the cnemial crest (anteriorly) and the internal condyle (posteriorly) 🡪 The variation of development seems to occur without strong variation of outline, which is more biologically likely that the variation seen in #5 and #8. The shape changes seem however still ambiguous 🡪 Ambiguous variation

#11: proximal end: anteroproximal orientation variation 🡪 As the variation is slight it is biologically likely, but the pattern also suggests potentially a taphonomic bending of the shaft near the proximal end🡪 Ambiguous variation

#12: shaft: circularity and posterior expansion variation 🡪 same as in #6 🡪 Ambiguous variation

#13: distal end: anterior development of the medial corner and posterior margin curvature variation 🡪 same as in #9 🡪 Most biologically plausible variation

**PC6**

#14 proximal end: mediolateral flattening 🡪 As the variation is slight it is biologically likely, but the pattern also suggests potentially a taphonomic flattening of the proximal end 🡪 Ambiguous variation

#15: proximal end: cnemial crest development variation 🡪 same as in #10 🡪 Ambiguous variation

#16: proximal surface and flattening variation 🡪 As the changes are slight the variation is biologically likely, but the observed pattern can also suggest abrasion, break or preservation biases 🡪 Ambiguous variation

#17: shaft: partial posterior shifts 🡪 The changes are smooth and do not involve anatomically aberrant variation, thus they are biologically likely 🡪 Most biologically plausible variation

#18: anterior part of distal surface more or less domed 🡪 same as #9 🡪 Most biologically plausible variation

**PC 5 and 6 are here considered as the most biologically plausible PCs. They are thus the only biologically discussed PCs.**

**Fibula**

**PC1**

#1: shaft: strong curvature variation 🡪 This strong variation suggests a mediolateral compression, mainly located on the shaft, affecting its curvature, but also the mediolateral orientation of the ends 🡪 Obviously taphonomically influenced variation

#2: proximal end: curvature variation of the medial and lateral margins toward the lateral side, associated with a development variation of the anterior part 🡪 This variation seems to suggest an anteroposterior compression provoking a bending of the shaft near the proximal end 🡪 Obviously taphonomically influenced variation

#3: distal end: flattening and proximal expansion variation 🡪 The variation suggests a strong anterior compression of the end 🡪 Obviously taphonomically influenced variation

**PC2**

#4: shaft: slight flattening and anterior expansion variation🡪 As these changes are relatively slight, they are more biologically plausible than those seen in PC1. However, the patterns observed here possibly suggest a taphonomic compression 🡪 Ambiguous variation

#5: proximal end: mediolateral flattening and anteroposterior expansion variation🡪 the shape changes are slighter than in #2, with no anatomically aberrant shape changes, and are hence more biologically plausible 🡪 Most biologically plausible variation

#6: proximal half: orientation variation 🡪 As the shape change is slight, it is more biologically plausible than in PC1. However, the pattern suggests possibly a slight alteration of the torsion of the shaft. 🡪 Ambiguous variation

#7: distal end: anterolateral and posterior margins development variation, associated with anteromedial projection development variation 🡪 The change is slight and very localized, which is more biologically likely than features in PC1. However, a compression of the anatomical feature could also explain this observation. 🡪 Ambiguous variation

**PC3**

#8: shaft: proximal half more or less pinched, associated with a lateral expansion variation 🡪 the variation suggests on the negative side an anterior compression of the bone, whereas on the positive side it suggests a mediolateral compression, which are both aberrantly taphonomic 🡪 Obviously taphonomically influenced variation

#9: proximal end: strong anterior flattening and mediolateral expansion variation 🡪 same as in #8 🡪 Obviously taphonomically influenced variation

#10: distal end: general development variation, relatively strong orientation variation of the anterior half 🡪 The variation is still relatively slight, hence biologically plausible, but the pattern also suggests an alteration of the bending of the distal part of the shaft near the distal end 🡪 Ambiguous variation

**PC4**

#11: shaft: anterior and posterior part antagonistic development variation 🡪 the shape changes are smooth, which seem more biologically likely that the changes observed in the previous PCs 🡪 Most biologically plausible variation

#12: proximal end: strong flattening of the anterior part, posterior development variation 🡪 The flattening variation is important, suggesting a strong compression or a break of the proximal part 🡪 Obviously taphonomically influenced variation

#13: distal end: anterior and posterior part antagonistic development variation 🡪 same as in #11 🡪 Most biologically plausible variation

**PC5**

#14: proximal half: mediolaterally flattening variation 🡪 The variation seems aberrantly taphonomic, suggesting a relatively strong compression 🡪 Obviously taphonomically influenced variation

#15: distal half: curvature variation 🡪 the variation is sufficiently slight to be biologically plausible, but could also suggest a relatively slight compression 🡪 Ambiguous variation

#16: ends 🡪 development variation 🡪 The flattening variation is important, suggesting a strong compression 🡪 Obviously taphonomically influenced variation

**PC6**

#8: shaft: anterior half anterior and posterior antagonistic development variation; associated with development variation of the distal half 🡪 same as in #11 🡪 Most biologically plausible variation

#9: proximal end: slight proximodistal twist variation, associated with a slight development variation of the anterior part 🡪 same as in #11 🡪 Most biologically plausible variation

#10: distal end: twist variations, associated with slight development variation of anteromedial projection 🡪 same as in #11 🡪 Most biologically plausible variation

**PC7**

#11: shaft: relative variation of the position of the iliofibularis insertion 🡪 same as in #11 🡪 Most biologically plausible variation

#12: proximal end: slight lateral twist and slight development variations 🡪 same as in #11 🡪 Most biologically plausible variation

#13: distal end: outline development variation 🡪 same as in #11 🡪 Most biologically plausible variation

**PC 2, 6 and 7 are here considered as the most biologically plausible PCs. They are thus the only biologically discussed PCs.**

**Part II – Tables recording the qualitative and quantitative morphological features discussed in the article for each specimen of each analysis**

| **Humerus** | Deltopectoral crest outline | Shaft | Robustness index |
| --- | --- | --- | --- |
| SMNS 12949 | - Beginning soft  - Apex laterally relatively convex (?)  - Ending steep  - Presence of distal processus  - Apex anteriorly slightly curved and elongated  - Apex not prominent | - Relatively straight | 2.44 |
| SMNS 91296 (F10) #1 | - Beginning soft  - Apex laterally relatively convex  - Ending soft  - Absence of distal processus  - Apex anteriorly straight and compact  - Apex relatively prominent | - Relatively sigmoid | 3.06 |
| SMNS 91296 (F10) #2 | - Beginning steep  - Apex laterally convex  - Ending steep  - Absence of distal processus  - Apex anteriorly slightly curved and elongated  - Apex prominent | - Relatively sigmoid | 2.87 |
| SMNS 91296 (F10) #3 | - Beginning steep  - Apex laterally straight  - Ending steep  - Absence of processus  - Apex anteriorly straight and elongated  - Apex prominent | - Relatively sigmoid | 2.80 |
| SMNS 91296 (F10) #4 | - Beginning soft  - Apex laterally convex  - Ending soft  - Absence of distal processus  - Apex anteriorly straight and compact  - Apex prominent | - Relatively sigmoid (?) | 2.71* |
| SMNS 91306 ^x^ (F48) | - Beginning steep  - Apex laterally straight  - Ending steep  - Absence of distal processus  - Apex anteriorly straight and compact  - Apex not prominent (?) | - Straight | 2.54 |
| SMNS 91310 (F65d512) | - Beginning soft  - Apex laterally convex  - Ending steep  - Absence of distal processus (?)  - Apex anteriorly slightly curved and elongated  - Apex not prominent | - Straight | 2.44 |
| GPIT 2 ^x^ | - Beginning steep  - Apex laterally convex  - Ending soft  - Absence of distal processus  - Apex anteriorly slightly curved and compact  - Apex prominent | - Sigmoid | 2,47 |
| SMNS 80664 | - Beginning slightly steep  - Apex laterally convex  - Ending soft  - Presence of distal processus  - Apex anteriorly curved and compact  - Apex not prominent | - Sigmoid | 2.84 |
| SMNS 12684 ^x^ | - Beginning steep  - Apex laterally convex  - Ending steep  - Absence of processus  - Apex anteriorly straight and elongated *  - Apex prominent * | - Straight * | 2.69* |
| SMNS 17928 | - Beginning soft  - Apex laterally relatively straight  - Ending steep  - Absence of processus *  - Apex anteriorly straight and elongated *  - Apex prominent * | - Sigmoid | 2.68* |

| **Radius** | Robustness index | Shaft posterior margin curvature |
| --- | --- | --- |
| SMNS 12949 | 2.55 | - Slightly curved |
| SMNS 12950 | 3.19* | - Well curved |
| SMNS 13200 ^x^ | 2.93* | - Straight |
| SMNS 81914 (F8) | 2.81 | - Well curved (?) |
| SMNS 91296 (F10) #5 | 2.38 | - Nearly straight |
| SMNS 91296 (F10) #6 | 3.03 | - Well curved |
| SMNS 91296 (F10) #7 | 3.00 | - Well curved |
| SMNS 91310 (F65) | 3.05 | - Well curved |
| GPIT 2 ^x^ | 2.69 | - Straight |
| SMNS 12354b ^x^ | 2.81* | - Straight* |
| MB.R.4718.59 ^x^ | 2.60 | - Curved* |

| **Ulna** | Robustness index | Shaft curvature in posterior side | Distal end |
| --- | --- | --- | --- |
| SMNS 12949 | 2.32* | - Straight* | - Presence of lateral concavity  - Presence of medial concavity  - Anteriorly developed  - Posteriorly not developed * |
| SMNS12950 | 2.94* | - Well curved | - Presence of lateral concavity  - Medial border flat  - Anteriorly developed  - Posteriorly not developed |
| SMNS13200 ^x^ | 3.18 | - Relatively straight | - Lateral border flat  - Slight medial concavity  - Anteriorly developed  - Posteriorly not developed |
| SMNS 91296 (F10) #8 | 2.49 | - Well curved | - Presence of lateral concavity  - Presence of medial concavity  - Anteriorly developed  - Posteriorly not developed |
| SMNS 91296 (F10) #9 | 3.00 | - Relatively curved | - Presence of lateral concavity  - Medial border flat  - Anteriorly not developed  - Posteriorly not developed |
| SMNS91296(F10) #10 | 2.78 | - Relatively straight | - Lateral border flat  - Medial border flat  - Anteriorly not developed  - Posteriorly not developed |
| SMNS 91306 ^x^ (F48) | 2.57 | - Relatively straight | - Presence of lateral concavity  - Presence of medial concavity  - Anteriorly developed  - Posteriorly developed |
| GPIT 2 ^x^ | 2.94 | - Relatively curved | - Laterally flat  - Medially flat  - Anteriorly developed  - Posteriorly not developed |
| GPIT uncat. #1 | 2.32* | - Relatively curved* | - Laterally flat  - Medially flat  - Anteriorly not developed *  - Posteriorly not developed |
| SMNS 12354b ^x^ | 2.92* | - Relatively curved* | ? |
| SMNS 12684 ^x^ | 3.24* | - Straight* | - Laterally flat*  - Medially flat*  - Anteriorly not developed*  - Posteriorly not developed* |
| MB.R.4718.58 ^x^ | 2.56 | - Well curved | - Presence of a slight lateral concavity  - Presence of slight medial concavity  - Anteriorly extremely developed  - Posteriorly extremely developed |

| **Femur** | Shaft curvature | fourth trochanter outline | Robustness index | Eccentricity index | Femoral head index  L/W: |
| --- | --- | --- | --- | --- | --- |
| SMNS 13200 ^x^ | - Sigmoid | - Rounded with distal process | 2,75 | 1,06 | 1,48 |
| SMNS 91296 (F10) #11 | - Sigmoid | - Rounded without distal process | 3,01* | 0,87* | 1,54 |
| SMNS 91306*(F48) | - Slightly sigmoid* | - Angled without distal process? * | 2,90 | 0,81 | 1,97 |
| GPIT 1 ^x^ L | - Sigmoid | - Rounded with distal process? | 2,86 | 1,10 | 1,97 |
| GPIT 1 ^x^ R | - Sigmoid | - Rounded with distal process? * | 2,72 | 1,08 | 1,83 |
| SMNS 53537 | - Slightly sigmoid* | ? * (Angled with distal process on the right femur of the same specimen) | 2,43* | 1,45* | 1,80 |
| SMNS 12220 | - Sigmoid | - Rounded without distal process | 2,80* | 1,18* | 1,96* |
| SMNS 12684 | - Sigmoid | ? * | 2,44* | 0,64* | 1,78* |
| MB.R.4718.98 ^x^ | - Straight | - Angled with distal process | 2,98 | 1,14 | 1,42* |
| MB.R.4753 | - Straight | - Angled without distal process | 3,06 | 1,24 | 1,79 |

| **Tibia** | Robustness index |
| --- | --- |
| SMNS13200 ^x^ L | 2,74 |
| SMNS 13200 ^x^ R | 2,78 |
| SMNS 91296 (F10) #12 | 3,07* |
| SMNS 91296 (F10) #13 | 3,13* |
| SMNS 91306 ^x^ (F48) | 3,13 |
| SMNS 91310 (F65) | 2,66 |
| GPIT 1 ^x^ L | 2,58* |
| GPIT 1 ^x^ R | 2,70 |
| GPIT RE 7313 | 2,99* |
| MB.R.4398.109 | 2,77* |

| **Fibula** | Robustness index | Relative position of the insertion of the iliofibularis relatively to the proximal end | Proximal half twist | Anteromedial projection development |
| --- | --- | --- | --- | --- |
| SMNS 13200 ^x^ L | 4,67 | 0,38 | - Well twisted | - Well developped |
| SMNS 13200 ^x^ R | 4,41 | 0,37 | - Well twisted | - Well developped |
| SMNS13200a+e | 4,38 | 0,36 | ? | - Well developped? |
| SMNS 91296 (F10) #14 | 5,50* | ? | - Straight* | - Well developped* |
| SMNS 91296 (F10) #15 | 5,10* | ? | - Straight* | - Well developped |
| SMNS 91296 (F10) #16 | 4,74* | 0,37* | - Straight* | - Less developped |
| SMNS 91297 (F14) | 4,70 | 0,37 | - Twisted | - Well developped |
| SMNS 91306 ^x^ (F48) L | 5,01 | 0,37 | - Well twisted | - Less developped |
| SMNS 91306 ^x^ (F48) R | 4,98 | ? | ? | - Less developped? |
| GPIT 1 ^x^ L | 3,89* | 0,38* | - Straight? * | - Less developed |
| GPIT 1 ^x^ R | 4,38 | 0,36* | - Straight* | - Less developped |
| GPIT uncat. #1 | 4,91 | 0,38 | - Straight? | - Well developped |
| GPIT uncat. #2 | 5,02 | 0,37 | - Twisted? | - Well developped |

^x^ = specimen present in more than two analyses

* = Values or characters states highlighted in yellow are biased so that the measure or the state does not reflect the biological original information
